# Impact of Virgin Olive Oil and Phenol-Enriched Virgin Olive Oils on the HDL Proteome in Hypercholesterolemic Subjects: A Double Blind, Randomized, Controlled, Cross-Over Clinical Trial (VOHF Study)

**DOI:** 10.1371/journal.pone.0129160

**Published:** 2015-06-10

**Authors:** Anna Pedret, Úrsula Catalán, Sara Fernández-Castillejo, Marta Farràs, Rosa-M Valls, Laura Rubió, Núria Canela, Gerard Aragonés, Marta Romeu, Olga Castañer, Rafael de la Torre, Maria-Isabel Covas, Montse Fitó, Maria-José Motilva, Rosa Solà

**Affiliations:** 1 Research Unit on Lipids and Atherosclerosis, CTNS, CIBERDEM, Hospital Universitari Sant Joan, Servei de Medicina Interna, IISPV, Universitat Rovira i Virgili, Reus, Spain; 2 Cardiovascular Risk and Nutrition Research Group (CARIN, Regicor Study Group), CIBER de Fisiopatología de la Obesidad y la Nutrición (CIBEROBN), IMIM (Hospital del Mar Medical Research Institute), Barcelona, Spain; 3 Ph.D. Program in Biochemistry, Molecular Biology and Biomedicine, Department of Biochemistry and Molecular Biology, Universitat Autònoma de Barcelona (UAB), Barcelona, Spain; 4 Food Technology Department, UTPV-XaRTA, University of Lleida-AGROTECNIO Research Centre, Lleida, Spain; 5 Centre for Omic Sciences (COS), Universitat Rovira i Virgili, Reus, Spain; 6 Pharmacology Unit, Facultat de Medicina i Ciències de la Salut, Universitat Rovira i Virgili, Reus, Spain; 7 Human Pharmacology and Clinical Neurosciences Research Group, CIBER de Fisiopatología de la Obesidad y la Nutrición (CIBEROBN), IMIM (Hospital del Mar Medical Research Institute), Barcelona, Spain; 8 Universitat Pompeu Fabra (CEXS-UPF), Barcelona, Spain; University of Milan, ITALY

## Abstract

The effects of olive oil phenolic compounds (PCs) on HDL proteome, with respect to new aspects of cardioprotective properties, are still unknown. The aim of this study was to assess the impact on the HDL protein cargo of the intake of virgin olive oil (VOO) and two functional VOOs, enriched with their own PCs (FVOO) or complemented with thyme PCs (FVOOT), in hypercholesterolemic subjects. Eligible volunteers were recruited from the IMIM-Hospital del Mar Medical Research Institute (Spain) from April 2012 to September 2012. Thirty-three hypercholesterolemic participants (total cholesterol >200mg/dL; 19 men and 14 women; aged 35 to 80 years) were randomized in the double-blind, controlled, cross-over VOHF clinical trial. The subjects received for 3 weeks 25 mL/day of: VOO, FVOO, or FVOOT. Using a quantitative proteomics approach, 127 HDL-associated proteins were identified. Among these, 15 were commonly differently expressed after the three VOO interventions compared to baseline, with specific changes observed for each intervention. The 15 common proteins were mainly involved in the following pathways: LXR/RXR activation, acute phase response, and atherosclerosis. The three VOOs were well tolerated by all participants. Consumption of VOO, or phenol-enriched VOOs, has an impact on the HDL proteome in a cardioprotective mode by up-regulating proteins related to cholesterol homeostasis, protection against oxidation and blood coagulation while down-regulating proteins implicated in acute-phase response, lipid transport, and immune response. The common observed protein expression modifications after the three VOOs indicate a major matrix effect.

**Trial Registration:**

International Standard Randomized Controlled Trials ISRCTN77500181.

## Introduction

Olive oil (OO) is a food item typical of the Mediterranean diet, and several studies have revealed that it has a unique phenolic profile with specific biological properties. Results from the European EUROLIVE study [1] showed that an increase in plasma HDL cholesterol, and a decrease of LDL oxidation, took place in a direct relationship with the phenolic compound (PC) content of the OO administered. These findings provided evidence to recommend the use of PC-rich OO, i.e. virgin olive oil (VOO), in order to achieve beneficial effects improving lipid profile and conferring protection from oxidative damage. The difference of PC content between OO and VOO is due to the elaboration process (www.internationaloliveoil.org). To date, studies have been focused on hydroxytyrosol because it has been reported as being the most biologically active PC in OO [2]. In fact, the European Food Safety Authority considers that the health claim may be used only for OO which contains at least 5mg of hydroxytyrosol and its derivatives per 20g of OO [2]. As the phenolic concentration in most of the VOOs available on the market is too low to provide this daily amount of hydroxytyrosol, the enrichment of VOO with its own PCs could be a possible approach to assure the consumption without increasing caloric intake [3,4]. Furthermore, the enrichment of VOO with its own PCs could lead to a bitter organoleptic taste caused by the presence of secoiridoids (hydroxytyrosol and its derivatives; oleruopein and ligstroside aglycones) [5]. Moreover, in terms of biological effects, synergistic effects with stronger improvements have been reported when different PCs are combined in comparison to single treatments [6]. For these reasons, the enrichment of a VOO by complementing its own phenols with PCs of aromatic herbs has been done in order to improve its nutritional profile and organoleptic characteristics. Thyme could enhance perfectly these features of a phenol-enriched VOO because it is one of the richest sources of flavonoids, such as naringenin, eriodictyol and apigenin and phenolic acids such as rosmarinic acid, ferulic acid and caffeic acid [7,8].

Approximately one hundred different proteins, not considered to be apolipoproteins (APO), have been identified as being associated with the HDL particle [9] and linked to its cardioprotective properties. Analysis of the HDL protein cargo is, however, still in its early stages, and data concerning the HDL proteome as a biomarker for disease or functionality are limited. Moreover, studies with respect to the impact of dietary interventions on the HDL proteome are scarce [10], and the effects of OO PCs on the complex structure of the HDL protein cargo have not yet been evaluated.

In this context, we hypothesized that sustained consumption of PCs from VOO, or functional phenol-enriched VOO, could modify the HDL proteome, which in turn could be related to their cardioprotective benefits. The objective of the current study was to assess the impact on the HDL protein cargo of a dietary intervention supplemented with a VOO or two different functional VOOs enriched with their own PCs (hydroxytyrosol and its derivatives; oleuropein and ligstroside aglycones) or complemented with thyme PCs (flavonoids, monoterpens, and phenolic acids), in hypercholesterolemic subjects from the VOHF study.

## Materials and Methods

### Subjects and study design

The VOHF study aimed at assessing whether functional VOOs, enriched with their own PCs or with them plus complementary phenols from thyme, could have a nutraceutical effect on HDL lipoprotein functionality. The present clinical trial was conducted in accordance with the Helsinki Declaration and the Good Clinical Practice for Trials on Medical Products in the European Community. All participants provided written informed consent, and the local institutional ethics committees approved the protocol (Comité Ético de Investigación Clínica del Instituto Municipal de Asistencia Sanitaria; CEIC-IMAS 2009/3347/I) registered with the International Standard Randomized Controlled Trial register (www.internationaloliveoil.org; ISRCTN77500181). This trial was conducted according to extended CONSORT 2010 guidelines.

**Fig 1:**
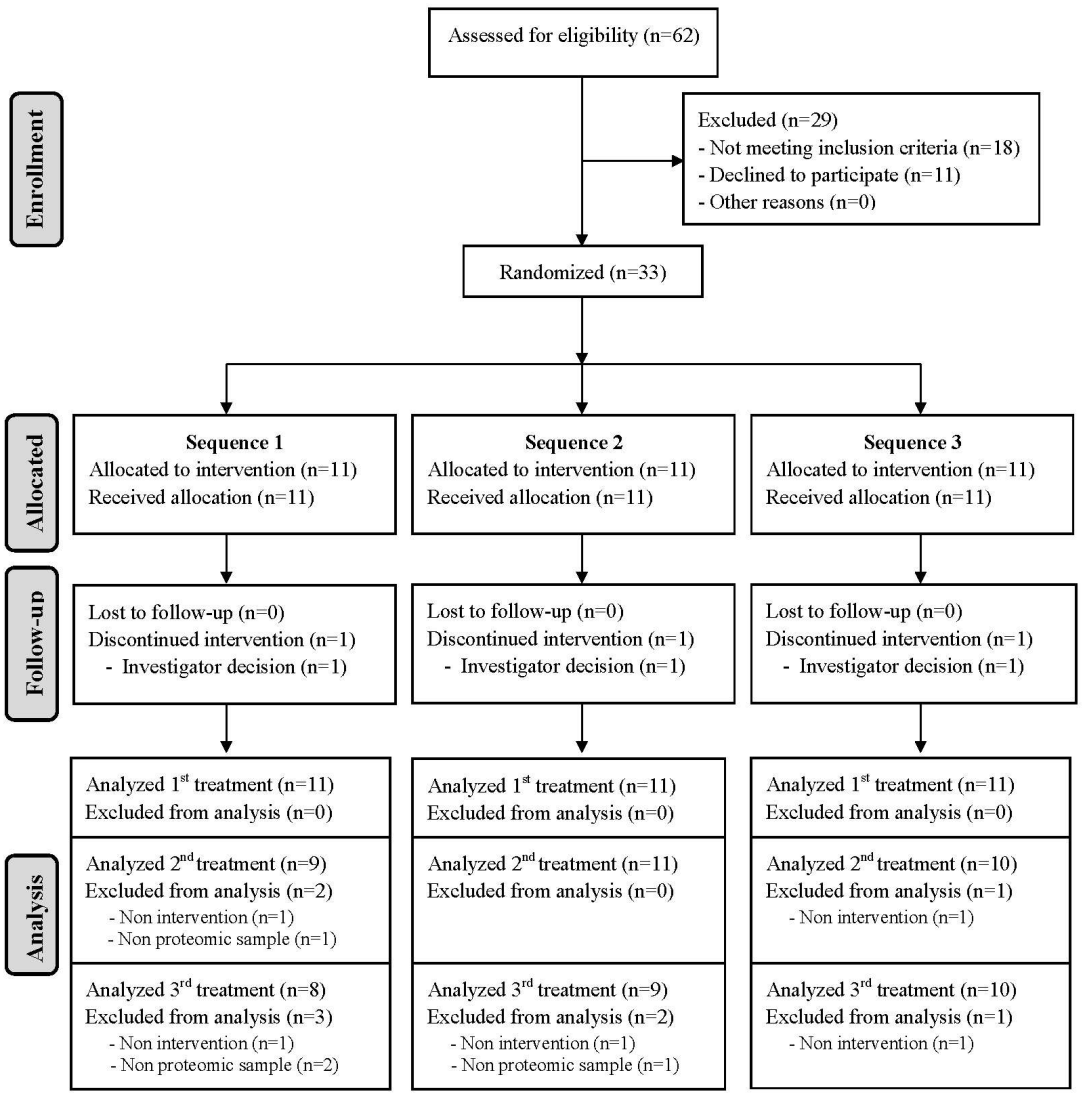
Participants Flow-chart based on post-interventions of HDL proteomics variable. Sequence 1: FVOO, FVOOT and VOO; Sequence 2: FVOOT, VOO and FVOO; Sequence 3: VOO, FVOO and FVOOT.

The VOHF study was a double-blind, randomized, controlled, crossover clinical trial with 33 hypercholesterolemic volunteers (total cholesterol>200 mg/dL; 19 men and 14 women), aged 35 to 80 year. Exclusion criteria included the following: BMI >35 Kg/m^2^, smokers, athletes with high physical activity (>3000 Kcal/day), diabetes, multiple allergies, intestinal diseases, or any other disease or condition that could worsen compliance. The study was conducted at IMIM- Hospital del Mar Medical Research Institute (Spain) from April 2012 to September 2012. The participants’ flow chart is described in Fig 1.

**Fig 2:**
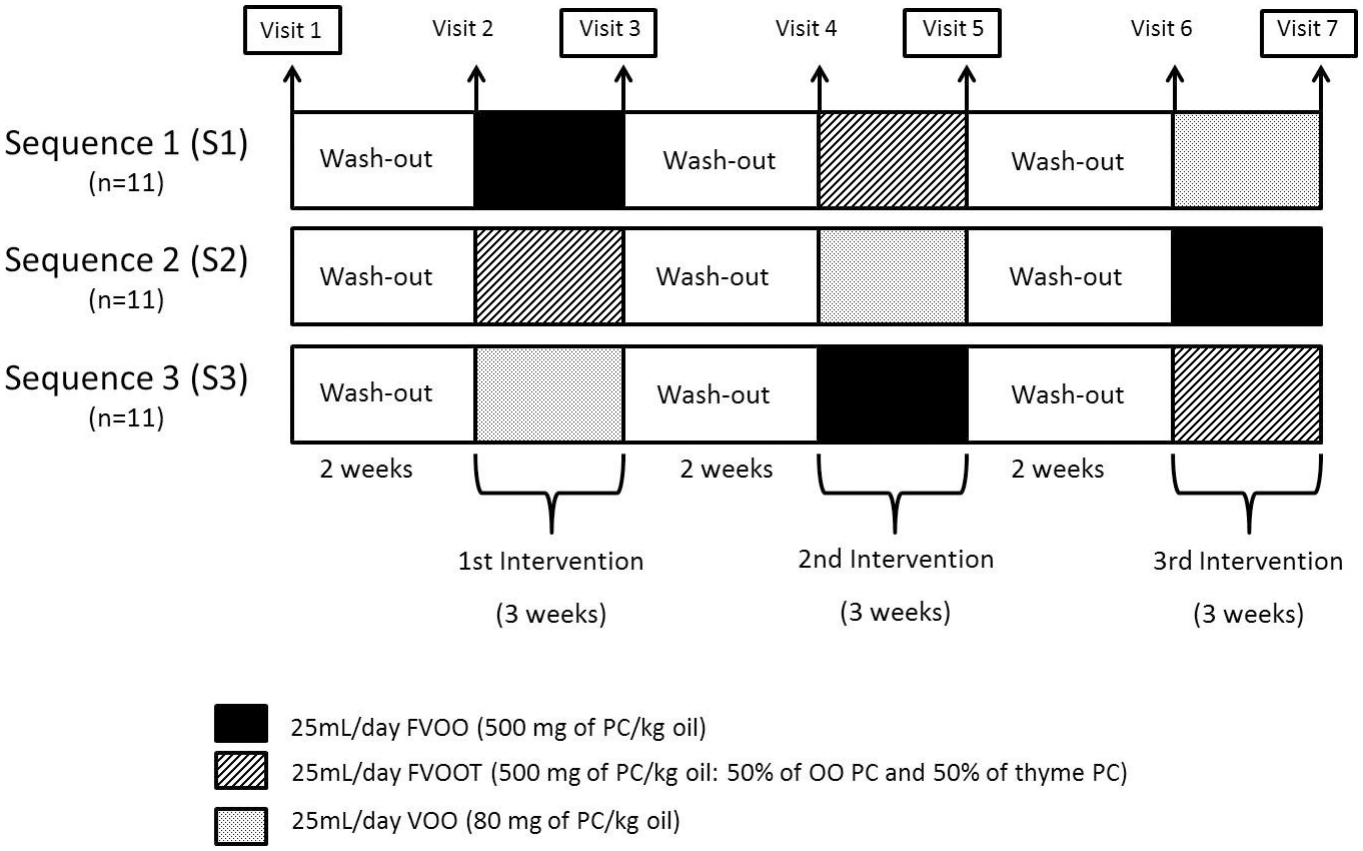
Experimental protocol for the intervention study. VOO: Virgin Olive Oil; FVOO: Functional Virgin Olive Oil enriched with its own PC; FVOOT: Functional Virgin Olive Oil enriched with its own PC plus complementary phenols from thyme. Blood collection for proteomic analysis: Visit 1, baseline; Visit 3, post-first intervention; Visit 5, post-second intervention; Visit 7, post-third intervention.

Subjects were randomly allocated to one of 3 sequences of administration of 25 mL/day of raw: a) VOO; 80 mg of PCs/kg oil, b) functional VOO enriched with its own PCs (FVOO; 500 mg of PCs/kg oil), and c) functional VOO enriched with its own PCs plus complementary phenols from thyme (FVOOT; 500 mg of PCs/kg; 50% of OO PCs and 50% of thyme PCs) (Sequence 1: FVOO, FVOOT and VOO, Sequence 2: FVOOT, VOO and FVOO, Sequence 3: VOO, FVOO and FVOOT) (Fig 2). The random allocation sequence was generated by a statistician, participant enrolment was carried out by a researcher, and participants’ assignment to interventions according to the random sequence was done by a physician. Due to the fact that all participants received each one of the three VOOs, restrictions such as blocking were unnecessary. Intervention periods were of 3 weeks and VOOs were consumed daily distributed among meals. There was a 2-week washout period prior to VOO interventions during which a common OO was consumed. A 3-day dietary record was administered to the participants at baseline and before and after each intervention-period. A nutritionist personally advised participants to replace all types of habitually consumed raw fats with the olive oils, and to limit their PC-rich foods consumption during the clinical trial. A set of portable containers with the corresponding 25 mL of VOO for each day of consumption were delivered to the participants at the beginning of each VOO administration period. The participants were instructed to return the containers to the center after the corresponding VOO consumption period in order to register the amount consumed. Moreover, the metabolites hydroxytyrosol sulfate, hydroxytyrosol acetate sulfate, thymol sulfate and hydroxyphenylpropionic acid sulfate were measured in 24-h urine in order to monitor the compliance after the intake of two phenol-enriched VOO [11]. Furthermore, concentration of the different phenolic metabolites was determined in the HDL fractions of the all participants. The concentrations of the metabolites present in the HDL pools used for proteomics analyses (as described below) are shown in S1 Table.

### Preparation and characterization of VOOs

The procedure to obtain the phenolic extracts and the enriched oils has been previously described [7]. Briefly, VOO with a low phenolic content was used as a control condition in the intervention and as an enrichment matrix for the preparation of the two phenol-enriched olive oils. FVOO was enriched with its own PCs by adding a phenol extract obtained from freeze-dried olive cake collected from a commercial olive mill in the olive-growing area of Les Garrigues (Lleida, Catalonia, Spain). FVOOT was enriched with its own PC (50%) and complemented with thyme PC (50%) using a phenol extract made up of a mixture of olive cake and commercially available dried thyme (*Thymus zyguis)*. The phenolic extracts used for enrichment were obtained in the laboratory using an accelerated solvent extractor (ASE 100 Dionex, Sunnyvale, CA). S2 Table shows the PCs, the fat soluble micronutrients, and the fatty acid daily intake with 25mL of VOO, FVOO, and FVOOT. The phenolic profile of the VOO was analyzed by high-performance liquid chromatography coupled to tandem mass spectrometry (HPLC/MS/MS) using the method previously described [7]. Tocopherols and fatty acids in VOO were analyzed following the procedure described by Morelló et al. (2004) [12] and the carotenoid content was analyzed as described before by Criado et al. (2008) [13].

### Sample size and power analysis

The sample size of 30 individuals allows at least 80% power to detect a statistically significant difference among three groups of 3 mg/dL of HDL-C and a standard deviation of 1.9, using an ANOVA test and assuming a dropout rate of 15% and a Type I error of 0.05.

### Collection of blood samples

**Fig 3:**
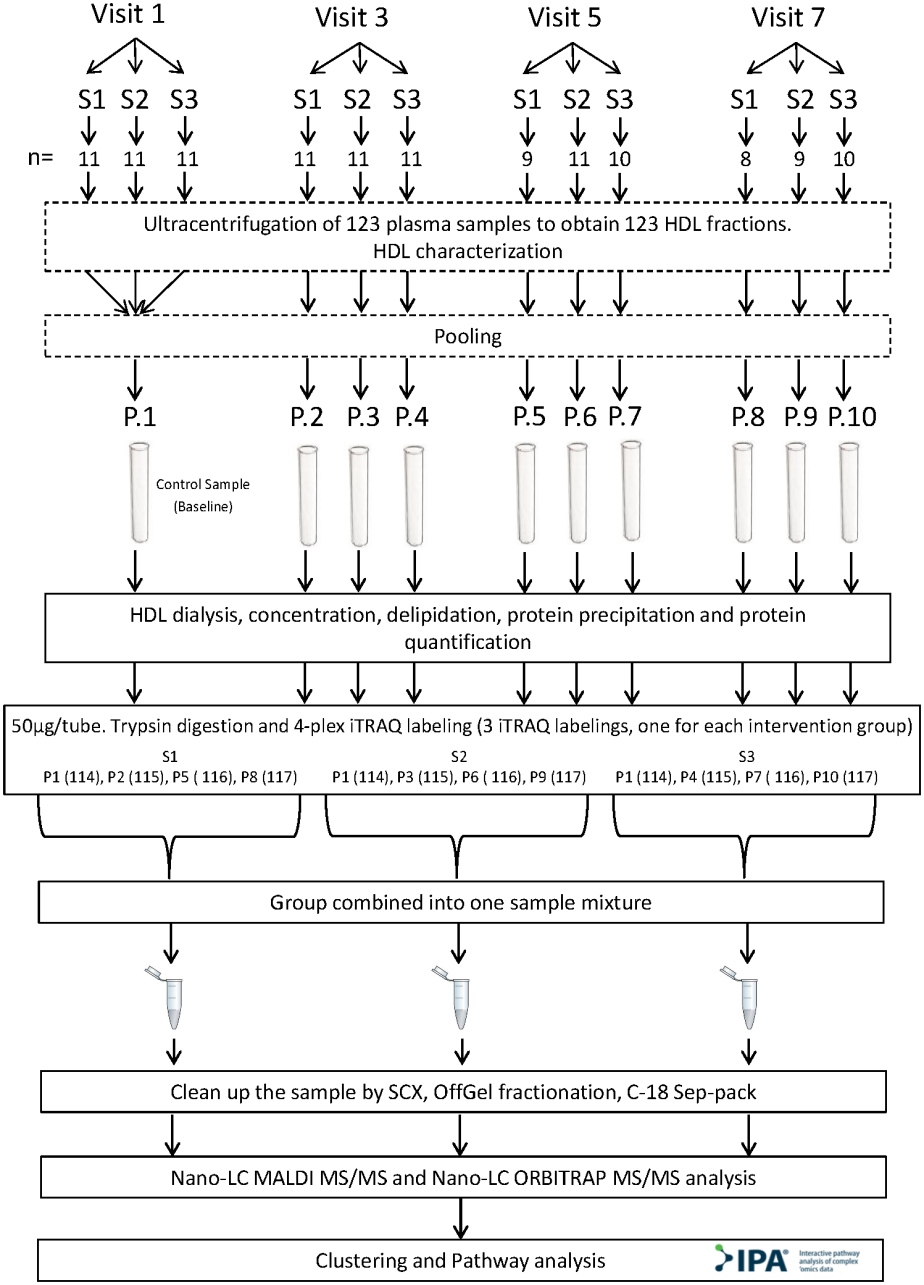
Proteomic study flow chart. S1: Sequence 1; S2: Sequence 2; S3: Sequence 3; HDL: P.: Pool; SCX: Strong Cation Exchange. Visits with n lower than 11 was due to loss of participants for the following reasons: dropout, did not participate in all interventions, did all the interventions but not all the tests, or sample loss during laboratory processing.

Fasting blood samples were taken from 33 participants before the first washout period (first visit-baseline) and after each VOO intervention period (visits 3, 5, and 7), through a catheter in an antecubital vein (Fig 2). Blood was collected in Vacutainer tubes with K_2_EDTA anticoagulant. Blood samples were centrifuged at 1 500 x*g* for 15 minutes and 2.8 mL of plasma were finally recovered. Protease Inhibitor Cocktail (PIC; Sigma-Aldrich, Tres Cantos, Spain) was added to plasma at a concentration 1/100 (1 μL of PIC for 100 μL of plasma). All samples were stored at -80°C until processing. A total of 123 plasma samples were used for proteomic analyses (33 participants per 4 visits minus 9 missing). See proteomic study flow chart (Fig 3).

### Human plasma HDL isolation

The HDL fraction (d = 1.036–1.21 g/mL) from each sample was isolated from 2.5 mL of plasma by sequential density ultracentrifugation in two steps using sodium bromide (NaBr; Sigma-Aldrich, Tres Cantos, Spain), as previously described by Havel RJ, et al. [14]. Sequential ultracentrifugation continues to be the procedure most commonly used for isolation of HDL in proteomic studies. To date, nearly all proteomic studies of HDL have utilized density gradient ultracentrifugation based on methods for the isolation of HDL from human plasma [15,16]. Briefly, the VLDL, IDL, and LDL, were removed by ultracentrifugation at 140 000 x*g* for 21 h at 10°C after adjustment of the plasma density to 1.063mg/mL with NaBr. The HDL fraction was obtained by adjusting the infranatant density to 1.21 mg/mL with NaBr prior to ultracentrifugation at 140 000 x*g* for 40 h at 10°C. 2 mL of HDL fraction were recovered and later stored at -80°C.

### HDL biochemical characterization

Apo A-I, Apo A-II and Apo B-100 were determined in each volunteer HDL fraction using immuno-turbidimetry assays (Horiba, Montpellier, France) in an autoanalyzer Cobas-Mira Plus (Roche Diagnostic System, Madrid, Spain). Total Cholesterol, Free Cholesterol, Phospholipids and Triglycerides were determined by enzymatic colorimetric techniques (Spinreact, Girona, Spain) also in the autoanalyzer Cobas-Mira Plus. Total protein was quantified in HDL fractions by the Bradford method (BioRad, Hercules, CA, USA). HDL fraction purity was assessed through the measurement of Apo B-100, almost all samples exhibited concentrations below the limit of detection (2 mg/dL).

### Proteomic sample preparation and quantitative analysis

For the proteomics studies (detailed in Supporting Information Data) the 123 isolated HDL samples were reduced to ten pools: 1 from first visit- baseline, 3 after third visit, 3 after fifth visit, and 3 after seventh visit (Fig 3). The pools were then dialyzed and delipidated by methanol/diethyl ether extraction, followed by a trichloroacetic acid/acetone protein precipitation before an in-solution tryptic digestion. Samples were subjected to an iTRAQ labeling for quantitative analysis, the resulting labeled peptides were separated according to their isoelectric point on an OffGel fractionator. Finally, two aliquots of the sample were analyzed by two different MS techniques, nano LC-MALDI MS/MS and nano LC-ORBITRAP-ESI MS/MS, to provide reliability and robustness (Fig 3). The files generated with the two mass spectrometers were combined in order to identify the HDL-associated proteins. The final identified proteins were required to present more than one peptide-spectrum match (PSM), or to have a minimum confidence score > 30% and coverage > 10%, in order to ensure accuracy in the assignment of protein identifications. In addition, intracellular and cell surface proteins were removed as being possible contaminants. In order to identify the differentially expressed proteins after interventions with respect to first visit- baseline, only samples analyzed by nano LC-ORBITRAP-ESI MS/MS were used due to the precision of this method. Relative expression levels were calculated for each protein as a ratio by Proteome Discoverer; biological replicates from the three iTRAQ sequences were then combined in an Excel spreadsheet and the mean of the ratios of all proteins calculated. Proteins with a differential expression of at least 0.8-fold change, or 1.3-fold change relative to baseline, were considered differentially expressed. In addition, a cut-off inferior to 0.5, or superior to 1.5, was applied to establish the most relevant protein expression changes observed after each intervention, defined as stronger effects. A detailed description of the methods and analysis is provided in the online **S1 Supporting Information**.

### Clustering and pathway analysis

Various bioinformatics tool were employed for the biological interpretation of the results. Proteins are referred to by their gene encode symbol.

Hierarchical clustering of protein expression data to investigate overall similarities of the proteome samples and identify the main biological functions involved was performed in web-tool STRING 9.1 (http://string-db-org). Ingenuity Pathway analysis (IPA; Ingenuity System Inc., Redwook, CA, USA, www.ingenuity.com) was used to analyze canonical pathways and protein networks involving the differentially expressed proteins for biological interpretation (Fig 3). Significance levels were assessed with Fisher’s exact tests (*p*<0.01). The differentially expressed proteins were overlaid with IPA-curated canonical pathways to explore possible metabolic and cell signaling pathways that were over- or under-represented by the experimentally determined genes. Specifically, we analyzed the proteins that overlapped the three VOO interventions in order to investigate potential common OO PCs mechanisms. In addition, possible connections between mapped genes were evaluated and graphical networks were algorithmically generated. Nodes representing genes and gene products were linked by biological relationships. Networks were ranked by a score that defines the probability of a collection of nodes being equal to or greater than the number in a network achieved by chance alone.

### Immunodetection assays

Apo A-I and Apo A-II, in the HDL pools, were determined using immunoturbidimetry assays (Horiba, Montpellier, France) in an autoanalyzer Cobas-Mira Plus (Roche Diagnostic System, Madrid, Spain). The quantification of haptoglobin and clusterin was performed using two commercial ELISA assays following manufacter’s instructions; the AssayMax Haptoglobin ELISA kit (AssayPro, MO, USA) and the Human Clusterin ELISA Kit (RayBiotech, GA, USA), respectively.

### Statistical analysis of biochemical data

The Kolmogorov-Smirnov test was used to verify the distributions of the variables. A paired Student’s t-test was employed for the comparison of paired and normally distributed variables. Wilcoxon signed-rank test was used for the comparison of paired and non-normally distributed variables. The level of statistical significance was set at *p* < 0.05. The possible interaction between the treatments and the treatment sequence (carryover effect) was assessed testing the period by treatment interaction-effect under a linear mixed model. All statistical analyses were performed with Statistical Package for the Social Sciences (SPSS) for Windows (20.0 version; IBM corp., Armonk, NY, USA).

## Results

### Participants’ characteristics

Table 1 summarizes the baseline characteristics of the study participants, significant differences were not observed among groups. No changes were observed in the main nutrients and medication intake throughout the study. The three VOOs were well tolerated by all participants and no adverse events were reported.

**Table 1:** Characteristics of the study participants at baseline.

Variable	Sequence 1 (n = 11)	Sequence 2 (n = 11)	Sequence 3 (n = 11)
Gender (male/female)	5/6	7/4	7/4
Age, years	54.91 ± 12.57	55.27 ± 11.88	55.45 ± 7.84
Body weight, kg	74.75 ± 16.80	74.60 ± 18.49	84.45 ± 17.74
BMI, kg/m2	25.63 ± 3.68	26.31 ±5.25	27.85 ± 4.71
SBP, mm Hg	125.09 ± 18.70	128.27 ± 16.69	130.45 ± 17.93
DBP, mm Hg	68.09 ± 13.53	72.27 ± 9.31	71.91 ± 13.43
Glucose, mg/dL	88.55 ± 11.63	93.00 ± 13.33	90.91 ± 10.53
Total cholesterol, mg/dL	228.36 ± 42.70	231.91 ± 32.70	218.82 ± 31.21
LDL cholesterol, mg/dL	150.38 ± 32.33	152.08 ± 28.46	142.26 ± 25.72
HDL cholesterol, mg/dL	52.78 ±11.75	52.96 ±12.82	53.39 ± 9.55
Tryglicerides, mg/dL	94.00 (75.00; 149.00)	119.00 (95.00; 168.00)	117.00 (81.00; 126.00)

Values expressed as mean ± standard deviation (SD) or median (25^th^ to 75^th^ percentile). Sequence 1 = FVOO, FVOOT and VOO; Sequence 2 = FVOOT, VOO and FVOO; Sequence 3 = VOO, FVOO and FVOOT. No significant differences between groups were observed. To compare means or medians among groups, ANOVA or Kruskal-Wallis test were performed, respectively; whereas χ2 and exact F-test, as appropriate, were computed to compare proportions. Abbreviations: SBP, systolic blood pressure; DBP, diastolic blood pressure.

### Biochemical characterization of HDL fractions

Biochemical characterization of HDL and also Apo A-I and HDL-c plasma levels segregated according to VOO intervention sequence are described in detail in S3 Table. In the HDL fraction and compared to baseline, significant increases in Apo A-I (p = 0.027; p = 0.021; p = 0.006, for VOO, FVOO, and FVOOT respectively), Apo A-II (p = 0.029; p = 0.019; p = 0.004, for VOO, FVOO, and FVOOT respectively), total protein (p = 0.073; p = 0.045; p = 0.037, for VOO, FVOO, and FVOOT respectively), total cholesterol (p = 0.033; p = 0.037; p = 0.005, for VOO, FVOO, and FVOOT respectively), phospholipids (p = 0.146; p = 0.021; p = 0.036, for VOO, FVOO, and FVOOT respectively) and total mass (p = 0.050; p = 0.014; p = 0.016, for VOO, FVOO, and FVOOT respectively) were observed after the three intervention periods. However, as a consequence of the increment of the different parameters of the HDL particle composition, the total mass of the HDL particle also increases and as a result, the composition of HDL expressed as percentage of total mass remains stable and non-significant changes were observed. Compared to baseline, significant increases were also observed in plasma Apo A-I and HDL-c after the three VOO intervention periods. However, no significant differences between the three VOO interventions, either in basal levels or in post interventions levels, were observed suggesting that in the present study, the enrichment of VOO with PC might not affect the ability of the VOO to increase different biochemical parameters and that the common matrix of the three VOO produced the key changes. We did not observe a carryover effect during the study.

### HDL proteomic results

#### Identification of HDL-associated proteins

A total of 155 HDL-associated proteins were initially identified by applying shotgun proteomics using Maldi and Orbitrap MS/MS to HDL fractions isolated by ultracentrifugation. Data were subsequently screened to ensure protein identification accuracy and 28 possible false-positives removed. The final list of HDL-associated proteins identified both at baseline and after interventions, was refined to 127 proteins (S4 Table). The majority (80/127) were consistent with well-established HDL-associated proteins determined in at least 3 different MS studies [9] and 32 had been previously identified by at least 1 MS study [9,17]. A total of 15 not previously referenced proteins were identified both at baseline and after interventions, although they do not show any modulation during interventions. These novel proteins are the following: Beta-Ala-His dipeptidase, BPI fold-containing family B member 1, Carbonic anhydrase 6, CD209 antigen, CD44 antigen, CD5 antigen-like, Ig heavy chain V-I region HG3, Ig lambda-1 chain C regions, Ig lambda-2 chain C regions, Ig lambda-7 chain C region, Indian hedgehog protein, Integrin alpha-2, Integrin beta-1, Multimerin-2, and Thymidine phosphorylase. Six well-established HDL-associated proteins were not detected: Apo O, Ceruloplasmin, Complement factor B, Inter alpha trypsin inhibitor 2, Plasma Kallikerein and Plasminogen [9].

#### HDL quantitative analyses

**Fig 4:**
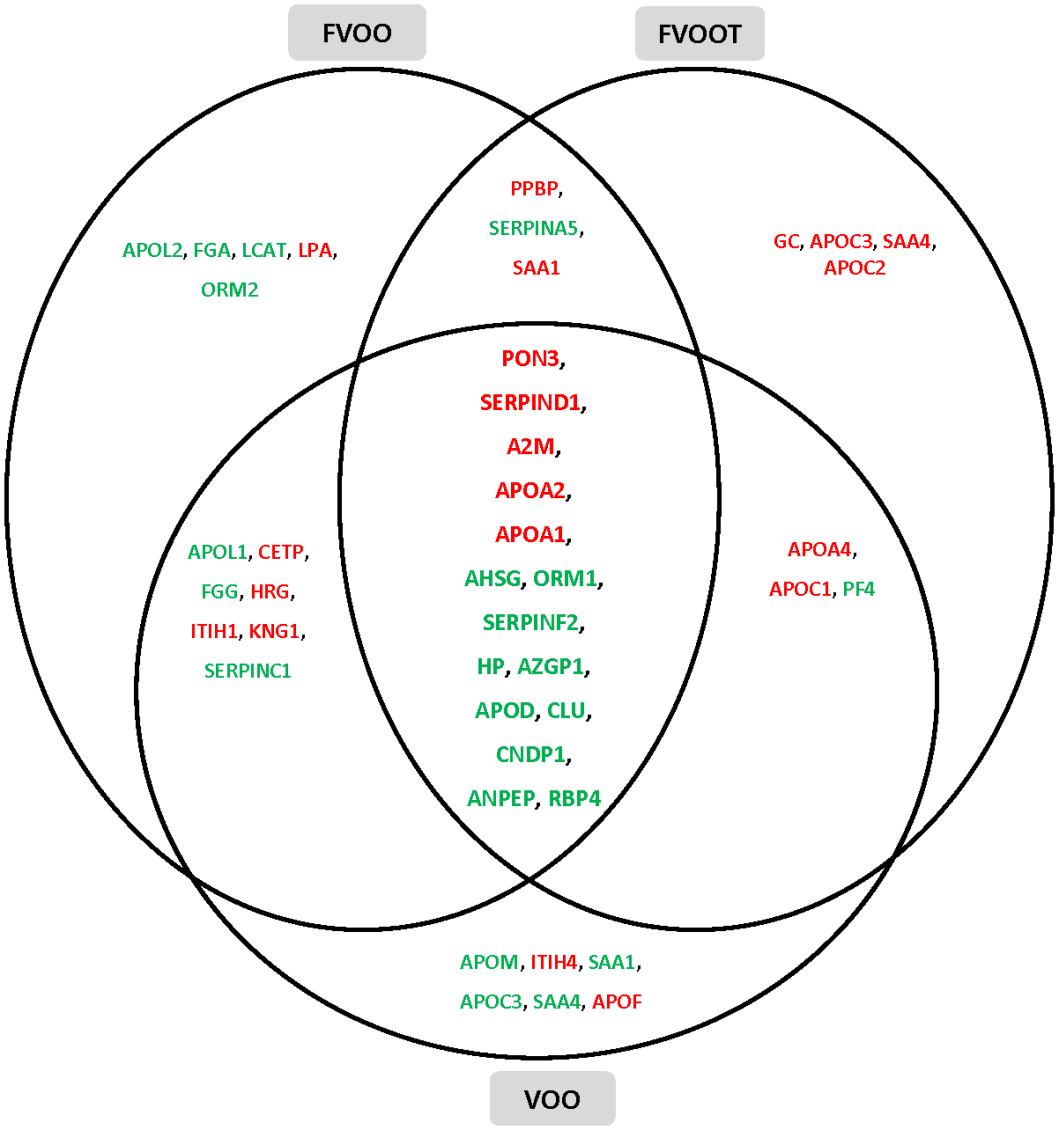
Venn diagram showing intersections of proteins differentially expressed after VOO, FVOO, and FVOOT interventions. Proteins are presented with their gene encode symbol. Red proteins: up-regulated; green proteins: down-regulated. VOO: Virgin Olive Oil; FVOO: Functional Virgin Olive Oil enriched with its own PCs; FVOOT: Functional Virgin Olive Oil enriched with its own PCs plus complementary phenols from thyme.

Compared to baseline values (first visit), the HDL protein cargoes of the differentially expressed proteins differed after the interventions according to the VOO received. The complete list of the proteins differentially expressed after each VOO intervention, and their principal biological functions, are shown in S5 Table. The proteins were associated with a broad range of biological functions, principally cholesterol homeostasis, lipid transport, acute-phase response, blood coagulation, immune response, protection against oxidation, and proteolysis. The proteins differently modulated after VOO, FVOO, and FVOOT interventions and associated with these main functions are represented in Fig 4. The overlapping among interventions indicates that 15 proteins were commonly up- or down-regulated after the three VOO interventions. These proteins were: Serum paraxonase/lactonase 3 (PON3), Apo A-II, Apo A-I, Apo D, Retinol binding protein 4 (RBP4), Heparin cofactor 2 (SERPIND1), zinc-alpha-2-glycoprotein (AZGP1), alpha-2-antiplasmin (SERPINF2), alpha-2-HS-glycoprotein (AHSG), clusterin (CLU), alpha-2-macroglobulin (A2M), haptoglobin (HP), alpha-1-acid glycoprotein (ORM1), Beta-Ala-His dipeptidase (CNDP1), and Aminopeptidase N (ANPEP). These 15 proteins that were observed to have the greatest expression modifications were identified with both MS techniques, nano LC-MALDI MS/MS and nano LC-ORBITRAP-ESI MS/MS. A protein-protein interaction network was generated for the 15 common, differentially expressed proteins using database and web-tool STRING 9.1 (S1 Fig). The common up-regulated proteins were related to cholesterol homeostasis, blood coagulation and protection against oxidation; the common down-regulated proteins were implicated in acute-phase response, lipid transport, immune response, and proteolysis. All proteins except for ANPEP and CNDP1, which are involved in proteolysis, appeared in the center of the functional network intersection indicating their key role in protein interactions. The information of these relevant proteins commonly regulated by the three VOO interventions was summarized in Table 2.

**Table 2:** Relevant proteins commonly differentially regulated by the three VOO interventions.

Gene Symbol	Protein Name	Principal Biological Function	VOO Fold Change	FVOO Fold Change	FVOOT Fold Change
**UP-REGULATED**					
APOA1	Apolipoprotein A-I	Cholesterol homeostasis	1.69	1.37	1.60
PON3	Serum paraoxonase/lactonase 3	Antioxidant protection	1.56	1.55	1.39
APOA2	Apolipoprotein A-II	Cholesterol homeostasis	1.45	1.40	1.34
SERPIND	Heparin cofactor 2	Blood coagulation	1.27	1.27	1.32
A2M	Alpha-2-macroglobulin	Blood coagulation	1.25	1.33	1.39
**DOWN-REGULATED**					
HP	Haptoglobin	Acute-phase response	0.56	0.80	0.65
CLU	Clusterin	Complement pathway and innate immune response	0.61	0.78	0.64
APOD	Apolipoprotein D	Lipid transport	0.61	0.71	0.68
AZGP1	Zinc-alpha-2-glycoprotein	Immune response	0.69	0.75	0.63
ORM1	Alpha-1-acid glycoprotein 1	Acute-phase response	0.75	0.53	0.58
SERPINF2	Alpha-2-antiplasmin	Acute-phase response	0.75	0.72	0.77
RBP4	Retinol-binding protein 4	Transport	0.78	0.78	0.77
ANPEP	Aminopeptidase N	Proteolysis	0.80	0.78	0.68
CNDP1	Beta-Ala-His dipeptidase	Proteolysis	0.82	0.69	0.72
AHSG	Alpha-2-HS-glycoprotein	Acute-phase response	0.84	0.80	0.76

Fold change > 1.3 denotes proteins up-regulated while fold change < 0.8 denotes decrease protein expression after the VOO interventions relative to the control-baseline. Gene symbol and principal biological function information were from UniProt database (http://www.uniprot.org/).

In addition to these 15 commonly modulated proteins, other specific protein expression changes were observed after each OO intervention. The stronger effects for each VOO were related to the following biological functions linked to atherosclerosis protection: transport, homeostasis cholesterol, antioxidant protection, blood coagulation, innate immune response, acute phase, and cellular adhesion. A stronger protein expression change was observed after VOO intervention in the following 6 proteins: Apo C-I, Apo A-I, Cholesteryl ester transfer protein (CETP), PON3, histidine-rich glycoprotein (HRG), and Ig heavy chain V-I region HG3, which were up-regulated while Ig lambda chain V-III region LOI was down-regulated. A stronger effect after FVOO was observed on 2 protein expression changes, PON3 and platelet basic protein (PPBP) which were up-regulated. Finally, a stronger up-expression change was noticed after FVOOT in the following 7 proteins: Apo A-I, Apo A-IV, Apo C-II, Apo C-III, serum amyloid A protein 4 (SAA4), afamin (AFM) and integrin beta-3 (ITGB3).

#### Confirmation of the proteomic results by immunodetection methods

**Fig 5:**
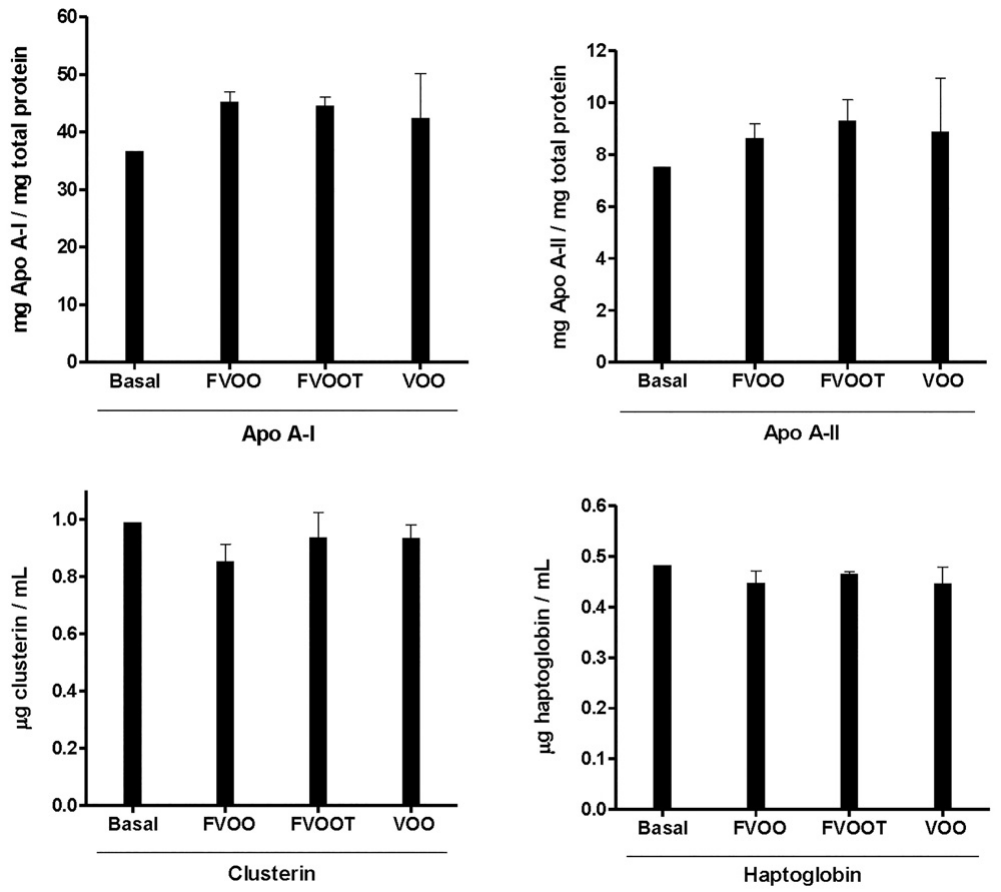
Expression levels of Apo A-I, Apo A-II, Clusterin and Haptoglobin. Expression levels of Apo A-I and A-II were measured by immunturbidimetry assays and clusterin and haptoglobin by ELISA assays in 10 HDL pools.

A panel of four proteins identified by MS analysis with changes after the three interventions, two up-regulated (Apo AI and Apo AII) and two down-regulated (Clusterin and Haptoglobin), were selected to confirm the changes in the protein expression that were observed in the proteomic analysis. These four proteins were quantified in the same 10 HDL pools used for proteomic analysis. Apo A-I and Apo A-II increases observed by proteomics were confirmed by immunoturbidimetry. The fold-changes observed in Apo A-I and Apo A-II measured by immunodetection were approximately 1.2 after the three VOO interventions compared to the basal value (Fig 5). Moreover, the results obtained for clusterin and haptoglobin quantifications measured by ELISA were also consistent with the decreases observed by proteomics with fold-changes of the order of 0.9 (Fig 5). The fold-changes observed using immunodetection methods were in the same way than those observed in proteomics analysis, even though they did not reach the same fold-change values. We observed an increase tendency for Apo AI and Apo AII proteins and a decrease tendency for Clusterin and Haptoglobin, in agreement with the MS findings, although these tendencies did not achieve statistical significance probably due to the low sample size. Moreover, these results confirm that proteomics analyses are more sensitive to detect minimum changes in HDL proteome.

#### Clustering and pathway analysis

**Fig 6:**
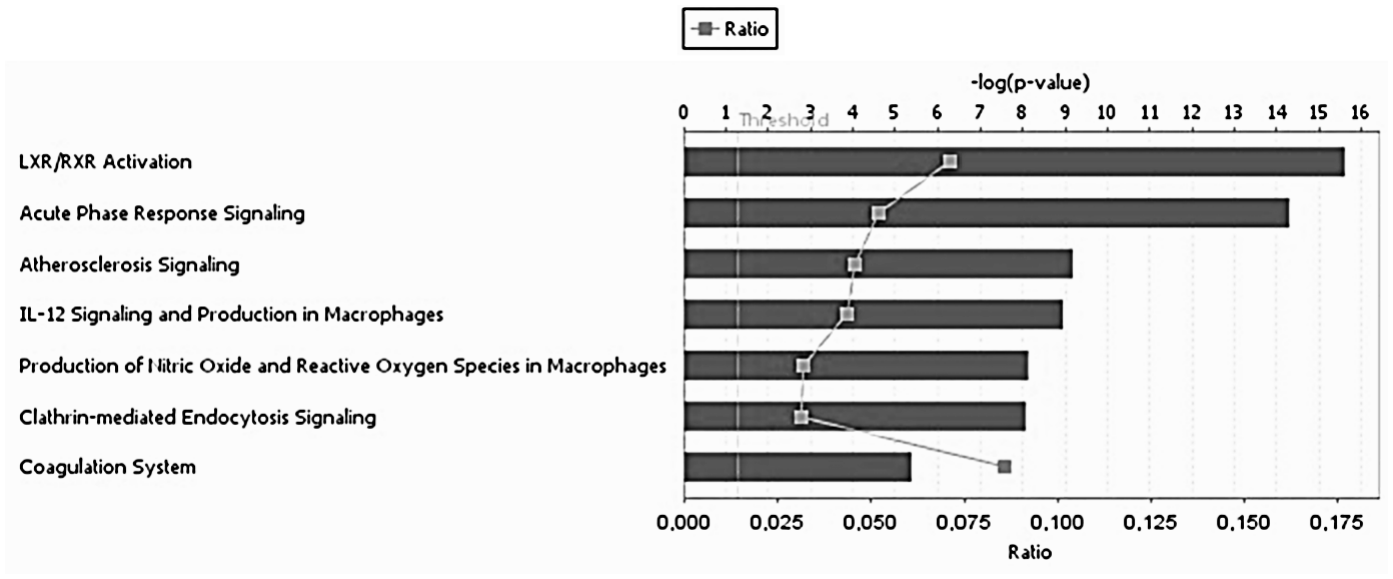
The top 7 signaling pathways that were significantly affected by the 15 common proteins differentially expressed after all VOO interventions. Significance levels were assessed with Fisher’s exact test (*p*<0.01). The pathways were ranked by *p* value. Blue bars indicate the negative log value (*p*-value). The ratio was calculated as the number of molecules in a given pathway that meet cutoff criteria divided by total number of molecules that make up that pathway.

**Fig 7:**
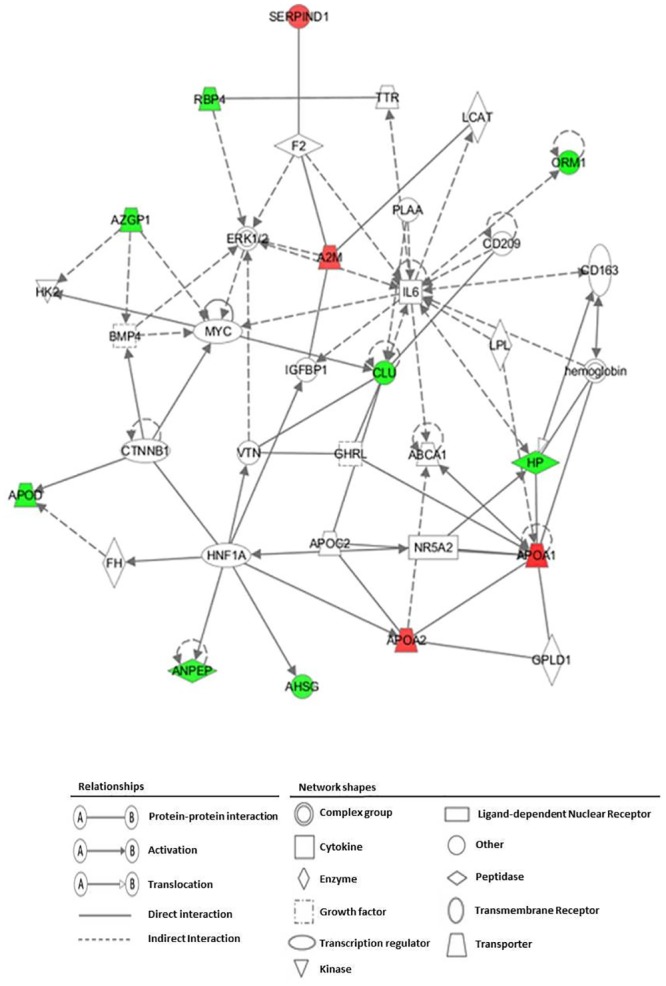
Top scored associated network generated by IPA describing common differentially expressed proteins after all VOO interventions. The top scoring significantly associated network was assessed with Fisher’s exact test (*p*<0.01). Proteins were presented with their gene encode symbol. The proteins indicated in red and green are those whose expression levels were significantly up- or down-regulated, respectively. Proteins indicated in white are those available in the IPA database, but not detected as differentially expressed in the present study. The shapes of the symbols denote the molecular class of proteins. Solid lines indicate direct molecular interactions, whereas dashed lines indicate indirect molecular interactions.

IPA analysis was performed to reveal the canonical pathways significantly affected (*p*<0.01) by OO phenols. The top 7 signaling pathways modified by the differentially expressed common proteins after all VOO interventions included: LXR/RXR activation, acute phase response signaling, atherosclerosis signaling, IL-12 signaling and production in macrophages, production of nitric oxide (NO) and reactive oxygen species (ROS) in macrophages, Clathrin-mediated endocytosis signaling, and coagulation system (Fig 6). Our results showed that OO phenols tended to have a strong effect on LXR/RXR activation (*p* = 2.39E-16; ratio = 0.071), followed by acute phase response signaling (*p* = 4.91E-15; ratio = 0.052). The highest scoring associated network generated by IPA, and the differentially expressed proteins common for all VOO interventions, are shown in Fig 7. The top scoring significantly associated network (score = 30) included 12 focused proteins and several associated genes, and was related to the following biological functions: lipid metabolism, small molecule biochemistry, and molecular transport. The nuclear receptor subfamily 5, group A, member 2 (NR5A2) and interleukin 6 (IL-6) were associated genes appearing in this network and representing the significantly affected top upstream regulators (*p*<0.01). NR5A2, which is implicated in the canonical pathway of FXR/RXR activation and the biological process of cholesterol homeostasis, was predicted from our results to be activated. Likewise, IL-6, which is involved in the acute phase response canonical pathway, was expected to be inhibited.

## Discussion

The present study revealed that, in hypercholesterolemic subjects, a supplementary consumption of 25 mL/day of raw VOO, FVOO, or FVOOT has an impact on the HDL proteome by changing the expression of a number of proteins with biological functions related to the cardioprotective function of the HDL particle. The greatest expression modifications were observed in 15 of the 127 proteins identified in the HDL fractions which were commonly up- or down- regulated after the three VOO interventions and might lead to a CVD protective HDL profile. A finding that encouraged us to highlight the effect of the fatty acid composition and the role of the PCs (lignans) of the common matrix of the three VOO tested on HDL remodeling. Moreover, IPA analysis revealed that several signaling pathways related to CVD were affected by OO PCs consumption. These data emphasize the key role of OO PCs in modifying pathways and conferring cardioprotective properties. PCs of VOO could be capable of transcriptional gene regulation inducing the down or up-regulation of proteins involved in functions related to cardiovascular risk [18,19]. Farràs et al.[20] observed how PCs from OO could exert an in vivo nutrigenomic effect on genes related to cholesterol efflux in humans. Moreover, PCs can modulate enzyme activities which may represent relevant antioxidant mechanisms by which dietary olive phenolic could have beneficial impact on cardiovascular health [21]. Phenol metabolites from olive have been observed to be incorporated to HDL particles where they could exert a local antioxidant protection and contribute to a functional enhancement of the HDL particle [22]. In concordance with these results, we observed that thyme phenols were also incorporated to HDL after FVOOT intervention and that olive phenol metabolites were higher after FVOO intervention compared to baseline and also to VOO. Furthermore, it has been recently reported that hydroxytyrosol contribute to the protection of endothelium reticulum (ER) stress which could cause unfolded or misfolded proteins and is considered as a new emerging risk factor for CVD. This newly described effect of hydroxytyrosol on ER stress could represent an alternative pathway through which olive oil phenols can modulate cell signaling and could impact HDL proteome and functionality [23].

To our knowledge, this is the first HDL proteomic study in humans that assesses the effects of PCs on the HDL protein cargo.

### Common effects of VOO interventions

Two well-known proteins related to cholesterol homeostasis, Apo A-I and Apo A-II, were significantly augmented after the three VOO interventions. Both proteins participate in reverse cholesterol transport and present anti-inflammatory and antioxidant properties [24,25]. The increase in Apo A-I concurred with the results observed by Solà et al. [26] in high CVD risk subjects after following a Mediterranean diet rich in VOO. PC could increase the expression and secretion of Apo-AI by regulatory mechanisms involved in cholesterol biosynthesis and metabolism [27]. The increment of Apo-AI observed could lead to a possible improvement of HDL functionality by enhancing HDL cholesterol efflux via ATP-binding cassette transporter A1 and HDL antioxidative properties, because Apo A-I is the major HDL component involved in these activities [28].

Two proteins related to blood coagulation, SERPIND1 and A2M, were up-regulated after the three VOO interventions thus increasing the cardioprotective activity of the HDL particle.

PON3, which is an antioxidant enzyme associated to HDL and with lactonase activity [29], was up-regulated after all VOO interventions. This effect could be indicative of an improvement in the oxidative status of the HDL particle promoted by the interventions. The effect of olive and thyme PCs on the main activities of these antioxidant enzymes should be further studied.

AHSG, ORM1, SERPINF2, and HP are acute-phase response proteins which were down-regulated after all VOO interventions. Acute-phase proteins are increased during inflammation and could be used as inflammatory biomarkers related to CVD [30,31]. Our results are in concordance with those of Santos-González et al. [32] who observed a decrease in acute-phase response proteins after a VOO diet in rats.

Plasma Apo D and RBP4, which have been reported to be up-regulated during CVD development [33,34], decreased after the three VOO interventions.

CLU, or Apo J, has been suggested to play a compensatory protective role by acting as an inflammatory modulator in myocardial infarction [35]. In the same way, AZGP1, has been observed to be up-regulated to counteract metabolic stress situations in humans [36]. Both proteins were down-regulated after all VOO interventions.

### Stronger effects of each VOO intervention

In addition to the commonly modulated proteins, specific strong effects on the HDL protein cargo were also observed after each intervention. For instance, after the intervention with VOO, Apo C-I, Apo A-I, CETP, and PON3 were markedly up-regulated. Among these changes the up-regulation of CETP is noteworthy. This protein facilitates the transport of cholesteryl ester from HDL to Apo B-100 containing lipoproteins, and its plasma activity has been suggested to be inhibited after a PC intervention [37]. To our knowledge, data concerning the effects of PCs on the expression and activity of CETP directly determined in HDL samples instead of plasma samples are limited. Changes in some immunoglobulins after VOO intervention were also reported. Immunoglobulins, which are secreted proteins related to innate immune response, could be plasmatic contaminants [17]. Nevertheless, the possibility that the HDL particle could act as a transporter of these proteins cannot be discarded and their role should be further studied.

From all the marked expression changes observed after the FVOO intervention, we would like to emphasize the up-regulation of the PON3.

We also highlight the strong up-regulation of Apo A-I, Apo A-IV, Apo C-II, and Apo C-III among the changes observed after the FVOOT intervention. Apo A-IV is mainly related to cholesterol homeostasis and oxidative protection, and has been associated with the cardioprotective properties of the HDL particle [38]. Although Apo C-III protein is present in HDL, it is also a major protein in VLDL. Chang et al. [39] showed positive associations between HDL- Apo C-III and the presence of CVD, suggesting that high levels of Apo C-III in the HDL particle may represent a class of dysfunctional HDL. Such results are, however, still controversial [39]. A redistribution of APOs between proatherogenic lipoproteins such as LDL to cardioprotective HDL could have been possible during the FVOOT intervention.

### Limitations and strengths

The pooling sample approach employed for the proteomic analysis, which was used to focalize primarily on the whole protein response and also to optimize the associated research expenses, constitutes the limitation of our study. Because of the methodological process used, the results of this work represent an exploratory analysis about the effect of a dietary intervention supplemented with a VOO or two different phenol enriched VOOs on the HDL proteome. However, the analysis is not sufficiently robust to draw conclusions about which of the three VOO tested has had the stronger effect on the HDL protein cargo. Future studies which analyze individual samples instead of pooling samples should be done to better assess the difference in the impact produced by the three types of VOO tested.

Moreover, HDL isolation is a key methodological point for proteomic analysis of HDL. Currently, the gold standard method for the HDL isolation is still under discussion and future attempts will be targeted towards improving isolation procedures [40]. Whilst sequential ultracentrifugation may alter HDL functionality, composition, and lead to a loss of lipid-poor Apo A-I it continues to be the most commonly used procedure in these studies. To date, nearly all proteomic studies of HDL have utilized density gradient ultracentrifugation based on methods for the isolation of HDL from human plasma [15]. However, the possibility exists that non-HDL plasmatic contaminants could have been present in the isolated HDL fraction.

One of the strengths of this study is its randomized and crossover design, which permitted the participants to ingest all VOO types. Moreover, the identification of the HDL-associated proteins was done by with two different MS techniques, Orbitrap and Maldi, which provide reliability and robustness to our identification results. Furthermore, it is important to note the novelty of the subject studied. The dietary modifications used in this study were very specific considering that only the concentration and the source of PCs of VOO administrated changed between interventions. The observed changes in the HDL proteome are smaller than those found when comparing a healthy population with patients with acute coronary syndrome [16] or with hemodialysis treatment [41]. However, our results are of great importance in the clinical practice meaning that small dietary changes can cause a remodeling of the HDL protein cargo improving the functionality of this particle.

## Conclusions

The results of our study illustrate the potential of HDL proteomics to lead to new biomarkers for CVD prevention. These HDL proteomics findings help to understand the improvement of the HDL functionality by measuring the effectiveness of nutritional interventions in a similar manner to pharmacological treatments as proposed by Birner-Gruenberger [40]. In conclusion, consumption of VOO, or phenol-enriched VOOs, has an impact on the HDL proteome in a cardioprotective mode that could enhance HDL functionality by up-regulating proteins related to cholesterol homeostasis, protection against oxidation, and blood coagulation while down-regulating proteins involved in acute-phase response, lipid transport, and immune response. The common protein expression modifications reported after the three VOOs indicate an important effect of the fatty acid and PC composition present in the common matrix of these VOO on the HDL remodeling. Further studies are needed, however, in order to assess the specific effects of each VOO incorporating different phenolic contents.

## Supporting Information

S1 Checklist CONSORT 2010 checklist. (DOC)

S1 Protocol TRIAL Protocol. (DOC)

S1 Fig Protein-protein interaction network for the 15 commonly differentially expressed proteins after the three VOO interventions (http:www.string-db.org). Proteins were presented with their gene encode symbol. Nodes represent genes encoding interacting proteins and the lines between them represent known and predicted interactions.
(TIFF)

S1 File Supplemental Methods. (DOCX)

S1 Table Concentration of the different phenolic metabolites present in the HDL pools. (DOCX)

S2 Table Virgin olive oils composition. Phenolic compounds, fat soluble micronutrients and fatty acids daily intake through 25 mL of VOO, FVOO and FVOOT.
(DOCX)

S3 Table Biochemical characterization of HDL segregated according to VOO intervention. (DOCX)

S4 Table HDL-associated proteins identified by MALDI and ORBITRAP MS techniques. (DOCX)

S5 Table Proteins differentially expressed after each VOO intervention. (DOCX)
