# Supplementary material for: Impact of Virgin Olive Oil and Phenol-Enriched Virgin Olive Oils on the HDL Proteome in Hypercholesterolemic Subjects: A Double Blind, Randomized, Controlled, Cross-Over Clinical Trial (VOHF Study)
Source: PLoS One. 2015 Jun 10;10(6):e0129160. doi: 10.1371/journal.pone.0129160 (PMC4465699; doi:10.1371/journal.pone.0129160)
Supplement: S1 Protocol — (DOC) [file pone.0129160.s002.doc]

**PROTOCOL S1**

**1. SUMMARY OF THE PROPOSAL**

**PROJECT TITLE:** VIRGIN OLIVE OIL AND HDL FUNCTIONALITY: A MODEL FOR TAILORING A FUNCTIONAL FOOD. The VOHF Project

**PRINCIPAL INVESTIGATOR: María Isabel Covas Planells**

| SUMMARY  (brief and precise, outlining only the most relevant topics and the proposed objectives): |
| --- |
| The global aim of the project is to assess whether functional olive oils, enriched both with their own phenolic compounds or with them plus additional complementary phenols from thyme, could act as nutraceuticals concerning the *in vivo* quantity and quality (functionality) of the human high density lipoproteins (HDL). This aim is expected to be attained in two steps. The first step (Tasks 1-4) comprises of the preparation of similar functional olive oils (enriched with their own phenolic content), but with differences in their total phenolic content (Task 1). Through a dose-response study the best ratio between quantity of phenolic compounds in olive oils/acute bioactive effects, assessed through the protective effect on lipid oxidative damage and endothelial dysfunction, will be assessed (Tasks 2-4). This will permit the selection of the best functional olive oil enriched with its own phenolic compounds. (Functional Olive Oil 1, FOO1) (Task 5). The second step (Tasks 6-8) is aimed at assessing whether the enrichment of the FOO1, by flavouring it with herbs with complementary structure/activity relationship (antioxidant mechanism) polyphenols such as thyme (rich in flavonols), (Functional Olive Oil 2, FOO2) could provide more benefits on human HDL amount and functionality than the FOO1. Other Tasks (9-11) include the centralized database management, statistical analyses, and dissemination plan. The obtained results could be immediately applied to functional olive oils and flavoured olive oil production, with a novel product having a greater added value, but withouth changes in the factories, thus without high increases in costs. We expect to obtain a nutraceutical olive oil(s) which could be potentially recommended as a second step in the guidelines of management of cardiovascular high-risk individuals. |

**2. INTRODUCTION**

(maximum **5** pages)

- The introduction should include: the aims of the project; the background and the state of the art of the scientific knowledge, including the essential references; the most relevant national and international groups working in the same or related topics.

**Introduction**

**Aims of the Project: The global aim of the project is to assess whether functional olive oils, enriched both with their own phenolic compounds or with them plus complementary phenols from thyme, could act as nutraceuticals concerning the *in vivo* quantity and quality (functionality) of the human high density lipoproteins (HDL).** To accomplish this aim we need: 1) to develop functional olive oils enriched with their own and other natural phenolic compounds by tailoring the quantity and quantity of total polyphenols for the best taste, bioavailability, and bioactivity (in *vivo* antioxidant and anti-endothelial dysfunction effects); and 2) to assess the effect of the phenolic compounds from the functional olive oils on the *in vivo* quantity and quality (functionality) of the human high density lipoproteins (HDL).

**Background:** Results of the **EUROLIVE Study** (The effect of olive oil on oxidative damage in European populations, (QLK1-CT-2001-00287). Coordinator: Dr. M.I. Covas, IMIM, Spain) study, a randomized, crossover study performed in 200 European individuals who participated in a nutritional intervention trial with 3 similar olive oils, but with differences in their phenolic content (1), showed an **increase of the HDL cholesterol levels and a decrease in the *in vivo* lipid oxidative damage in a dose-dependent manner with the phenolic content of the olive oil administered**. Within the frame of the **OLIVEOIL Project** (*Aceite de oliva enriquecido en compuestos fenólicos. Obtención de fenoles purificados, evaluación de su potencial antioxidante y desarrollo de un prototipo de aceite de oliva enriquecido*. AGL2005-07881-C02-01. Coordinator Dr. M.J. Motilva, Lleida University. IP of Subproject 2: Dr. Rosa Solá. Rovira and Virgili University) **a phenolic extract was obtained containing the main components of the virgin olive oil phenolic fraction** (oleuropein and ligtroside seicoiroids, flavonoids, phenolic acids, and lignans) **with a potent antioxidant activity** (ORAC value higher than 5000). **This extract permitted the development of a functional olive oil enriched with 1000 ppm of phenols** to be tested as an anti-hypertensive functional food. **To standardize olive oil phenolic compounds content is of relevance, as considerable variation exists in the quantity of antioxidant phenolics present in virgin olive oil. This may have ramifications for the bioactivity of the Mediterranean diet of which olive oil is an essential component.**

Besides the dose-dependent decrease of the lipid oxidative damage with the phenolic content of the olive oil, data from the EUROLIVE study (2) also showed that phenolic compounds from olive oil can modulate the postprandial oxidative stress after 40 mL olive oil ingestion. The degree of postprandial oxidative stress was inversely related with the phenolic content of natural similar olive oils from high (366 ppm) to low (2.7 ppm) phenolic content (2). Concerning virgin olive oils with high phenolic content, however, on one hand they have a bitter and pungent taste which could promote a refusal among consumers particularly those from non-Mediterranean areas. Also, the addition of bioactive ingredients to functional foods in high quantity could compromise the stability of the bioactive ingredients. On the other hand, **high phenolic compound-rich foods could have a dual action due to the fact that antioxidants can also act as oxidants**. The antioxidant system is a complex and delicate network of interacting molecules. When an antioxidant is oxidized it is converted into a harmful radical which needs to be reduced back to its reduced form by complementary antioxidants. The administration of high doses of a single type of antioxidants could even promote rather than reduce lipid peroxidation (3), and has been shown to increase atherosclerotic areas in animal models (4,5). In this sense, it has been reported that supplementing high-risk individuals with a single type of antioxidant (i.e. vitamin E) alone promoted rather than reduced lipid peroxidation (3), while the combination of vitamin E and vitamin C was effective in reducing atherosclerosis in human trials (6). There are three main biological antioxidant mechanisms for controlling lipid oxidation:1) free radical trapping and chain-breaking; 2)metal-chelating;3) and singlet oxygen quenching (7). **Hydroxytyrosol and the main phenols of the virgin olive oil, the secoiridoid group act, similarly to phenolic acids, inhibiting the lipid oxidation by trapping free and peroxy radicals. Other antioxidants, such as flavonoids, also help to control the extent of the lipid peroxidation by chelating metal (i.e. copper) ions ( 7).**

**Due to all the above, and on the basis of the Precautionary Principle adopted by the European Union (8), polyphenol-enriched food needs to be carefully monitored and tested in order to assess the best ratio between the degree of enrichment (concentration and type of antioxidants in the food) and bioavailability and bioactive effects.**

High density lipoprotein (HDL)-cholesterol levels are inversely and independently related with cardiovascular disease (9). Low levels of HDL cholesterol are the most characteristic lipid feature in individuals with premature coronary heart disease (10). **Currently, pharmacological or natural agents which can increase HDL cholesterol levels are considered to be the key factors for future therapies.** However, the recent unexpected association of torcetrapib, an agent that increases plasma HDL-C but with an increased cardiovascular mortality, has led not only to the discontinuation of further trials involving this drug (11), but also to considering the **functional quality of the HDL as being a more important issue than the circulating quantity of HDL**. The HDL can become a pro-inflammatory molecule through non-enzymatic (copper mediated oxidation; free radical attack…) and enzymatic mechanisms (i.e. via myeloperoxidase), thus reducing its functionality in the reverse cholesterol transport (12,13). Other than reverse cholesterol transport, however, HDL has several beneficial effects on arteries: experimental evidence suggests that HDL has anti-inflammatory, antioxidant and endothelial function enhancing properties on the arterial wall (14, 15). HDL mediates several mechanisms for improving endothelial function by signalling to endothelial nitric oxide (NO), up-regulating the activity of the synthase eNOS, and increasing NO bioavailability [[18]](http://www.sciencedirect.com/science?_ob=ArticleURL&_udi=B6T16-4V6YSV8-1&_user=1517318&_rdoc=1&_fmt=&_orig=search&_sort=d&view=c&_acct=C000053451&_version=1&_urlVersion=0&_userid=1517318&md5=c83f30de72de86a365da8a2b0e789952#bbib18) I.S. Yuhanna, Y. Zhu and B.E. Cox *et al.*, High-density lipoprotein binding to scavenger receptor-BI activates endothelial nitric oxide synthase, *Nat Med* **7** (2001), pp. 853–857. [**Full Text** via CrossRef](http://www.sciencedirect.com/science?_ob=RedirectURL&_method=outwardLink&_partnerName=3&_targetURL=http%3A%2F%2Fdx.doi.org%2F10.1038%2F89986&_acct=C000053451&_version=1&_userid=1517318&md5=0b7297bd9d93362068eb9e2a1cfebbad) | [View Record in Scopus](http://www.sciencedirect.com/science?_ob=RedirectURL&_method=outwardLink&_partnerName=655&_targetURL=http%3A%2F%2Fwww.scopus.com%2Fscopus%2Finward%2Frecord.url%3Feid%3D2-s2.0-0034927750%26partnerID%3D10%26rel%3DR3.0.0%26md5%3D46ce0fca449d56ae1a763b2a7685268b&_acct=C000053451&_version=1&_userid=1517318&md5=b5a67f5b2201304c4b636495072fdc5e) | [Cited By in Scopus (211)](http://www.sciencedirect.com/science?_ob=RedirectURL&_method=outwardLink&_partnerName=656&_targetURL=http%3A%2F%2Fwww.scopus.com%2Fscopus%2Finward%2Fcitedby.url%3Feid%3D2-s2.0-0034927750%26partnerID%3D10%26rel%3DR3.0.0%26md5%3D46ce0fca449d56ae1a763b2a7685268b&_acct=C000053451&_version=1&_userid=1517318&md5=f0ea79bee3fb988813e1b3497d8a2acc)(15)((16). Whether besides an increase in the amount of HDL cholesterol promoted by the phenolic content of the olive oil, as has been shown in the EUROLIVE study (1), an increase of the HDL functionality exists remains to be elucidated. The increase in HDL cholesterol in a direct relationship with the phenolic content of the olive oil has also been reported in animal models (17).The increase in HDL cholesterol associated with the consumption of polyphenol-rich food, other than olive oil, or supplements has also been reported in other human studies (18, 19).

Thus, **besides assessing mechanisms involved in the increase in HDL lipopoprotein from the intake of dietary phenolics, the effect of phenolic compounds on HDL functionality deserves to be investigated**. The physical-chemical modifications in the HDL which can deplete its functionality are linked with oxidative and nitroxidative stress, due to the presence of free ion metals or enzymatic and metabolic modifications such are those induced by hiperglycaemia, oxidation, and desialization (19). Thus, a first step, when the impact of an olive oil on HDL functionality is to be assessed, is to monitor its antioxidant “*in vivo*” capacity, as a tool for the predictive value of a functional olive oil to promote benefits on the HDL quantity and functionality. Increases in HDL cholesterol after the intake of a high phenolic content olive oil versus a low phenolic content one have been previously reported by our group (20, 21) and by Ramirez-Tortosa and co-workers (22) in Granada University, Spain. Reductions in oxidative damage and improvement in endothelial function with the phenolic content of the olive oil has been reported by the group of Pérez-Jimenez and co-workers in Cordoba University (23). The antioxidant capacity of functional olive oils enriched in tyrosol and hydroxytyrosol has been tested by the group of Dr. F. Visioli with the Milano University (Italy) team (24). Also, the group of Dr. Valentina Ruiz-Gutierrez in the Instituto de la Grasa (Spain) is developing olive oils enriched with the unsaponifiable fraction. However, and **to the best of our knowledge, no previous data or on-going work exist at European or international level concerning the effects of functional olive oils, enriched with its own and other polyphenols, as nutraceuticals for the increase in the amount and functionality of the HDL lipoprotein**.

The identification and validation of robust biomarkers for disease risk is a key factor for assessing the potential effectiveness and benefits of health-promoting food compounds. This is the basis for new and competitive economic and health developments in the food area as covered by the recently harmonized legislation on the emerging 'health claims (HC) made on food' in Europe (25). **The present project is aimed at capitalizing the milestones achieved in the previous EUROLIVE and OLIVEOIL Projects by tailoring a functional olive oil for its best suitability for promoting one of the current goals for modern cardiovascular therapies: the increase in the HDL functionality. The use of “omics” technology (genomics, proteomics, and metabolomics) in the project will assess the effect of functional olive oils at molecular level. The aim is expected to be attained in two steps.** **The first step (Tasks 1-4)** comprises the procedures for establishing the suitability and the best ratio between the quantities of polyphenols/acute bioactive effects, assessed through the protective effect on lipid oxidative damage and endothelial dysfunction, for functional olive oils enriched with their natural polyphenols (Functional Olive Oil 1, FOO1) (Task 5). **The second step (Tasks 6-8)** is aimed at assessing whether the enrichment of the FOO1 with polyphenols from another natural source such as thyme (rich in flavonols), with a complementary structure/activity relationship, could provide more benefits on the HDL functionality than the FOO1 (Functional Olive Oil 2, FOO2).Flavouring by spicing may result in an improvement of the stability of extra virgin olive oils (26) but in addition it is expected to further contribute to health benefits of olive oil. **Thyme is the herb selected for flavouring olive oil because is rich source of phenolic antioxidants** with metal-chelating properties, but their use must be carefully monitored by the characteristic flavour of some phenolic compounds (27). In this Project two types of flavouring methods (direct addition of the herbs or addition of extracts obtained by infusion) will be evaluated. To the best of our knowledge, this is the first time that flavoured olive oils are evaluated both for their richness in polyphenols and in human clinical trials for its healthy properties. Flavoured olive oils have increased upon the market in last years as an added value for the consumers’ due to their differential flavours. The obtained results could be immediately applied to functional and flavoured olive oil, with a novel product having a greater added value, but with minor costs in the production process. **Overall we expect to obtain a nutraceutical olive oil which could potentially to be recommended as a second step in the guidelines of management of cardiovascular high-risk individuals.**

**References**

1. Covas MI, Nyyssönen K, Poulsen HE, et.al. *The Effect of Polyphenols in Olive Oil on Heart Disease Risk Factors*. Ann Int Med 2006; 145: 333-341.
2. Covas MI, de la Torre K, Farré M, Kaikkonen J, et al. *Postprandial LDL phenolic content and LDL oxidation are modulated by olive oil phenolic compounds in human.* Free Radical Biol Med 2006;40:608-616.
3. Neutzil J, Thomas SR, Stocker R. Requirement for, promotion, or inhibition by alpha-tocopherol of radical-induced initiation of plasma lipoprotein lipid peroxidation. Free Rad Biol Med 1997;22:57-51.
4. Wilson T, Knight TJ, Beitz DC, Lewis DS and Engen RL, Resveratrol promotes atherosclerosis in hypercholesterolemic rabbits. *Life Sci* 59(1): PL15-21, 1996.
5. Acín S, Navarro MA, Arbonés-Manar JM, et al. Hydroxytyrosol administration enhances atherosclerotic lesion development in ApoE deficient mice.. J Biochem 2006; 140: 383-91.
6. Salonen JT, Nyyssonen K, Salonen R, et al. *Antioxidant supplementation in atherosclerosis prevention (ASAP) study: a randomized trial of the effect of vitamins E and C on 3-year progression of carotid atherosclerosis.* J Int Med 2000; 248: 377-86.
7. Scalbert A, Johnson IT, Saltmarsh M. *Polyphenols :antioxidants and beyond. Am* J Clin Nutr. 2005 ;81:215S-217S. Review.
8. European Union (2002), European Union consolidated versions of the treaty on European Union and of the treaty establishing the European community, Official Journal of the European Union, C325, [24 December](http://en.wikipedia.org/wiki/December_24) [2002](http://en.wikipedia.org/wiki/2002), Title XIX, article 174, paragraph 2 and 3.
9. Castelli WP, Doyle JT, Gordon T, et al. *HDL cholesterol and other lipids in coronary heart disease. The cooperative lipoprotein phenotyping study.* Circulation. 1977 May;55(5):767-72.
10. Schaefer EJ, Lamon-Fava S, Ordovas JM. *Factors associated with low and elevated plasma high density lipoprotein cholesterol and apolipoprotein A-I levels in the Framingham Offspring Study*. J Lipid Res. 1994;35:871-82.
11. Barter PJ, Caulfield M, Eriksson M, et al Effects of torcetrapib in patients at high risk for coronary events.. N Engl J Med. 2007;357:2109-22.
12. Ansell BJ, Watson KE, Fogelman AM, Navab M, Fonarow GC. High-density lipoprotein function recent advances.. J Am Coll Cardiol. 2005;46:1792-8.
13. Norata GD, Pirillo A, Catapano AL. *Modified HDL: biological and physiopathological consequences.* Nutr Metab Cardiovasc dis 2006,16:371-386.
14. Assmann A, Gotto M.*HDL cholesterol and protective factors in atherosclerosis*. Circulation 2004;109:8–14.
15. BarterPJ, Nicholls S, Rye, KA et al. *Antiinflammatory properties of HDL.* Circ Res 2004;95: 764–772.
16. Yuhanna IS, Zhu I,. Cox BE et al. *High-density lipoprotein binding to scavenger receptor-BI activates endothelial nitric oxide synthase*. Nat Med 2001;7:853–857.
17. Mangas-Cruz MA, Fernadez-Moyano A, Albi T, et al. Effects of minor constituents (non-glyceride compounds) of virgin olive oil on plasma lipid concentrations in male Wistar rats. Clin Nutr 2001;20:211-5.
18. Devaraj S, Vega-López S, Kaul N, Shönlau F, Rohdewald P, Jialal I. Supplementation with a pine bark extract rich in polyphenols increases plasma antioxidant capacity and alters the lipoprotein profile. Lipids. 2002;37:931-4.
19. Mursu J, Voutilainen S, Nurmi T, Rissanen TH, Virtanen JK, Kaikkonen J, et al. Dark chocolate consumption increases HDL cholesterol concentration and chocolate fatty acids may inhibit lipid peroxidation in healthy humans. Free Radic Biol Med. 2004;37:1351-9.
20. Marrugat J, Covas MI, Fitó M, et al. *Effects of differing phenolic content in dietary olive oils on lipids and LDL oxidation. A randomized controlled trial.* Eur J Nutr 2004;43:140-147.
21. Weinbrenner T, Fitó M, de la Torre R, et al.Olive oils high in phenolic compounds modulate oxidative/antioxidative status in men. J Nutr 2004;134:2314-21.
22. Ramírez-Tortosa C, López-Pedrosa JM. *Olive oil- and fish oil-enriched diets modify plasma lipids and susceptibility of LDL to oxidative modification in free-living male patients with peripheral vascular disease: the Spanish Nutrition Study*. Br J Nutr. 1999 82:31-9.
23. Ruano J, Lopez-Miranda J, Fuentes F, Moreno JA, Bellido C, Perez-Martinez P, Lozano A, Gómez P, Jiménez Y, Pérez Jiménez F. Phenolic content of virgin olive oil improves ischemic reactive hyperemia in hypercholesterolemic patients.J Am Coll Cardiol. 2005;46:1864-8.
24. Visioli F, Caruso D, Galli C, Viappiani S, Galli G, Sala A. *Olive oils rich in natural catecholic phenols decrease isoprostane excretion in humans.* Biochem Biophys Res Commun. 2000;278:797-9.
25. EU Regulation (EC) No 1924/2006 of the European Parliament and of the European Council of 20 December 2006 on nutrition and health claims made on foods, Official Journal of the European Union L 12 (2007) 3-18.
26. Gambacorta G, Faccia A, Pati S, Lamacchia C, Baiano A, La Notte E. Changes in the Chemicals and sensorial profile of extra virgin olive oils flavored with herbs and spices during storage. J Food Lipids 2007;14: 202-215.
27. Horwath el al (2008) Chemical characterisation of wild populations of Thymus from different climatic regions in southeast Spain Biochemical sistematics and ecology 36(2008) 117-133.

**3. OBJETIVES**

(maximum **2** pages)

- **3.1** Describe the reasons to present this proposal and the **initial hypothesis** which support its objectives (maximum **20** lanes)

| The present project is aimed at capitalizing the milestones achieved in the previous EUROLIVE and OLIVEOIL Projects by tailoring a functional olive oil for its best suitability for promoting one of the current goals for modern cardiovascular therapy: the increase in the HDL functionality. Results of the EUROLIVE (QLK1-CT-2001-00287) study, a randomized, crossover trial performed in 200 European individuals with 3 similar olive oil but with differences in their phenolic content, showed an increase of the HDL cholesterol levels and a decrease in the *in vivo* lipid oxidative damage in a dose-dependent manner with the phenolic content of the olive oil administered to healthy individuals. Within the frame of the OLIVEOIL Project (AGL2005-07881-C02-01) a functional olive oil with 1000 ppm of phenolic compounds has been developed from virgin olive oil enriched with its total phenolic extracts. The increase in HDL cholesterol associated with consumption of phenolic-rich food, other than olive oil, or supplements has been also reported in other human studies. For all above mentioned, we hypothesize that a functional olive oil tailored for providing the best relationship between phenolics (amount and type)/ phenolics bioavailability and bioactivity (antioxidant and anti-endothelial dysfunction) will be a useful tool for increasing not only the amount of HDL cholesterol, but also the functionality (antioxidant, anti-inflammatory, and reverse cholesterol transport capacity) of the human HDL *in vivo*. |
| --- |

- **3.2.** Indicate the **background and previous results** of your group or the results of other groups that support the initial hypothesis

| Data from an increase in HDL cholesterol after virgin olive oil consumption has been previously reported from some groups including those of Ramirez-Tortosa and co-workers in Granada University and Pérez-Jiménez and co-workers in Córdoba University, Spain. Previous results from the group of IMIM in humans, and from the group of Mangas-Cruz and co-workers in rats, showed an increase in HDL cholesterol with the phenolic content of the olive oil. However the full evidence of the dose-dependent increase of the HDL cholesterol related to the phenolic content of the olive oil was provided by the results of the EUROLIVE study, a large-sample size, and multicentre study in several European populations. The fact that in this study, and others, a decrease in the lipid oxidative damage was observed with the phenolic content of the olive oil support the hypothesis of the *in vivo* antioxidant capacity of olive oil as a protective factor for the HDL functionality. The improvement in stability and flavour of the extra virgin olive oil flavoured with herbs and spices reported (Gambacorta G and co-workers) support the hypothesis that a flavoured functional olive oil enriched not only with its own phenolics but also with complementary ones from thyme could provide additional health benefits and consumers’ acceptation. |
| --- |

- **3.3.** Describe briefly the **objectives** of the project.

| 1. **The main aim of the project is to assess whether functional olive oils, enriched both with their own phenolics or also with them plus complementary phenols from thyme, could act as nutraceuticals concerning the HDL lipoprotein functionality.**  Specific objectives are:  1.1 To prepare similar functional virgin olive oils with differences in their phenolic content (1000 ppm, 750 ppm, and 500 ppm) by optimizing the phenolic extraction procedure. To test their suitability and taste.  1.2 To prepare a functional olive oil enriched with its own phenolic compounds and those from thyme.  1.3 To assess if there is a threshold in the bioavailability of phenolic compounds from similar virgin olive oils enriched with the full spectrum of their phenolic compounds, but with differences in their total phenolic content.  1.4 To assess if there are interactions in the bioavailability of olive oil phenolic compounds with thyme components after the functional olive oil flavouring.  1.5 To assess which olive oil has the best dose/effect (*in vivo* antioxidant and anti-endothelial dysfunction) relationship from several olive oils enriched with the full spectrum of their own phenolic compounds (Functional Olive Oil 1, FOO1).  1.6 To assess the effect of the selected FOO1 on the human HDL amount and functionality *in vivo.*  1.7 To assess whether the flavouring of FOO1 with thyme (Functional olive oil 2, FOO2) provides additional benefits on the human HDL amount and functionality *in vivo*.  1.8 To assess the effect of the consumption of the functional olive oils on the phenolic content of the HDL.  1.9 To assess the relationship between the phenolic content of the HDL and its functionality.  1.10 To disseminate the results of the Project within the scientific community and the general public. |
| --- |

**3.4.** **For Coordinated projects** only, the **coordinator** must indicate (maximum **2** pages):

- the global objectives of the coordinated project, the need for coordination, and the added value provided by this coordination
- the specific objectives of each subproject
- the interaction among the objectives, activities and subprojects
- the mechanisms of coordination for an effective execution of the project.

The VOHF Project is a project which is based on a close relationship between Food Chemistry, Clinical, Human Pharmacology, and Biochemical Expertise for its development. Due to this, a multidisciplinary approach such as is provided by the different groups involved in the Project is required**.** Within each Subproject there are professionals with specific and complementary skills. The added value of the project is its translational nature which will permit the transfer from food technology innovations to possible health benefits and health claims for the developed products. Also, the project is aimed to provide insights on the mechanistic aspects by which a functional olive oil could exert health benefits.

Although each Subgroup has a major role in several tasks, the participation in several of them are intertwined. Specific objectives for each subproject would involve:

**Subproject 1.** Cardiovascular Risk and Nutrition Research Group. Instituto Municipal de Investigación Médica (IMIM-Hospital del Mar).Acronym: IMIM. PI: Dr. M.I. Covas. This Subproject is involved in Objectives: 1.3;1.4; 1.5;1.6;1.7; 1.8; 1.9; and 1.10

**Subproject 2.** Subproject 2. Lleida University. Acronym ULL IP. Dr. M.J. Motilva. This Subproject is involved in Objectives: 1.1; 1.2;1.3; 1.4; 1.5;1.6;1.7; 1.8; and 1.10

**.Subproject 3.** Subproject 2. Rovira and Virgili University. Acronym URV. IP. Dr. R. Solá.

This Subproject is involved in Objectives: 1.5; 1.6; 1.7;1.8; 1.9, and 1.10

The interaction among objectives, activities, and Subprojects is shown in Table 1.

For an effective management of the Project the Coordinator must carefully:

1. Check the accomplishment of the deadlines scheduled in the project for each Task and Subgroup.
2. Engage alternatives in the case that Point 1 would not be accomplished.
3. Establish common protocols for recording the general data of the participants and collection of biological samples.
4. Establish Specific Forms to be filled at the Sending and the Arrival of the biological samples.
5. Organize the Kick-off meeting and one meeting every nine months for Project Coordination.

**TABLE 1.INTERRELATIONSHIP BETWEEN OBJECTIVES, ACTIVITIES AND SUBPROJECTS**

| **TASK** | **Subproject** |  |
| --- | --- | --- |
| **Task 1.** **Functional virgin olive oils preparation** (**Phase I)**  ***OBJECTIVE: 1.1*** | **ULL** | From a virgin olive oil, and after an improvement of the phenolic compounds (PC) extraction procedure, similar olive oils but with a total PC of 1000, 750, and 500 ppm will be developed “de novo”. |
| **Task 2. Dose-Response Study**  ***OBJECTIVES: 1.3; 1.5*** | **URV** | Randomized, crossover, controlled trial;12 healthy volunteers; acute dose of 40 mL of 3 types of olive oils (1000, 750, and 500 ppm of PC). Biological samples will be collected at baseline and at postprandial times. |
| **Task 3. Bioavailability studies (Phase I)**  ***OBJECTIVE: 1.3.*** | **IMIM/ULL** | The bioavailability of phenolic compounds from olive oils will be assessed in samples of Task 2. |
| **Task 4. Bioactivity studies in the Dose-Response Study**  ***OBJECTIVE: 1.5*** | **URV** | Postprandial oxidative stress and endothelial dysfunction will be assessed in samples collected from participants in Task 2. |
| **Task 5. Selection of the best functional olive oil.**  ***OBJECTIVE: 1.5*** | **IMIM/ULL/**  **URV** | From data obtained in Tasks 1-4, the functional olive oil (FOO1) with the best phenol bioavailability/bioactivity will be selected. |
| **Task 6. Functional virgin olive oils preparation (Phase II).**  ***OBJECTIVE: 1.2*** | **ULL** | FOO1 will be enriched with thyme components, as a source of complementary antioxidants (FOO2). |
| **Task 7. Sustained Consumption Clinical Trial**  ***OBJECTIVES:***  ***1.6;1.7;1.8;1.9 and 1.4*** | **IMIM** | Randomized, crossover, controlled trial with 30 hyperlipemic individuals and 3 types of olive oil (refined (placebo), FOO1, and FOO2). Biological samples collected at baseline and before and after interventions. At the 1^st^ day a similar assay as reported in Task 2 (postprandial) will be performed with FOO1 and FOO2. |
| **Task 8. Bioactivity and HDL functionality in the Sustained Consumption Study**  ***OBJECTIVES:***  ***1.6;1.7;1.8; and 1.9*** | **IMIM/ULL/ URV** | The protective effect on oxidative damage and on the quantity and functionality of the HDL will be assessed at baseline and before and after each intervention in samples of Task 7. |
| **Task 9. Centralized Database management.**  ***All OBJECTIVES*** | **IMIM** | Individual data bases for each Subproject will be performed. A common data base for the study will be constructed on a standardized basis. |
| **Task 10. Statistical analyses**  ***All OBJECTIVES*** | **IMIM** | Statistical analyses performance |
| **Task 11. Dissemination of the results obtained**    ***OBJECTIVE: 1.10*** | **IMIM/ULL/ URV** | Scientific manuscripts and texts to be released to the media (TV, journals, C.Ass.) will be prepared for the scientific community and the general public. |

**4. METHODOLOGY AND WORKING PLAN**

**(in the case of coordinated projects this title must include all the subprojects)**

**Detail and justify precisely the methodology and the working plan**. Describe the working chronogram.

- The working plan should contain the tasks, milestones and deliverables. The projects carried out in the Hesperides or in the Antarctic Zone must include the operation plan.
- For each task, it must be indicated the Centre and the researchers involved in it.
- If personnel costs are requested, the tasks to be developed by the personnel to be hired must be detailed and justified. Remember that personnel costs are eligible only when personnel is contracted, **fellowships are not eligible** as personnel costs.

The working plan is based on the schedule and methods required to accomplish the Tasks reflected in Table 1.The interrelationship among Tasks is shown in Figure 1. An individualized description for each Task is provided.

Besides Coordinating the global Project, the role of Subproject 1 will be to lead Task 3 (Bioavailability Studies. Phase I), 7 (Clinical trial with sustained functional olive oil consumption), 9 (Bioactivity and HDL functionality in the sustained consumption study), 9 (Centralized Data base Management), 10 (Statistical Analyses), and 11 (Dissemination of the results). Also, Subproject 1 will perform as co-leader of Task 5 (Selection of the best functional virgin olive oil enriched with its own phenolic compounds) and will participate in Task 4 (Bioactivity in the Dose-Response Study).

The role of the Subproject 2 will be to lead Tasks 1 and 6 (Functional Olive Oils preparation I and II). Also, Subproject 2 will perform as co-leader of Task 5) Selection of the best functional virgin olive oil enriched with its own phenolic compounds) and will participate in Tasks 3 (Bioavailability Studies. Phase I) and 11 (Dissemination of the results).

The role of the Subproject 3 will be to lead Tasks 2 (Dose-Response Studies) and 4 (Bioactivity in the Dose-Response Study). Also, Subproject 3 will perform as co-leader of Task 5 (Selection of the best functional virgin olive oil enriched with its own phenolic compounds) and will participate in Tasks 8 (Bioactivity and HDL functionality in the sustained consumption study) and 11 (Dissemination of the results).

**Task 1**

Functional olive oil preparation (I)

**Task 2**

Dose-Response Study

**Task 3**

Bioavailability

Studies ( I)

**Task 4**

Bioactivity Studies in

Dose-Response Study

**Task 5**

Selection of the best

Functional olive oil

(FOO1)

**Task 6**

Functional olive oil preparation (II)

(FOO2)

**Task 7**

Sustained Consumption Study

**Task 8**

Bioactivity and HDL

Functionality in

Sustained Consumption Study

**Task 9.** CentralData

base management

**Task 10**. Statistical analyses

**Task 11.** Dissemination of the results

Figure 1. Interrelationship of Project Tasks

**TASKS DESCRIPTION**

| **Task 1** | **Starting date (Month):**  1 | | **Ending Date (Month):**  3 | | |
| --- | --- | --- | --- | --- | --- |
| **Task Title** | **Functional virgin olive oils preparation (Phase I)** | | | | |
| Activity type | RTD | | | | |
| Participants short name |  | ULL | |  |  |
| **Objectives**  1.1 To prepare similar functional olive oils, enriched with their own phenolic compounds , but with differences in their total phenolic content (1000 ppm, 750 ppm, and 500 ppm) | | | | | |
| **Description of work**  Task 1.1. Virgin olive oil phenolic compounds extraction: The main components of the virgin olive oil phenolic fraction, including the oleuropein and ligtroside derivatives (secoiridoid derivatives), phenolic acids, flavonoids and lignans will be extracted from olive oil cake (Suarez et al, 2009). On the basis of the expertise achieved in the OLIVEOIL project the selected method will be the accelerated solvent extraction (ASE) with improvements. A priori ethanol:water (80:20) will be considered as extraction solvent and the range of temperatures from 40 to 80 ºC. The main parameters for optimising will be: temperature, time extraction conditions, and proportion of the components of the extraction solvent. The resulting phenolic extracts will be rotary evaporated until full ethanol elimination and further freeze dried and stored at -80º C and N_2_ atmosphere. The optimal phenolic extract obtained will be used to prepare the enriched olive oils which will be used in Task 2 (Dose-response study).  Task 1.2. Preparation of functional olive oils enriched with their own phenolic compounds at concentrations of 1000ppm, 750 ppm, and 500 ppm: The phenolic extract from Task 1.1. will be added to a virgin olive oil with low phenolic content (below 100 ppm), as enrichment matrix. Different strategies for the addition of the extract will be evaluated (dispersion procedure by Polytron, ultrasonic treatments, and the screening of stabilizers to improve the dispersion and stability of the phenolic extract in the enrichment matrix.  Task 1.3. Monitoring the efficacy of olive oil enrichment. In the previous project (OLIVEOIL) the ULL group validated a method for the assessment of the phenolic compounds in olive oil by ultra-performance-liquid chromatography coupled to a tandem mass spectrometry (Suarez, 2008). This method will be applied to the individual quantification of phenols. The total (Folin-Cicalteau) phenolic content, the oxidative stability (Rancimat), and the antioxidant capacity (ORAC) will be evaluated. Two modalities of ORAC will be used: total ORAC assay (for evaluating the antioxidant activity of the whole enriched oil), and hydrophilic ORAC assay (for evaluating the antioxidant activity of hydrophilic part of the oil) (Artajo, 2006; Ortega, 2008).  Task 1.4. The possible consumers acceptance of the different types of functional olive oils will be determined by a Hedonic Test using a 9-point hedonic scale. | | | | | |
| **Deliverables**  D.1.1. Report on olive oil preparation and taste (Month 3) | | | | | |
| **Milestones**  M.1.2. Olive oils prepared for Task 2. (Month 3) | | | | | |

| **Task 2** | **Starting date (Month):**  1 | | **Ending Date (Month):**  6 | | |
| --- | --- | --- | --- | --- | --- |
| **Task Title** | **Dose –Response study** | | | | |
| Activity type | RTD | | | | |
| Participant short name |  |  | | URV |  |
| **Objectives**  2.1 To perform the dose- response study with functional olive oils from Task 1 | | | | | |
| **Description of work**  Task 2.1. 12 healthy volunteers (6 men and 6 women aged from 20 to 50) will be recruited. Physical examinations and routine biochemical laboratory determinations will be carried out to discard pathologies. Volunteers will be administered with each one of the following functional olive oils: FOO1000 pppm, FOO 750 ppm, and FOO500 ppm. The volume of olive oil to be administered at a single dose will be 40 ml. Olive oil administration will be assigned randomly. All subjects will be submitted to a wash-out period (WO) with one week of free-phenol diet between treatments. Participants will be requested to fulfill a dietary record during the 3 days prior to the intervention. The Center will provide dietary recommendations for the wash-out periods. Volunteers will be asked to not perform physical activity during the 3 days prior to the intervention. On the intervention day, blood will be collected at baseline and at 15 min, 30 min, 45 min, 1h, 2h, 4h and 6 h after olive oil administration. Urine will be collected in fractions of 2 hours for 10 hours.  This task will be performed at the URV.  **The physician will:**   1. Carry out anamnesis inquiry, physical examination, inclusion and exclusion criteria checked at the beginning of the study. 2. Be connected with the subjects if their laboratory values and other examination results are abnormal. 3. Supervise medical matters. 4.Check the non-laboratory data introduced in the database   **The nutritionist will:**  1. Give instruction on the adequate phenolic-free diet. 2. To convert the dietary records in food and nutrient groups to be entered in the Database of the Study.  **The nurse or qualified technician will:**   1. Draw blood samples 2.Measure the urine sample volume 2. Prepare aliquots of the specimens and freeze them following a standardized protocol   **The administrative or technical personnel will:**  Introduce the non-laboratory data in a specific bioavailability study database | | | | | |
| **Deliverables**  D.2.1. Database of the dose-response study with general characteristics (Month 6) | | | | | |
| **Milestones**  M.2.2. Dose-response study complexion (Month 6) | | | | | |

| **Task 3** | **Starting date (Month):**  5 | | **Ending Date (Month):**  15 | | |
| --- | --- | --- | --- | --- | --- |
| **Task Title** | **Bioavailability studies (Phase II)** | | | | |
| Activity type | RTD | | | | |
| Participants short name | IMIM | ULL | |  |  |
| **Objectives**  3.1. To assess the bioavailability of phenolic compounds from olive oils in samples of Task 2.  3.2. To assess whether a threshold exists for olive oil phenolic compound absorption from natural olive oils enriched with the full spectra of their phenolic compounds | | | | | |
| **Description of work**  Task 3.1**.** Tyrosol, hydroxytyrosol, and 0-methyl-hydroxytyrosol will be measured by GC/MS in plasma and urine following previously developed analytical methodology (Miro-Casas, 2003). Standards for glucuronide and sulfate conjugates will be obtained by biocatalyzed synthesis with a single-step product isolation and in high yield developed by the IMIM group (Khymenets,2006) (IMIM).  Task 3.2. Identification and quantification in human plasma of phenol metabolites by UPLC-ESI-MS/MS (ULL). The task will be focused on the detection of a broad spectra of phenolic fraction in biological fluids to evaluate their potential bioavailability (ULL) and their pharmacokinetics (IMIM). In a previous project (OLIVEOIL) the ULL group validated a method for the assessment of the phenolic compounds in olive oil (Romero, 2008). A validation started in the OLIVEOIL will be applied to plasma and urine for measuring a broad spectra of olive oil phenolics (including oleuropein and ligstroside aglicones, lignans, flavonoids, and conjugates). The procedure includes sample preparation using off-line SPE (OASIS HLB, Waters Corp., Milford, MA) and UPLC analysis of phenols using a Waters Acquity Ultra-Performance^TM^ LC with binary pump.  Plasma in Tasks 3.1 and 3.2 will be analyzed at baseline at 15 min, 30 min, 45 min, 1h, 2h, 4h and 6 h after olive oil ingestion and in urine every 2 hours until 12 hours after olive oils administration (IMIM- ULL). Total number of samples= 396  Task 3.3. Comparative study of the quantification of tyrosol, hydroxytyrosol, o-methyl-hydroxytyrosol, and their glucuronides and sulfates in human plasma by GC/MS and UPLC-ESI-MS/MS. The project offers a good opportunity to perform an interlaboratory assay (IMIM and ULL) using common plasma samples (Task 2). This comparative analysis will further strengthen bioavailability results and will permit the evaluation of the performance achieved using the UPLC-ESI-MS/MS method. | | | | | |
| **Deliverables**  D.3.1. Data base of the dose-response study with data of phenolic compounds in plasma and urine (Month 15)  D.3.2. Comparison of two procedures (GC/MS and UPLC-ESI-MS/MS) for tyrosol, hydroxytyrosol and their biological metabolites in plasma (Month 15). | | | | | |
| **Milestones**  M.3.1. Data base of the dose-response study with data of phenolic compounds in plasma and urine (Month 15) | | | | | |

| **Task 4** | **Starting date (Month):**  5 | | **Ending Date (Month):**  15 | | |
| --- | --- | --- | --- | --- | --- |
| **Task Title** | **Bioactivity studies in the Dose-Response Study** | | | | |
| Activity type | RTD | | | | |
| Participants short name | IMIM |  | | URV |  |
| **Objectives**  4.1. To assess the in vivo bioactivity of the functional olive oils prepared in Task 1 in samples of Task 2 | | | | | |
| **Description of work**  Task 4.1. Oxidative markers will be measured at baseline and at 2h, 4h, and 6 h (IMIM) after olive oil administration.: Plasma oxidized LDL and urinary metabolites of the 3’- chloro-tyrosine will be measured by ELISA and GC/MS (IMIM) (Covas MI, 2006a, b).  Protein carbonyl content (PCC) will be measured by spectrometry, 8-isoprostane (8-epi PGF2α), and 8-hydroxydeoxyguanosine (8-OHdG) by enzyme-linked immunosorbent assay (ELISA) (URV).Total number of samples= 144.  Task 4.2 Plasma glucose and lipid profile will be measured by enzymatic methods in automated mode at baseline and at 2h, 4h, and 6 h (URV). Total number of samples= 144.  Task 4.3 Endothelial function will be measured through the assessment of ischemic reactive hyperemia (IRH). Ischemic reactive hyperemia (IRH) will be measured with a Laser-Doppler procedure (linear Periflux 5000 (Perimed S.A., Stockholm, Sweden) at baseline and at 4 h and 6 after olive oil intake (Ruano, 2005). | | | | | |
| **Deliverables**  D.4.1. Database of the dose-response study with data of bioactivity parameters (Month 15) | | | | | |
| **Milestones**  M.4.1. Database of the dose-response study with data of bioactivity parameters (Month 15) | | | | | |

| **Task 5** | **Starting date (Month):**  16 | | **Ending Date (Month):**  17 | | |
| --- | --- | --- | --- | --- | --- |
| **Task Title** | **Selection of the best functional olive oil (FOO1).** | | | | |
| Activity type | RTD | | | | |
| Participant short name | IMIM | ULL | | URV |  |
| **Objectives**  5.1 To select the best virgin olive oil enriched with its phenolic content (FOO1) | | | | | |
| **Description of work**  Task 5.1 On the basis of the results obtained in Tasks 2-4 and data from the statistical analyses performed in Task 10 the functional olive oil (FOO1) with the best phenolics bioavailability and bioactivity will be selected. | | | | | |
| **Deliverables**  5.1. Report on the rationale for the olive oil selection (Month 17) | | | | | |
| **Milestones**  M.5.1 Selection of the FOO1 for Tasks 6 and 7 (Month 17) | | | | | |

| **Task 6** | **Starting date (Month):**  4 | | **Ending Date (Month):**  18 | | |
| --- | --- | --- | --- | --- | --- |
| **Task Title** | **Functional virgin olive oils preparation (Phase II)** | | | | |
| Activity type | RTD | | | | |
| Participants short name |  | ULL | |  |  |
| **Objectives**   - 1. To prepare a functional olive oil enriched not only with its own phenolic compounds but with complementary phenols from thyme (FOO2). | | | | | |
| **Description of work**  Task 6.1. Monitoring the best procedure for flavoring the FOO1 with thyme components: Two procedures of flavouring methods will be evaluated: 1) infusion by the direct addition of the herbs to the functional virgin olive oils or 2) addition of phenolic extract from thyme previously obtained by ASE system (similar way as Task 1.2). In both methods the optimal conditions to reach the maximal phenolic transference from the herb to the olive oil will be selected. The selection of the optimal proportion of olive oil phenols/thyme phenols will be based on the maximal antioxidant capacity by ORAC assay. The optimal phenolic extract obtained from thyme will be used to prepare different mixtures with phenolic olive paste extract. The monitorization of the phenol enrichment will be based on the concentration of the more important flavonoids of the thyme with a molecular structure that favors the antioxidant mechanism by chelating metal: luteolin (the major flavone), eriodyctiol and naringenin (the major flavanones), and taxifolin (the major dihydroflavonol). The total (Folin-Cicalteau) and individual (ultra-performance-liquid chromatography coupled to a tandem mass spectrometry with a binary pump system) phenolic content, oxidative stability (Rancimat), and antioxidant capacity (ORAC, both total and hydrophilic, (Artajo, 2006; Ortega, 2008) will be evaluated. The consumer acceptance of the different types of flavored olive oils will be determined by a Hedonic Test using a 9-point hedonic scale, considering the extremes as “dislike extremely” and “like extremely”, respectively.  Task 6.2. FOO2 Preparation: The olive oil selected from Task 5, the functional olive oil (FOO1), will be prepared at the same dose of phenolic compounds, but with the addition of a complementary source of natural flavonoids from thyme (FOO2). The optimal polyphenol mixture and procedure will be selected from the results of Task 6.1 | | | | | |
| **Deliverables**  D.6.1**.** Report of the best procedure to enrich a functional olive oil with thyme components (Month 16)  D.6.2. Report on the characteristics of the FOO2 (Month 18). | | | | | |
| **Milestones**  M.6.1 Completion of FOO2 for Task 7(Month 18) | | | | | |

| **Task 7** | **Starting date (Month):**  15 | | **Ending Date (Month):**  24 | | |
| --- | --- | --- | --- | --- | --- |
| **Task Title** | **Sustained Consumption Clinical Trial** | | | | |
| Activity type | RTD | | | | |
| Participants short name | IMIM |  | |  |  |
| **Objectives**  7.1 To develop a clinical supplementation trial with functional olive oils and to obtain biological samples and participants’ general characteristics, physical activity, and diet | | | | | |
| **Description of work**  Task 7.1 Cross-over clinical supplementation trial, in which 30 subjects (15 men and 15 women) will be randomised to one of 3 orders of administration of 25 mL/day of raw refined virgin olive oil, FOO1 (functional olive oil enriched with its own phenolic compounds), and FOO2 (functional olive oil enriched with its olive oil phenolic compounds plus those of thyme), in 3 periods of 3 weeks (previous intervention). Current intervention is a randomized, controlled, double-blind, corss-over clinical supplementation trial, in which 33 subjects (19 men and 14 women) will be randomised to one of 3 orders of administration of 25 mL/day of raw refined virgin olive oil, FOO1, and FOO2, in 3 periods of 3 weeks. Prior to the interventions, there was a 2-week wash-out period where common olive oil was the main fat consumed. To avoid an excessive intake of antioxidants, such as PC, during the clinical trial period, the participants were advised to limit their consumption of polyphenol-rich foods. A 3-day dietary record, which records the type, quantity, and preparation of food consumed, was administered to participants during the last three days before baseline and before and after each intervention period to control their diet throughout the studyDemographic data, alcohol and drug consumption will be recorded. Physical examination, blood pressure and anthropometric measurements will be performed. The previous eligibility criteria were the following: *inclusion criteria:* Participants will be hyperlipemic patients, with low HDL cholesterol, aged 20-60 years. *Exclusion criteria*: 1) Intake of antioxidant supplement or acetylsalicylic acid or any other drug with established antioxidative properties. 2) Athletes with PA >3000 kcal/week in leisure-time. 3) Obesity (BMI > 30 kg/m^2^). 4) Diabetes, multiple allergies, intestinal diseases.5) Any condition that limits the mobility of the subject making study visits impossible.6) Life threatening illness such as cancer or severe disease with a lowered expected 3-year survival.7) Any other disease or condition that would worsen the adherence to the measurements or treatment. The current eligibility criteria are the following: *inclusion criteria*: hypercholesterolemic volunteers (total cholesterol > 200 mg/dL), aged 35 to 80. *Exclusion criteria:* 1) BMI > 35 kg/m2. 2) Smokers. 3) Athletes with high physical activity (>3000 kcal/day). 4) diabetes, multiple allergies, intestinal diseases. 5) any condition that limits the mobility of the subject making study visits impossible. 6) Life threatening illness such as cancer or severe disease with a lowered expected 3 year survival. 7) Any other disease or condition that would worsen the adherence to the measurements or treatment. *Sample size calculation:* The sample size of 30 individuals allows at least 80% power to detect a statistically significant difference among groups of 3 mg/dL of HDL cholesterol, assuming a drop out rate of 15% and a Type I error of 0.05 (2-sided).  **The physician will:** 1) Carry out anamnesis inquiry, physical examination, inclusion and exclusion criteria at the beginning the study; 2) Be connected with the subjects if their laboratory values and other examination results are abnormal; 3) Supervise medical matters; and 4)Check the non-laboratory data introduced in the database  **The nutritionist will:** Give instruction on the adequate phenolic-free diet. To convert the dietary records in food and nutrient groups to be entered in the Database of the Study.  **The nurse or qualified technician will:** 1) Draw blood samples; 2) Measure the urine samples volume; 3) Prepare aliquots of the specimens and freeze them following a standardized protocol; 4)Carry out anthropometric and blood pressure measurements;5)Administration of physical activity practice questionnaire (Elosua, 2000).  **The administrative personnell will:** Introduce the non-laboratory data in a database | | | | | |
| **Deliverables**  D.7.1. Database of the sustained consumption clinical trial with general characteristics (Month 24) | | | | | |
| **Milestones**  M.7.1 Completion of the sustained consumption clinical trial (Month 24) | | | | | |

| **Task 8** | **Starting date (Month):**  20 | | **Ending Date (Month):**  33 | | |
| --- | --- | --- | --- | --- | --- |
| **Task Title** | **HDL lipoprotein functionality and antioxidant capacity assessment in the Sustained Consumption study** | | | | |
| Activity type | RTD | | | | |
| Participant short name | IMIM |  | | URV |  |
| **Objectives**  8.1 To assess the HDL amount and functionality after FOO1 and FOO2 consumption.  8.2 To assess changes in the in vivo protective effect on oxidative damage of the above mentioned functional olive oils.  8.3 To assess whether nutrigenomic changes occur in genes related with HDL quantity  and functionality | | | | | |
| **Description of work**  At baseline and before and after each intervention in Task 7.1 , the following parameters will be measured (Total sample size=210 samples):  Task 8.1 Markers of compliance: Measurement of tyrosol and hydroxytyrosol in spot morning urine will be performed by GC/mass spectrometry (IMIM) (Covas, 2006a).  Task 8.2 Oxidative damage. Plasma lipids will be performed by enzymatic methods in a automated mode. In vivo circulating oxidized LDL and 3’ chlorotyrosine derivates in urine will be determined by ELISA (IMIM) (Covas, 2006 a and b).  Protein carbonyl content (PCC) by spectrometry, 8-isoprostane (8-epi PGF2α), and 8-hydroxydeoxyguanosine (8-OHdG) by enzyme-linked immunosorbent assay (ELISA) (URV).  Endothelial function and Intestinal microorganism study.  Task 8.3. Parameters of HDL functionality: Isolation of HDL will be done by sequential centrifugation. The following parameters will be measured in isolated HDL: 1)Fatty acid content, vitamin E, and total phenolic content in HDL by HPLC-DAD (Gimeno , 2007); 2)Apolipoproteins (Apo)A1, ApoA2, and ApoA4 by ELISA; Cholesterol esther transfer protein (CETP) and lecitine cholesterol acyl transferase (LCAT) by fluorometry. Paraoxonase (Organophosphatase activity) PON1) and PAF-AH activities by spectrometry with automated mode. 3’ chlorotyrosine and 3’-nitro-tyrosine by GC/MS (IMIM) (Covas, 2006a).  Bilayer fluidity of HDL will be measured by confocal microscopy. Cholesterol efflux from cells will be measured in culture of macrophages (Girona, 2003) (URV).  Task 8.4 OMICS. Gene expression assay will be performed as previously described (Khymenets 2008 and 2009). Several candidate genes implicated in the increase of the HDL cholesterol and in the improvement of the HDL functionality in humans will be tested. Candidate genes: ABCA1: ATP-binding cassette, sub-family A (ABC1), member 1; ABCG1: ATP-binding cassette, sub-family G (WHITE), member 1; and ABCG4: ATP-binding cassette, sub-family G (WHITE), member 4 ApoA1: apolipoprotein A-I; and ApoE: apolipoprotein E; LPL: lipoprotein lipase; PAF-AH1B3: platelet-activating factor acetylhydrolase, isoform Ib, gamma subunit 29kDa; PPARalpha: peroxisome proliferator-activated receptor alpha; PPARgamma: peroxisome proliferator-activated receptor gamma; and PBPPAR: PPAR binding protein; RARA: retinoic acid receptor, alpha. Proteomic analyses, by separation with 2-DE (two-dimensional gel electrophoresis) and PMF (protein mass fingerprinting) with MALDI-TOF-MS (matrix-assisted laser desorption/ionization time of flight mass spectrometry ,will be performed in HDL samples (Heinecke, 2008). Metabolomics will be performed by UPLC–MS/MSin urine samples (Bijlsma, 2006). | | | | | |
| **Deliverables**  D.8.1. Data base of the sustained consumption study with compliance, oxidative damage, and HDL functionality markers (Month 33) | | | | | |
| **Milestones**  M.8.1 completion of the laboratory determinations in the sustained consumption clinical trial (Month 33) | | | | | |

| **Task 9** | **Starting date (Month):**  1 | | **Ending Date (Month):**  33 | | |
| --- | --- | --- | --- | --- | --- |
| **Task Title** | **Centralized database management** | | | | |
| Activity type | RTD | | | | |
| Participant short name | IMIM |  | |  |  |
| **Objectives**  9.1 To distribute individual databases for each Subgroup with the corresponding parameters to be introduced.  9.2. To integrate all Databases with all data in the Common Database of the VOHF Study. | | | | | |
| **Description of work**  Task 9.1.To construct individual databases in ACCES to be distributed for each Subproject.  Task 9.2.To construct Common Data Bases including all field centre and laboratory database using the same variable names and codes.  Task 9.3. To code the open ended questions to assure criteria homogeneity.  Task 9.4. To identify and correct miscoded data through cross-tabulations of raw data and consultations with research partners.  Task 9.5. To examine missing data and apply the proper handling of missing data and imputation procedures.  Task 9.6. To calculate scores or scales and indexes using standard techniques.  Task 9.7. To develop the analysis manual (including variable names, range, algorithm of score calculation).  All Tasks will be performed at the IMIM | | | | | |
| **Deliverables**  D.9.1. Individual databases (Month 4)  D.9.2. Common Complete Edited Database for the Dose-Response Trial (Month 17).  D.9.3. Common Complete Database for the Bioavailability Study nested in the Sustained Consumption Trial (Month 32).  D. 9.4. Common Complete DataBase for the Sustained Consumption Clinical Trial (Month 33). | | | | | |
| **Milestones**  M.9.1. Set up of individual databases (Month 4)  M.9.2. Completion of Database for the Dose- Response Trial (Month 17)  M.9.3. Completion of Database for the Bioavailability Study nested in the Sustained Consumption Trial (Month 32).  M9.4. Completion of Database for the Sustained Consumption Clinical Trial (Month 33) | | | | | |

| **Task 10** | **Starting date (Month):**  16 | | **Ending Date (Month):**  34 | | |
| --- | --- | --- | --- | --- | --- |
| **Task Title** | **Statistical analyses** | | | | |
| Activity type | RTD | | | | |
| Participant short name | IMIM |  | |  |  |
| **Objectives**  10.1 To perform the statistical analyses for analyzing the data obtained in the Project. | | | | | |
| **Description of work**  Task 10.1. To perform all pooled analyses under the direction of the Coordinator and various collaborating investigators for data from the Dose-Response Study.  Task 10.2. To perform all pooled analyses under the direction of the Coordinator and various collaborating investigators for data from the Bioavailability Study nested in the Sustained Consumption Trial.  Task 10.3. To perform all pooled analyses under the direction of the Coordinator and various collaborating investigators for data from the Sustained Consumption Clinical Trial.  All Tasks will be performed at the IMIM | | | | | |
| **Deliverables**  D.10.1. Set of statistical analyses for analyzing the phenolic compound bioavailability and the bioactive parameters in the Dose-Response Study (Month 18).  D.10.2. Set of statistical analyses for analyzing the phenolic compound bioavailability in the Bioavailability Study nested in the Sustained Consumption Trial (Month 32).  D.10.3. Set of statistical analyses for analyzing the bioactivity parameters in the Sustained Consumption Trial (Month 34). | | | | | |
| **Milestones**  M.10.1. Completion of Statistical analyses of the Dose-Response Study(Month 18).  M.10.2. Completion of Statistical analyses of the Bioavailability Study nested in the Sustained Consumption Trial (Month 32).  M.10.3. Completion of Statistical analyses of the Sustained Consumption Trial (Month 34). | | | | | |

| **Task 11** | **Starting date (Month):**  20 | | **Ending Date (Month):**  36 | | |
| --- | --- | --- | --- | --- | --- |
| **Task Title** | **Dissemination of the results** | | | | |
| Activity type | DT | | | | |
| Participants short name | IMIM | ULL | | URV |  |
| **Objectives**  11.1 To prepare scientific manuscripts for their publication in scientific journals  11.2 To prepare a manuscript and a leaflet to be disseminated in the media for the general public. | | | | | |
| **Description of work**  Task 11.1 .Scientific manuscripts will be prepared concerning: 1) The effects of the consumption of similar functional olive oils but with differences in their phenolic content (leader URV); 2) The bioavailability of olive oil phenolic compounds from functional olive oils enriched or not with complementary phenols from thyme (leader ULL); 3) Scientific manuscript on the comparison of the performance of two procedures (GC/MS and UPLC-ESI-MS/MS) for tyrosol, hydroxytyrosol and their biological metabolites detection and quantification in human plasma (Leader IMIM); 4)The effect of functional olive oils on lipid oxidative damage and HDL functionality (leader IMIM).  Task 11.2. A manuscript addressed to consumers with the results of the project will be performed to be sent to European and International Consumer Associations. A leaflet explaining the results of the project and the cost/benefit ratio of each type of olive oil to be sent to the media will be prepared (leader IMIM) | | | | | |
| **Deliverables**  D.11.1. Scientific manuscript on the effects of the consumption of similar functional olive oils but with differences in their phenolic content (Month 22).  D. 11.2 Scientific manuscript on the comparison of the performance of two procedures (GC/MS and UPLC-ESI-MS/MS) for tyrosol, hydroxytyrosol and their biological metabolites detection and quantification in human plasma (Month 22).  D11.3. Scientific manuscript on the bioavailability of olive oil phenolic compounds from functional olive oils enriched or not with complementary phenols from thyme (Month 34).  D.11.4 Scientific manuscript on the effect of functional olive oils on lipid oxidative damage and HDL functionality (Month 36).  D.11.5 Dissemination manuscript for European Consumer Associations (Month 36)  D.11.6. Explanatory leaflet of the Project for the media (Month 36). | | | | | |
| **Milestones**  M.11.1. Completion of the scientific manuscript on the effects of the consumption of similar functional olive oils but with differences in their phenolic content (Month 22).  M.11.2 Completion of the scientific manuscript concerning two procedures for tyrosol, hydroxytyrosol and their biological metabolites detection and quantification of in human plasma (Month 22) .  M.11.2. Completion of the scientific manuscript on the bioavailability of olive oil phenolic compounds from functional olive oils enriched or not with complementary phenols from thyme (Month 34)  M.11.3. Completion of the scientific manuscript on the effect of functional olive oils on lipid oxidative damage and HDL functionality (Month 36).  M.11.4 Completion of the Divulgative manuscript for general public and leaflet for the media (TV, journals, radio..) (Month 36). | | | | | |

**REFERENCES FOR METHODS**

Artajo, L.S.; Romero, M.P.; Morelló, J.R.; Motilva, M.J. Enrichment of refined olive oil with phenolic compounds: Evaluation of their antioxidant activity and their effect on the bitter index. J Agricultural and Food Chemistry 2006; 54 (16): 6079-88.

-[Bijlsma S](http://www.ncbi.nlm.nih.gov/sites/entrez?Db=pubmed&Cmd=Search&Term="Bijlsma S"%5BAuthor%5D&itool=EntrezSystem2.PEntrez.Pubmed.Pubmed_ResultsPanel.Pubmed_DiscoveryPanel.Pubmed_RVAbstractPlus), [Bobeldijk I](http://www.ncbi.nlm.nih.gov/sites/entrez?Db=pubmed&Cmd=Search&Term="Bobeldijk I"%5BAuthor%5D&itool=EntrezSystem2.PEntrez.Pubmed.Pubmed_ResultsPanel.Pubmed_DiscoveryPanel.Pubmed_RVAbstractPlus), [Verheij ER](http://www.ncbi.nlm.nih.gov/sites/entrez?Db=pubmed&Cmd=Search&Term="Verheij ER"%5BAuthor%5D&itool=EntrezSystem2.PEntrez.Pubmed.Pubmed_ResultsPanel.Pubmed_DiscoveryPanel.Pubmed_RVAbstractPlus), [Ramaker R](http://www.ncbi.nlm.nih.gov/sites/entrez?Db=pubmed&Cmd=Search&Term="Ramaker R"%5BAuthor%5D&itool=EntrezSystem2.PEntrez.Pubmed.Pubmed_ResultsPanel.Pubmed_DiscoveryPanel.Pubmed_RVAbstractPlus), [Kochhar S](http://www.ncbi.nlm.nih.gov/sites/entrez?Db=pubmed&Cmd=Search&Term="Kochhar S"%5BAuthor%5D&itool=EntrezSystem2.PEntrez.Pubmed.Pubmed_ResultsPanel.Pubmed_DiscoveryPanel.Pubmed_RVAbstractPlus), [Macdonald IA](http://www.ncbi.nlm.nih.gov/sites/entrez?Db=pubmed&Cmd=Search&Term="Macdonald IA"%5BAuthor%5D&itool=EntrezSystem2.PEntrez.Pubmed.Pubmed_ResultsPanel.Pubmed_DiscoveryPanel.Pubmed_RVAbstractPlus), [van Ommen B](http://www.ncbi.nlm.nih.gov/sites/entrez?Db=pubmed&Cmd=Search&Term="van Ommen B"%5BAuthor%5D&itool=EntrezSystem2.PEntrez.Pubmed.Pubmed_ResultsPanel.Pubmed_DiscoveryPanel.Pubmed_RVAbstractPlus), [Smilde AK](http://www.ncbi.nlm.nih.gov/sites/entrez?Db=pubmed&Cmd=Search&Term="Smilde AK"%5BAuthor%5D&itool=EntrezSystem2.PEntrez.Pubmed.Pubmed_ResultsPanel.Pubmed_DiscoveryPanel.Pubmed_RVAbstractPlus).Large-scale human metabolomics studies: a strategy for data (pre-) processing and validation. Anal. Chem. 2006; 78: 567-74.

-Covas MI, de la Torre K, Farré M, Kaikkonen J, Fitó M, López-Sabater C, Pujadas-Bastardes M, Juglar J, Weinnbrenner T, Lamuela-Raventós R, de la Torre R. Postprandial LDL phenolic content and LDL oxidation are modulated by olive oil phenolic compounds in humans. Free Rad Biol Med 2006;40:608-616.

-Covas MI, Nyyssönen K, Poulsen HE, Kaikkonen J, Zunft HJF,Kiesewetter H, Gaddi A, de la Torre R, Mursu J, Bäumler H, Nascetti S, Salonen, JT, Fitó M, Virtanen J, Marrugat J. The Effect of Polyphenols in Olive Oil on Heart Disease Risk Factors. Ann Int Med 2006(a); 145: 333-341.

-Elosua R, García M, Aguilar A, Molina L, Covas MI, Marrugat J on behalf of investigators of the MARATDON Group. *Validation of the Minnesota Leisure Time Physical Activity questionnaire in Spanish women.* Med & Science Sports Exercise 2000; 32: 1431-37.

-Gimeno E, de la Torre-Carbot K, Lamuela-Raventós RM, Castellote AI, Fitó M, de la Torre R, Covas MI, López-Sabater MC. *Changes in the phenolic content of low density lipoprotein after olive oil consumption in men. A randomized crossover controlled trial.* Br J Nutr 2007 ; 98, 1243–1250.

-[Girona J](http://www.ncbi.nlm.nih.gov/sites/entrez?Db=pubmed&Cmd=Search&Term="Girona J"%5BAuthor%5D&itool=EntrezSystem2.PEntrez.Pubmed.Pubmed_ResultsPanel.Pubmed_DiscoveryPanel.Pubmed_RVAbstractPlus), [LaVille AE](http://www.ncbi.nlm.nih.gov/sites/entrez?Db=pubmed&Cmd=Search&Term="LaVille AE"%5BAuthor%5D&itool=EntrezSystem2.PEntrez.Pubmed.Pubmed_ResultsPanel.Pubmed_DiscoveryPanel.Pubmed_RVAbstractPlus), [Solà R](http://www.ncbi.nlm.nih.gov/sites/entrez?Db=pubmed&Cmd=Search&Term="Solà R"%5BAuthor%5D&itool=EntrezSystem2.PEntrez.Pubmed.Pubmed_ResultsPanel.Pubmed_DiscoveryPanel.Pubmed_RVAbstractPlus), [Motta C](http://www.ncbi.nlm.nih.gov/sites/entrez?Db=pubmed&Cmd=Search&Term="Motta C"%5BAuthor%5D&itool=EntrezSystem2.PEntrez.Pubmed.Pubmed_ResultsPanel.Pubmed_DiscoveryPanel.Pubmed_RVAbstractPlus), [Masana L](http://www.ncbi.nlm.nih.gov/sites/entrez?Db=pubmed&Cmd=Search&Term="Masana L"%5BAuthor%5D&itool=EntrezSystem2.PEntrez.Pubmed.Pubmed_ResultsPanel.Pubmed_DiscoveryPanel.Pubmed_RVAbstractPlus). HDL derived from the different phases of conjugated diene formation reduces membrane fluidity and contributes to a decrease in free cholesterol efflux from human THP-1 macrophages. [Biochim Biophys Acta.](javascript:AL_get(this, 'jour', 'Biochim Biophys Acta.');) 2003 22;1633(3):143-8.

-Heinecke J. The HDL Proteome: A Marker—and Perhaps Mediator—of Coronary Artery Disease. J Lipid Res. 2008 PMID: 19060251.

-Khymenets O, Joglar J, Clapés P, Parella T, Covas MI, de la Torre R. Bioacatalyzed synthesis and structural characterization of monoglucuronides of hydroxytyrosol, tyrosol, homovanillic alcohol, and 3-(4’-hydroxyphenyl) propanol. Adv. Synth. Catal 2006; 348:2155-62.

- Khymenets O, Covas MI,Farré M, Langohr K, Fitó M, de la Torre R. Role of sex and time of blood sampling in SOD1 and SOD2 expression variability. Clin Biochem 2008;41:1348–54.

-Khymenets O, Fitó M, Covas M I, Farré M, Pujadas M A, Muñoz D, Konstantinidou V, de la Torre R. Mononuclear cell transcriptome response after sustained virgin olive oil consumption in humans: an exploratory nutrigenomics study. OMICs 2009 (in press).

-Miró-Casas E, Covas MI, Farré M, Fitó M, Ortuño J, Weinbrenner T, Roset P, de la Torre R. Hydroxytyrosol disposition in humans. Clin Chem. 2003;49:945-52.

-Ortega N; Romero MP; Macià A; Reguant J; Anglès N; Morelló JR; Motilva MJ. Obtention and characterization of phenolic extracts from different cocoa sources. J Agricultural and Food Chemistry 2008; 56 (20): 9621-27.

-Romero MP, Casanovas M, Valls RM, Solà R, Girona J, Heras M, Gónzalez C, Soler A, Motilva MJ. Study of the phenolic profile in human plasma after the consumption of virgin olive oil. XXIV International Conference on Polyphenols. Polyphenols Communications, 2008. Volume I. pp. 779-780 (manuscript in preparation).

-Ruano J, Lopez-Miranda J, Fuentes F, Moreno JA, Bellido C, Perez-Martinez P, Lozano A, Gómez P, Jiménez Y, Pérez Jiménez F. Phenolic content of virgin olive oil improves ischemic reactive hyperemia in hypercholesterolemic patients.J Am Coll Cardiol. 2005;46:1864-8.

-Schröder H, Covas MI, Marrugat J, Vila JS, Pena A, Alcántara M, Masià R. Use of a three-day estimated food record, a 72-hour recall and a food-frequency questionnaire for dietary assessment in a Mediterranean Spanish population. Clin Nutr 2001; 20: 429-37.

-Suarez M, Macia A, Romero MP, Motilva MJ. Improved liquid chromatography tandem mass spectrometry method for the determination of phenolic compounds in virgin olive oil. J Chromatography A. 2008, 1214 (1-2) 90-99.

-Suarez M, Romero MP, Ramo T, Macià A, Motilva MJ. Methods for preparing phenolic extracts from olive cake for potential application as food antioxidants. J Agric Food Chem 2009, February 25 issue (in press).

**4.1 CHRONOGRAM MODEL (EXAMPLE)**

This chronogram must indicate the persons involved in the project, including those contracted with project funds.

Underline the name of the person responsible of each task.

| Tasks | Centre | Persons | First Year (*)  Month | Second Year (*)  Month | Third Year (*)  Month |
| --- | --- | --- | --- | --- | --- |
|  |  |  | **1 2 3 4 5 6 7 8 9 10 11 12** | **13 14 15 16 17 18 19 20 21 22 23 24** | **25 26 27 28 29 30 31 32 33 34 35 36** |
|  |  |  | x | x | x | x |x | x |x | x | x |x |x |x | x | x |x | x |x | x | x | x | x | x | x |x | x |x | x | x |x |x | x | x | x | x | x |x |
| **Task 1. Functional**  **Olive Oils Preparation (I)** | ULL | MP Romero  M Suarez  A Macia  T Ramo  MJ Motilva | x | x | x |  |  |
| **Task 2 Dose-Response**  **Study** | URV | R. Solà  M.Giralt  RM. Valls  R Albaladejo . | x | x | x | x |x | x | |  |  |
| **Task 3 Bioavailability**  **Studies (I)** | ULL  IMIM | MJ Motilva  A Macia  A Serra  R. de la Torre  O. Castañer  Contracted FP2 | x | x |x | x | x |x |x |x | x | x |x |  |
| **Task 4. Bioactivity**  **Studies (I**) | URV  IMIM | M Romeu  S. Fernández  U. Catalán  RM Valls  R Albaladejo  Olga Castañer  Contracted FP2 | x | x |x | x | x |x |x |x | x | x |x |  |
| Tasks | Centre | Persons | First Year (*)  Month | Second Year (*)  Month | Third Year (*)  Month |
|  |  |  | **1 2 3 4 5 6 7 8 9 10 11 12** | **13 14 15 16 17 18 19 20 21 22 23 24** | **25 26 27 28 29 30 31 32 33 34 35 36** |
|  |  |  | x | x | x | x |x | x |x | x | x |x |x |x | x | x |x | x |x | x | x | x | x | x | x |x | x |x | x | x |x |x | x | x | x | x | x |x |
| **Task 5. Selection of**  **The best FOO1**  **(Functional olive oil 1)** | ULL  URV  IMIM | MJ Motilva  A Serra  Rosa Solá  M. Giralt  M.Cladellas  M.I. Covas |  | | x | x | |  |
| **Task 6. Functional**  **Olive Oil Preparation**  **(II)** | ULL | MP Romero  T Ramo  Contracted PhD  M Suarez  A Macia  MJ Motilva | | x |x | x |x | x | x |x |x |x | x | x |x | x |x | x |  |
| **Task 7. Sustained**  **Consumption Trial** | IMIM  I | M. Cladellas  M.I. Covas  Contracted  Nutritionist  Contracted Nurse  Contracted FP2  R. de la Torre  O. Castañer |  | x | x |x | x | x | x | x | x | x |x |  |
| **Task 8. HDL funcionality and bioactivity** | URV  IMIM | All URV  Investigators  O. Castañer  M.I. Covas  Contracted FP2 |  | | x | x | x | x |x | x |x | x | x |x |x | x | x |x |
| Tasks | Centre | Persons | First Year (*)  Month | Second Year (*)  Month | Third Year (*)  Month |
|  |  |  | **1 2 3 4 5 6 7 8 9 10 11 12** | **13 14 15 16 17 18 19 20 21 22 23 24** | **25 26 27 28 29 30 31 32 33 34 35 36** |
|  |  |  | x | x | x | x |x | x |x | x | x |x |x |x | x | x |x | x |x | x | x | x | x | x | x |x | x |x | x | x |x |x | x | x | x | x | x |x |
| **Task 9. Database**  **mangement** | IMIM | O. Castañer  Statician* | x | x | x | x |x | x |x | x | x |x |x |x | x | x |x | x |x | x | x | x | x | x | x |x | x |x | x | x |x |x | x | x | x |
| **Task 10. Statistical**  **Analyses** | IMIM | Statician*  MI Covas |  | x | x |x | x | x | x | x | x | x |x | x |x | x | x |x |x | x | x | x | x |
| **Task 11. Dissemination**  **of**  **The Results** | ULL  URV  IMIM | All investigators  of all Institutions |  | x | x | x | x |x | x |x | x | x |x |x | x | x | x | x | x |x |

(*) Mark an X inside the corresponding boxes (months)

*Contracted as an External Service when required

**5. BENEFITS DERIVED FROM THE PROJECT, DIFUSION AND EXPLOTATION OF RESULTS**

(maximum **1** page)

The following items must be described:

- Scientific and technical contributions expected from the project, potential application or transfer of the expected results in the short, medium or large term, benefits derived from the increase of knowledge and technology.
- Diffusion plan and, if appropriate, exploitation plan of the results.

As has been mentioned before, the global aim of the this project is to assess whether functional olive oils, enriched both with their own phenolic compounds or also with them plus complementary phenolic compounds from thyme, could act as nutraceuticals concerning the quantity and quality (functionality) of the human high density lipoprotein (HDL) *in vivo*.

Our hypothesis is that the functional olive oils tested will be protective on the oxidative lipid damage and will increase the quantity and functionality of the HDL lipoprotein, particularly the thyme spiced olive oil. If our hypothesis are accomplished these data will constitute the scientific basis for a claim to consider these functional olive oils as nutraceuticals, thus permitting health claims to be made. A patent (s) will be developed in order to commercialize the functional olive oil through an associated olive oil industry.

The project includes the assessment of several potential mechanisms for increasing the HDL quantity and quality. This is a current goal for therapeutic options against coronary heart and vascular diseases. The positioning of a nutraceutical in this area will open new insights both in the guidelines for risk patients management (in a similar way that sterol-rich food is recommended as a second step in the management of hyperlipemia), and in the search for nutraceutical foods other than olive oils. This will open a new field in the functional food area.

The Diffusion plan is referred to in the Task 11 of the Project. Within the Project, the generation of scientific manuscripts is included. Also, and for the general public, a divulgative manuscript to be sent to Consumers Associations will be prepared, as well as a leaflet to be sent to the media (press, TV, and supermarket publications (i.e. Eroski)). We have not included a web site within the frame of the Project due to the doubts that in recent years have been aroused on this issue concerning its efficacy for Project results dissemination among the general public. However, a web for the project can be included if the reviewers of the Project, and the area policy makers, think it would be useful as part of the exploitation plan of the results.

**6. BACKGROUND OF THE GROUP**

**(In the case of a coordinated project the topics 6. and 6.1. must be filled by each partner)**

(maximum **2** pages)

- **Indicate the previous activities and achievements of the group in the field of the project:**

If the project is related to other previously granted, you must indicate the objectives and the results achieved in the previous project.

If the project approaches a new research field, the background and previous contributions of the group in this field must be indicated in order to justify the capacity of the group to carry out the project.

**SUBPROJECT 1. Instituto Municipal de Investigaciones Médicas (IMIM)**

The Main Areas in which the Cardiovascular Risk and Nutrition Research Group of the Instituto Municipal de Investigación Médica (IMIM-Hospital del Mar) has developed its scientific activity are:

1) Intervention studies

- 1. Olive Oil Studies:

Antioxidant effect of Olive Oil and Bioavailability of olive oil phenolic compounds in humans: In 1998 Partners from the IMIM-Hospital del Mar (P.I.. Dr. M.I. Covas, Subproject 1) started the study: “Antioxidant Effect of Olive Oil: A Multidisciplinary Study. This study has been supported by the Spanish Grants CICYT (1997, 2002) and FEDER (1999). The acquired expertise permitted us to promote the European Project: “The effect of olive oil consumption on oxidative damage in European populations: The EUROLIVE Study (QLK1-CT-2001-00287.Coordinator: Dr. M.I. Covas, Coordinating Centre: IMIM-Hospital del Mar)”. Besides bioavailability studies of phenolic compounds (PC) from olive oil (OO) in humans, the project included the performance of 6 randomized, crossover, controlled clinical trials (Denmark, Finland, Germany (2), Italy, and Spain) with 3 similar OOs, but with differences in their PC content in 200 healthy volunteers.

From our results we have been able to establish:1) the high antioxidant capacity of OOs with a high PC content (virgin), and the influence of the type of PC present in the oils on such antioxidant capacity (Med Clin 2000; Lipids 2000); and 2) the antioxidant capacity on the *ex vivo* LDL oxidation of the whole content of PC present in the virgin OO (Lipids 2000) and that of isolated PC from OO as tyrosol (Int Pharmacol Res 2000). In human studies we have reported the capacity of PC from OO and its metabolites to bind human LDL *in vivo* (J Chromatogr A 2006; Anal Chim Acta 2007) as well as the protective effect of olive oil PC over other PC bound to human LDL (Biol Res 2004) in a dosis-dependent manner of the PC content of the OO administered (Free Rad Biol Med 2006; Br J Nutr 2007). We characterized the bioavailability of tyrosol and hydroxytyrosol, major OO phenolics, in humans (Drugs Exp Clin Res 2003; Clin Chem 2003; Eur J Clin Nutr 2003) from real-life doses of natural olive oils. In postprandial studies we established :1) a 25 mL dose of any type of OO do not promote oxidative stress (Drugs Exp Clin Res 2004); and 2) doses of 40 mL and 50 mL of any type of OO promote oxidative stress, which is modulated by the PC of the OO (Lipids 2001; Free Rad Biol Med 2006). The anti-thrombotic effect of OO with high PC content has also been assessed (Am J Clin Nutr 2007). In the frame of short-term effects (1 week) we have reported:1) an increase in the antioxidant content in LDL (Eur J Nutr 2000) after virgin OO consumption; and 2) a decrease in the *in vivo* LDL and DNA oxidation, and an increase in glutathione related enzymes, in a dose-dependent manner with the PC of the OO administered (J Nutr 2003). Concerning sustained effects of OO consumption (3 weeks), in randomized, crossover, controlled studies with healthy volunteers (Eur J Nutr 2004) and stable CHD patients (Atherosclerosis 2005; Eur J Clin Nutr 2007), with an established high degree of oxidative stress (Atherosclerosis 2003), we described a dose-dependent decrease of the *in vivo* LDL oxidation, systolic blood pressure, and inflammatory markers, with the phenolic content of the olive oil administered. Results of the EUROLIVE Study published in Ann Int Med 2006, Free Rad Biol Med 2006, FASEB J 2007, J Nutr 2007, and J Am Coll Nutr 2007, showed the capacity of olive oil for: 1) counteracting DNA oxidation, without changes in DNA adduct formation, and increasing the reduced glutathione content in a similar way in all types of OO; 2) increasing the HDL cholesterol and decreasing the lipid oxidative damage in a dose-dependent manner with the phenolic content of the OO administered; 3) increasing the oleic acid content in: i) LDL, in an inverse relationship with the degree of lipid oxidative damage, and ii) plasma, in an inverse relationship with the systolic blood pressure levels in non-Mediterranean participants.

1.2 The effect of the Mediterranean Diet in the primary prevention of cardiovascular disease. The PREDIMED Study.

This project starts with the Network of Research Groups granted by ISCIII (G03/045) and is followed in the frame of the CIBER of Fisiopatología de la Obesidad y la Nutrición (CIBEROBN,CB06/03) and the RETIC (RD06/0045/0000). The Project involves the recruitment of 7,500 individuals, currently recruited, at high cardiovascular risk and the assessment of the effects of two variants of the Mediterranean Diet (rich in virgin olive oil and rich in dry fruits) on primary (disease development) and secondary (lipids, lipid oxidation, inflammatory markers…) end points for cardiovascular disease. In this project, investigators from 18 Research Groups of 7 Autonomous Communities of Spain, are included. Results of the PREDIMED study at 3-months of intervention showed the beneficial effect of the Mediterranean-type diet on cardiovascular risk factors (Ann Int Med 2006), including the degree of *in vivo* LDL oxidation (Arch Int Med 2007), particularly in participants consuming the Mediterranean Diet enriched with virgin olive oil. According to this, an inverse relationship between the adherence to the Mediterranean Diet and inflammation markers at baseline has been reported in a subsample of 883 individuals (Eur J Clin Nutr 2007). The benefits of a Mediterranean diet enriched with nuts on metabolic syndrome features have been recently reported (Arch Int Med 2009).

- 1. Nutrigenomics: Molecular mechanisms involved in the protective effect of olive oil and the Mediterranean Diet and consumption.

Within the frame of the EUROLIVE (CICYT, SAF 2004-08173-C03-00) and PREDIMED (FIS PI04 1308) studies we are developing a line of research on human *in vivo* gene expression associated to The Mediterranean diet and olive oil and its PC consumption. As a first step, several procedures for mRNA extraction were compared in order to select the best for application (Anal Biochem 2005). As a previous methodological step we have evaluated several of sources of inter- and intra-individual variability gene expression (Clin Biochem, 2008). The transcriptome response after virgin olive oil consumption has been recently assessed (OMICS 2009, in press).

2) Nutritional epidemiology

With data from population studies (the REGICOR study, FIS 1993 and 1994; and HERMES, FIS 2002) we have assessed associations between nutrients, food, or dietary patterns and risk factors for coronary heart disease (CHD)(Br J Nutr 2002 and 2003; Eur J Nutr 2002 and 2004; Eur J Clin Nutr 2004 and 2007; Ann Nutr Metab 2005; Free Rad Biol Med 2006) in individuals aged 24-74 years. The food questionnaires used in all our studies have been previously validated (Clin Nutr 2001). Our results show the efficacy of healthy dietary patterns as a tool for the management of CHD risk factors. Lately we focused on obesity (J Nutr 2004;Am J Clin Nutr 2006; Int J Obes 2006; Obesity 2007; Br J Nutr 2007 (2); Eur J Nutr 2007) as a major CHD risk factor.

**SUBPROJECT 2. Lleida University (ULL)**

The Main Areas in which the **Antioxidants Research Group of the Departament of Food Technology of the University of Lleida** (**Subproject ULL**) has developed its scientific activity are: the antioxidants field and virgin olive oil. The studies carried out during the last five years (period 2005-2008) have been diverse and have supposed a great challenge for our investigation group. During this time, our group has reoriented its objectives on: firstly, the development and validation of the analytical methods for the characterization and quantification of phenolic compounds present in vegetal extracts; and secondly, the bioavailability evaluation of phenols by developing analytical methods for the analysis of their native forms and their metabolites in human biological fluids. Next, a brief summary of the obtained main results is presented.

The evaluation of the potential antioxidant the individual components of the phenolic fraction of virgin olive oil has been carried out inside the activities of the before financed project AGL2005-07881-C02 (*Enriched olive oil enriched in phenolic compounds. Obtaining purified phenols, evaluation of its antioxidant potential and development of a prototype of enriched olive oil*). These phenolic compounds have been purified from the virgin olive oil. The results of different models of evaluation of their antioxidant capacity have showed a significant biologic potential for the secoiridoides and flavonoids and synergy effects between them. Taking into account the results of different models *in vitro*, the phenolic extract obtaining from the main olive mill by-product, the pomace, has been optimized. This extract has been used for the development of a prototype of an enriched olive oil in phenolic compounds with the aim of enhancing its benefits in preventing cardiovascular diseases. At present, this oil is being tested in a clinical study with a group of 30 volunteers. This study is coordinated with the Unit of Lipids and Atherosclerosis School of Medicine, University Rovira i Virgili.

In the field of antioxidants and the development of functional foods, our group participates since 2006 in a project within the CENIT Program (Ingenio 2010) entitled "Methodology for the design, evaluation and validation of functional foods in the prevention of cardiovascular disease and Alzheimer's (MET-DEV-FUN). The consortium is led by business Morella Nuts, SL. (Reus) and participate in the consortium the following companies: Nabisco Inc.; BTSA, Applied Biotechnology, SL; Selection Vaig battle, S. a.; Pecuaria Industrial Technology, SA; Neuron BioPharma, SA; Shirota Functional Foods, SL; Llet Grup Pascual, ODS; Innaves, SA. The consortium activities of the research group are directly related with La Morella Nuts and Shirota Functional Foods. The incorporation in a project the size and economic activity of this program CENIT, has supposed a significant challenge in the dynamics of work. This new dynamic requires a rapid response to the needs of the company at any time and a commitment of confidentiality, which is assuming a certain reduction in productivity in terms of scientific publications. The work of this collaboration have been focused on optimizing the conditions for obtaining phenolic extracts from different plant sources, such as cocoa, carob flour, oilseeds and byproducts of wine industry. Different variables, solutions and parameters for the phenolic extraction have been evaluated, so that the results have been transferred to the industry and have served as the basis for the semi-industrial scale. Therefore this activity related to the acquisition of phenolic extracts from different plant sources supports the group's ability to carry out Tasks 1 and 6 of this proposal (Preparing functional oils FOO1 and FOO2).

**Thanks to financial support from the CENIT project has been able to acquire a large infrastructure of liquid chromatography UPLC-ESI-MS/MS. This infrastructure has enabled the development and validation of analytical methods and quantification of phenolic compounds by liquid chromatography with tandem MS.** **The phenolic groups have been considered: hydroxybenzoic acids, acids hydroxycinamics, flavonols, flavanols and lignans. In this line also has validated the methodology for determination of procyanidins and its metabolites in plasma samples.** **All these analytical methodologies have been used to support the activities of the various companies within the CENIT project, and in any case have provided the basis for the implementation of these methods in the laboratory of the company.** **The experience of the group shows their ability to take for granted successfully with the Tasks 2, 3, 4, and 5 of this proposal (Dose-response study and phenol Bioavailability studies).**

**In connection with international research groups, in recent years have carried out the following contributions:**

- **Canadian Grain Commission (Group Dr. Véronique J. Barthet)
  Grain Research Laboratory - Laboratoire de Recherche des Grains (Winnipeg, Canada). Activity: Methods for extracting and evaluating the potential of antioxidant lignans in flax seeds.**
- **Institute of Food Research (Dr Paul A Kroon, Polyphenols and Health). Norwich Research Park (Norwich, UK). Activity: Assessment of intestinal transport and metabolism of olive oil phenols in CaCo2 cell model.**
- Critical Fluids Technology & Separations Laboratory Department of Chemical Engineering (Professor Jerry W. King). University of Arkansas (Fayetteville, USA)

Activity: The focus of the research would involve the investigation of several processing methods and systems designed to extract and fractionate polyphenolic-containing foodstuffs and natural product, with a particular focus on grape pomace as a side-stream product from the juice and wine production industries.

Through this partnership will greatly improve efforts to obtain the phenolic extract (Tasks 1.1. and 6.1.).

This collaboration is carried out through a stay of 6 months (June-December 2009) of a member of our research group (Manuel Suarez Recio). Manuel Suarez is currently enjoying an FPU scholarship attached to the project AGL2005-07881-C02-01, which ends in December 2010. Since this project is now completed, the investigator will join full-time to the project being requested.

**SUBPROJECT 3. Rovira and Virgili University (URV).**

The **Diet, Nutrigenetics and Health Group**, of Universitat Rovira i Virgili, Faculty of Medicina, Hospital Universitari Sant Joan, Reus, is made up of components of the research groups groups of “Farmacobiologia cel·lular” and the " Unitat de Recerca de Lípids i Arteriosclerosi " work in collaboration to improve scientific evidence of nutritional influence in chronic disease, particularly, in cardiovascular disease. **Previous relevant experience:** the main areas where this new multidisciplinary group has developed its scientific activity are:1) human health benefits of dietary compounds such as soluble fiber, cocoa products, and phenolic compounds from virgin olive oil; the specific focus being on cardiovascular disease and methods to value the antioxidant capacity of biological fluids. 2) The group expertise embraces molecular mechanisms, toxicology and human dietary intervention trials of ingested bioactive compounds. Also, the group has expertise in *in vivo* experimental animal studies into mechanisms of acute toxicity, long-term effects and molecular mechanisms of bioactive compounds. Researchers of both groups are also involved in the project 3118-CDTI CENIT of methodologies for the design, evaluation, and validation of functional food in the prevention of cardiovascular diseases and of the Alzheimer and project EdAl of education in nourishment in the children’s population.

The cacao products study provided the patents:

**Inventor/s (signature):** Ramírez B, Anglès MN, Reguant J, Solà R, Godàs G,

**Title:** Alimento funcional con efectos positivos en la prevención de enfermedades cardiovasculares. **Application number:** 20080487 **First priority country:** UNITED STATES **Date of priority:** 2008 **Patent code:** 000243. **Inventor/s (signature):** Ramírez B, Anglès MN, Reguant J, Solà R, Godàs G,

**Title:** Alimento funcional con efectos positivos en la prevención de enfermedades cardiovasculares. **Application number:** 200502963 **First priority country:** SPAIN **Date of priority:** 2007.**Patent code:** 000230. **Inventor/s (signature):** Ramírez B, Anglès MN, Reguant J, Solà R, Godàs G. **Title:** Alimento funcional con efectos positivos en la prevención de enfermedades cardiovasculares.**Application number:** 20060788 **First priority country:** EUROPE **Date of priority:** 2007. **Patent code:** 000231

Currently, the process of the patents about the effects of *Plantago ovata* husk, soluble fiber on cardiovascular biomarkers are in course.

In AGL2005-07881-C02-02, PROJECT TITLE: Virgin olive oil enriched with phenolic compounds in order to improve cardiovascular protection. The results are submitted to the editor: Human plasma virgin olive oil phenolic compounds detection with synergistic antioxidant effect. At present we are involved in a human intervention study to test an olive oil enriched with 1000 ppm , elaborated by Dr Mª J Motilva (AGL2005-07881-C02-01), as a anti-hypertensive food.

Molecular and cellular bases of the oxidative damage in ageing. G03/137

Evaluation of the ingestion of dried fruits on the caloric balance, the metabolic and inflammatory responses and the oxidation. AGL2005/03605/ALI.

Prooxidant activity of the aluminium in transgenic mice. Evaluation of the protective role of melatonin on the antioxidant enzymes gene expression. PI 050622.

Effects of iron supplementation during pregnancy on the mother and neonatal health according to mutations of the HFE gene of the hereditary hemochromatosis. PI052462.

Nowadays, both groups take part in 3118-CDTI CENIT project: Methodologies for the design, evaluation and validation of functional food in the prevention of cardiovascular diseases and of the Alzheimer. MET-DEV-FUN. Period: 2006-2009 IP: Rosa Solà and Mª José Motilva,

Recent relevant publications related to the project:

Alvaro A, Solà R, Rosales R, Ribalta R, Anguera A, Masana L, Vallvé JC. Gene Expression Analysis of a Human Enterocyte Cell Line Reveals Downregulation of Cholesterol Biosynthesis in Response to Short-chain Fatty Acids. IUBMB Life, 2008; 60(11): 757–764.

Cabre JJ, Martin F, Costa B, Pinyol JL, Llor JL, Ortega Y, Basora J, Baldrich M, Sola R, et al. Metabolic Syndrome as a Cardiovascular Disease Risk Factor: Patients Evaluated in Primary Care. BMC Public Health 2008, 8:251 doi:10.1186/1471-2458-8-251

Sola R, Godas G, Ribalta J, Vallve JC, Girona J, Anguera A, Ostos M, Recalde D, Salazar J, Caslake M, Martin-Lujan F, Salas-Salvado J, Masana L. Effects of soluble fiber (Plantago ovata huso) on plasma lipids, lipoproteins and apolipoproteins in men with ischemic Heart disease. Am J Clin Nutr 2007; 85 : 1157- 1163

Sundl I, Guardiola M, Khoschsorur G, Sola R, Vallve JC, Godas G, Masana L, Maritschnegg M, Meinitzer A, Cardinault N, Roob Jm, Rock E, Winklhofer-Roob Bm, Ribalta J. Elevated concentrations of circulating vitamin E in carriers of the apolipoprotein A5 gene-1131T>C variant and associations with plasma lipids and lipid peroxidation. J Lipid Res 2007; 48: 2506- 2513

Fito M, Guxens M, Corella D, Saez G, Estruch R, De La Torre R, Frances F, Cabezas C, Lopez-Sabater Mdel C, Marrugat J, Garcia-Arellano A, Aros F, Ruiz-Gutierrez V, Ros E, Salas-Salvado J, Fiol M, Sola R, Covas MI; for the PREDIMED Study Investigators. Effect of a traditional Mediterranean diet on lipoprotein oxidation: a randomized controlled trial. Arch Intern Med 2007; 1195-1203

Drissi A, Girona J, Cherki M, Godàs G, Derouiche A, El Messal M, Saile R, Kettani A, Solà R, Masana L, Adlouni A. Evidence of hypolipipemiant and antioxidant properties of argan oil derived from the argan tree (Argania spinosa). Clinical Nutrition 2004;23:1159-1166

Girona J, La Ville AE, Solà R, Motta C, Masana L. HDL derived from the different phases of conjugated diene formation reduces membrane fluidity and contributes to a decrease in free cholesterol efflux from human THP-1 macrophages. Biochimica et Biophysica Acta. Mollecular and Cell Biology of Lipids 2003;1633:143-148

Giralt M, Nogués MR, Alomar A, Argany N, Calvo CG, Mallol J. Human oxidative stress during PUVA therapy. Protective effect of a Polypodium leucotomos extract. Meth Find Exp Clin Pharmacol 22 (6): 496.(2000).

Gómez, M.; Esparza, J.L.; Nogués, R.; Giralt, M.; Cabré, M.; Domingo, J.L. Pro-oxidant activity of aluminum in the rat hippocampus. Gene expression of antioxidant enzymes after melatonin administration. Free Radical Biology and Medicine 38(1)**:** 104-111 (2005)

Nogués MR, Giralt M, Romeo M, Mulero M, Sanchez-Martos V, Rodríguez E, Acuna-Castroviejo D, Mallol J. Melatonin reduces oxidative stress in erythrocytes and plasma of senescence-accelerated mice. Journal of Pineal Research 41(2)**:** 142-149 (2006)

Mulero M, romeo M, Giralt M, Folch J, Nogués MR, Fortuno A, Sureda FX, Linares V, Cabre M, Paternain JL, Mallol J. Oxidative stress-related markers and largerhans cells in a hairless rat model exposed to UV raditation. Journal of Toxicology and Environmental Health 69 (14)**:** 1371-1385 (2006)

Martínez-Vea A, Marcas L, Giralt M, Bardají A, Gutiérrez C, Romeo M, García C, Compte T, Peralte C, Mallol J, Oliver JA. Oxidative stress and cardiovascular effects of anemia treatment with erythropoietin in predialysis patients with chronic kidney disease. Neprhrology Dialysis Transplantation. 21**:** 147-148 (2006)

Esparza JL, Gómez M, Nogués MR, Paternain JL, Mallol J, Domingo JL. Melatonin reduces oxidative stress and increases gene expression in the cerebral cortex and cerebellum of aluminum-exposed rats. J Pineal Res 39: 129-136 (2005)

Nogués MR, Giralt M, Romeu M, Mulero M, Sánchez-Martos V, Rodríguez E, Acuña-Castroviejo D, Mallol J. Melatonin reduces oxidative stress in erythrocytes and plasma of senescence-accelerated mice (SAM). J Pineal Res 41:142-149 (2006)

Mulero M, Rodríguez-Yanes E, Nogués MR, Giralt M, Romeu M, Gonzalez S, Mallol J. Polypodium leucotomos extract inhibits glutathione oxidation and prevents Langerhans cell depletion induced by UVB/UVA radiation in a hairless rat model. Exp Dermatol 17 (8):653-658 (2008)

Espía M, Sebastián C, Mulero M, Giralt M, Mallol J Celada A, Lloberas J. Granulocyte macrophage-colony-stimulating factor-dependent proliferation is impaired in macrophages from senescente-accelerated mice. J Ger A Bio Sci Med 63(11): 1161-1167 (2008)

**6.2. PUBLIC AND PRIVATE GRANTED PROJECTS AND CONTRACTS OF THE RESEARCH GROUP**

Indicate the project and contract grants during the last 5 years (2004-2008) (national, regional or international)

Include the grants for projects under evaluation

| Title of the project or contract | Relationship with this proposal (1) | Principal Investigator | Budget | Funding agency and project reference | Project period  (2) |
| --- | --- | --- | --- | --- | --- |
|  |  |  | EUROS |  |  |
| EUROLIVE: The effect of olive oil consumption on oxidative damage in Euroopean populations. | 1 | Covas MI | 412950 | EU | C  2002-2005 |
| Factores dietéticos relacionados con la aparición y protección frente al infarto miocardio: Un estudio de casos y controles. | 2 | Schröder H | 29500 | FIS.ISCIII  Reference: CP03/00115 | C  2007-2009 |
| Mecanismos moleculares involucrados en el papel protector del aceite de oliva frente al desarrollo de los procesos ateroscleróticos. | 1 | Covas MI | 155100 | MCYT  SAF2004-08173-C03-00 | C  2003-2006 |
| Efecto de la Dieta tipo Mediterráneo en la prevención primaria de la enfermedad cardiovascular. Red temática de Grupo. Red estudio PREDIMED | 1 | Coordinator: Estruch R  IP IMIM-Hospital del Mar: Covas MI | 22134 | FIS.ISCIII  PI051726 | C  2006 |
| Multiple new systemic phenotypic biomarkers for the prediction of first major cardiovascular events in a high cardiovascular risk population. Modulation by a Mediterranean-type diet. | 2 | Coordinator: Estruch R  IP IMIM-Hospital del Mar: Covas MI | 240358 | MINISTERIO DE SANIDAD Y CONSUMO  CNIC-06 | C  2008-2011 |
| NEW sets of biomarkers of food-DErived FAs and poLyphenols  NEW DEAL | 1 | Coordinator:Dr Licia IACOVIELLO  IP IMIM-Hospital del Mar: Covas MI | 693288 | EU | S  2009-2014 |
| Chlorophyllic and carotenoid fractions of oils from Les Garrigues (Lleida). | 3 | Mª Paz Romero  UdL | 58748 | AGL 2001-0550 | C  28/12/01 to 27/12/04 |
| Phenolic fraction of olive oil: identification, functionality and transfer from the fruit to the oil. | 1 | Mª José Motilva  UdL | 51750 | AGL 2002-0289 ALI | C  31/12/2002  30/12/2005 |
| Trade agreement University of Lleida-AOC Les Garrigues Council: Quality control of olive oil process and inspection tasks. | 3 | Mª Paz Romero/  Mª José Motilva  UdL | 30000 | SEDAI Chemical and Sensory Quality In Foods | C  2000/04 |
| Volatile compounds in olive oils from Arbequina cultivar.  DOP Les Garrigues | 3 | Mª Paz Romero  UdL | 14950 | AGL 2004-06158 | C  13/13/2004  13/12/2005 |
| Olive oil enriched with phenolic compounds. Pure phenols obtaining, evaluation of their antioxidant capacity and development of an enriched olive oil prototype | 1 | Mª Josefa Motilva  UdL | 80000 | AGL2005-07881-C02-01/ALI | C  2005 to 2008 |
| Protean peroxidative lesion of neuronal tissues. An undesirable effect of omega–3 fatty acid supplemented diet? | 3 | Manuel Portero Otín  Experimental Medicin Department.  UdL | 102600 | AGL2006-12433/ALI | C  2006 to 2008 |
| Methodology for designing, evaluating and validating the functional foods in the CV and Alzheimer diseases prevention. | 1 | Trade Association:  La Morella Nuts,S.A.  UdL Mª José Motilva | 1411980 | CENIT  MET-DEV-FUN | C  2006 to 2009 |
| Metabolism of chlorophylls and phenols in olive fruits: involvement in the intrinsic profile of minor components in virgin olive oil according to variety, origin and cultivation system. | 1 | Mª Paz Romero  UdL | 68000 | AGL 2007-66139-  C02-02-ALI | C  2007-2010 |
| Network in spices research | 2 | Gonzalo Alonso  Díaz-Marta  Un. Castilla-La Mancha | 20000 | AGL-2006-27834-E/ALI | C  2007-2008 |
| Establecimiento de metodologías para el diseño, evaluación y validación de Alimentos funcionales contra el Alzheimer y las enfermedades cardiovasculares. LA MORELLA NUTS, S.A. MET-DEV-FUN | 1 | Rosa Solà Alberich  URV | 140000 | CENIT – Ministerio de Industria, Turismo y Comercio | C  2006-2009 |
| Aceite de oliva enriquecido en compuestos fenólicos para optimizar la protección cardiovascular. Del consumo a los efectos protectores cardiovasculares  AGL2005-07881-CO2-02/ALI | 1 | Rosa Solà Alberich  URV | 100000 | Dirección General de Investigación | C  2005-2008 |
| Ensayo clínico multicentrico, comparativo, doble ciego, paralelo de dos vias de los efectos del tratamiento con cutílculas de semillas de plantago ovata sobre el perfíl lípidico en pacientes afectos de colesterolemia. Fase IV | 1 | Rosa Solà Alberich  URV | 306000 | Empresa MADAUS S.A.  Departament d’industria de la Generalitat de Catalunya | C  2005-2007 |
| Molecular and cellular bases of the oxidative damage in the aging. (Thematic networks of research groups) | 1 | Jordi Mallol  URV | 28670 | Instituto de Salud Carlos III  G03/137 | C  2003-2006 |
| Evaluation of the consumption ingestion of dried fruits on the caloric balance, the metabolic and inflammatory response and the oxidation. | 0 | Jordi Salas  URV | 45000 | CICYT  AGL2005/03605/ALI | C  2005-2008 |
| Prooxidant activity of the aluminium in transgenic mice. Evaluation of the protective role of melatonin on the antioxidant enzymes gene expression. | 1 | Mercedes Gómez  URV | 63070 | FIS. Instituto de Salud Carlos III.  PI 050622 | C  2005-2008 |
| Effects of iron supplementation during pregnancy on the mother and neonatal health according to mutations of the HFE gene of the hereditary hemochromatosis. | 1 | Victoria Arija  URV | 76755 | PI052462 | C  2005-2008 |
| Biomarkers of Robustness of Metabolic Homeostasis for Nutrigenomics-derived Health CLAIMS Made on Food. | 1 | Andreu Palou  Universitat Illes Balears |  | Competitive European Regions through research and innovation -Seventh     Framework Programme (FP7) | S |
| Health promoting mechanisms of polyphenols from fruit and vegetables. | 1 | Cinta Bladé  URV |  | Competitive European Regions through research and innovation -Seventh     Framework Programme (FP7) | S |

1. Write 0, 1, 2 or 3 according to: 0 = Similar project; 1 = Very related; 2 = Low related; 3 = Unrelated.

(2) Write C or S if the project has been funded or it is under evaluation, respectively.

**7. TRAINING CAPACITY OF THE PROJECT AND THE GROUP**

**(In the case of Coordinated Projects this issue must be filled by each partner)**

This title must be filled only in case of a positive answer to the corresponding question in the application form.

Justify that the group is able to receive fellow students (from the Suprograma de Formación de Investigadores) associated to this project and describe the training capacity of the group. In the case of coordinated projects, each subproject requesting a FPI fellowship must fill this issue.

Note that all necessary personnel costs should be included in the total budget requested. The available number of FPI fellowships is limited, and they will be granted to selected projects as a function of their final qualification and the training capacity of the groups.

**SUBPROJECT 1. Instituto Municipal de investigaciones Médicas (IMIM)**

The Cardiovascular Risk and Nutrition Research Group of the Institut Municipal d´Investigació Mèdica (IMIM-Hospital del Mar) is well suited to receive fellow students. The link of the IMIM with the Pompeu Fabra University and that of the Hospital del Mar with the Barcelona Autonomic University facilitates the Master and PhD Programs of the fellows. On schedule, multiple scientific sessions are performed both in the frame of the IMIM and also in the frame of the Parc de Recerca Biomèdica de Barcelona (PRBB)(which comprises, the Biology Faculty of the UPF University , the Genomic Regulation Center (CRG) etc.). Also our Research Group has specific scientific sessions every 2 weeks. In all type of scientific session national and international speakers out from the PRBB are commonly invited.

The following PhD thesis has been performed in the frame of our group in the last years:

-Interacción de la MDMA con la paroxetina en humanos. (*Interaction between paroxetin and MMDA in humans)*. María Segura. University Pompeu Fabra. Year 2004: Director: Dr. R. de la Torre

-Depuración enantioselectiva de la MDMA en humanos . *(Enantioselective depuration of MDMA in humans)*. Neus Pizarro. Pizarro Neus. Pompeu Fabra University. Year 2004. Director: Dr. R. De la Torre

-Efectos Antioxidantes del Aceite de Oliva y sus compuestos fenólicos (*Antioxidant effects of olive oil and its phenolic compounds)* . Montserrat Fitó Colomer. Autonomic University of Barcelona. Year 2003. Director: Dr. M.I. Covas

- Distribution of 3,4-methylenedioxymethamphetamine (MDMA) in non conventional matrices and its applications in clinical toxicology. Simona Pichini. Barcelona Autonomic University. Year 2005. Director: Dr. R. De la Torre. Co-director: Dr. M.Farré

- La oxidación lipídica en el riesgo de infarto agudo de miocardio: una aproximación poblacional (*Lipid oxidation and myocardial infarction risk: a population approach).* Mónica Guxens Junyent. Autonomic University of Barcelona. Year 2008. Director: Dr. M.I. Covas. Co-director: Dr. Jaume Marrugat

-Bloqueo bifascicular: Factores que influyen en la evolución a bloqueo auriculoventricular avanzado y en la mortalidad. (*Bifascicular blockage: Influencing factors for its evolution to an auricululoventricular blockage and mortality*) Julio Martí Almor. AutonomicUniversity of Barcelona. Year 2008. Director: Dr. M. Cladellas.

-Drug metabolism and pharmacogenetics as contributing factors to MDMA-induced toxicity in humans. (TD). Brian. O’Mahony . Pompeu Fabra University. Year 2008. Director: Dr. R. De la Torre.

The following PhD work is currently on-going:

# -Molecular mechanisms involved in the protective effect of the Mediterranean diet and olive oil consumption in humans. Valentini Konstantinidou. University Pompeu Fabra. Director: Dr. M.I Covas

-The transcriptomic effect of virgin olive oil and its phenolic compounds in European Popullations.Olha Khymenetz. University Pompeu Fabra. Director: Dr. Rafael de la Torre

Post-doctoral fellowships

Dr. Tanja Weinbrenner, a German nutritionist, had a Marie Curie Grant (2003-2004) fo do a fellowship in the Spanish Project of the Antioxidant Effect of Olive Oil and its Phenolic Compounds. Tutorial: Dr. M.I. Covas

Post-graduate fellowships.

In the frame of the European Program, Leonardo da Vinci (Leo Theses) we Program collaborators. Around every 6 months we receive a postgraduate student from the Food Technology Institut of Athens or from Patras University. Last students were: Kyriaki V Papantoniou (From: 1/10/2008; To: 31/03/2009); Aikaterini Antoniou (From: 24/03/2008 to 24/06/2008); Anna Kolliopoulou (From: 16/07/2007; To: 11/01/2008); and Ioannis Vouldis (From: 1/05/2006; To: 15/10/2006

**SUBPROJECT 2 (ULL). Unit of Antioxidants (University of Lleida)**

The extensive professional experience demonstrates the training capacity of the group. The main activities from last years are detailed below:

- Doctoral Thesis

The following PhD thesis has been performed in the frame of our group in the last years:

- Criado García, María Nieves (2005). Study of pigment profile of olives and olive oils from *Arbequina* cultivar grown in Les Garrigues area (Lleida-Spain). University of Lleida. Supervised by Motilva Casado, M.Josefa/Romero Fabregat, M. Paz

- Artajo Medina, Luz S. (2007). Phenolic Compounds: Their role during olive oil extraction and in flaxseed: Transfer and Antioxidant Function. University of Lleida. Supervised by Motilva Casado, M.Josefa/Romero Fabregat, M. Paz

The following PhD work is currently on-going (name and subject):

- Nadia Ortega Olivé. Bioactive compound from cocoa and other vegetable sources.

- Aránzazu Soler Cantero. Antioxidant activity of phenols from olive oil.

- Maria Casanovas Castro (MICINN-FPU grant holder) Topic: Pigment profile in relation to phenols.

- Aida Serra (University of Lleida grant holder) Topic: Bioavailability of bioactive compounds.

- Manuel Suárez (MICINN-FPI grant holder). Topic: Functional olive oil.

The quality of the formation is guaranteed by the qualityof the job placement of our postgraduate students. All PhD graduates are working in Departments of Quality or I+D of private companies.

- María Jesús Tovar de Dios. Malting plant of Damm, S.A. Lleida

- Nieves Criado García. Mercadona. València

- Luz Artajo Medina. The National Federation of Coffee Growers of Colombia.

- José Ramón Morelló Estupiñá. La Morella Nuts, S.A. Tarragona.

Recently, two PhD students has incorporated in I+D Departments, Nadia Ortega Olivé (La Morella Nuts, S.A. Tarragona) and Aránzazu Soler (Shirota Functional Foods, S.L. Tarragona)

- Post-grade formation

The group relies on the participation of associate professors with a large academic experience in Food Science and Technology. The Motilva and Romero professors are responsible of the subjects ‘Antioxidants and health’ and ‘Quality and composition of vegetal oils’ in the University Master's Degree in Agro-food systems by Research. The continuation for the students of this master is the completion of the Doctoral Thesis in any of the related doctoral programs. Also, the group participates in the Master in Management and Innovation in the Food Industry, a Master to provide the candidates of a specialization in the development of processes and food products, their quality assurance and surveillance of the safety and the strategies for their commercialization.

Also, in the laboratories of the Department of Food Technology are carried out many experimental works supervised by these teachers. The more important activities are the supervision of grade thesis to student of the School of Agricultural and Forestry Engineering specialized in Food Industries, but also in our laboratories there are students of the Nutrition and Dietetic grade doing their short period of formative practices and students of occupational training from Secondary Schools.

**SUBPROJECT 3. Rovira and Virgili University**

The following PhD thesis has been performed in the frame of our group in the last years:

Implementation of stable isotopes lipoprotein kinetic studies: effects on hdl metabolism of a mediterranean type diet rich in mufas from virgin olive oil. Katia ULIAQUE CUGAT, European Doctorate. Doctorado de Nutrición y Metabolismo, Facultad de Medicina y Ciencias de la Salud, Universidad ROVIRA I VIRGILI, 27 January 2007. Dra R SOLÀ.

Efectos de la fibra soluble cáscaras de *Plantago ovata* sobre factores lipídicos de riesgo cardiovascular. Anna ANGUERA VILA, Doctorado de Nutrición y Metabolismo, Facultad de Medicina y Ciencias de la Salud, Universidad ROVIRA I VIRGILI, Reus, 3 May 2007. Dra R SOLÀ**..**

The following PhD work is currently on-going:

Effects on diet on cardiovascular risk factors. Rosa Mª VALLS ZAMORA, European Doctorate. Training in Royal Infermery University of Glasgow, (from 03/2006 to 04/2006), COSPI-2 (Erasmus Intensive Programme “Combating Obesity: Strategies for Prevention and Intervention”) Karl-Franzens University in Graz (Austria) (from /02/2007 to / 03/2008) and Rowett Research Institute Centre, in Aberdeen (UK) (from 14/07/2008 to 14/09/2008).

Development of precise cellular *in vitro* assay to evaluate the antiinflammatory and vasodilatation properties of different bioactive natural compounds. Laia PONS LLECHA, European Doctorate. Training in Bolonya (Italia) (from 1/10/2008 to 19/12/2008)

From nuclear factors to protein secretion: antiinflamatory and improve endothelial function properties of different bioactive natural compounds. Úrsula CATALÀN SANTOS. European Doctorate. Training in COSPI-3 in the third year of our Erasmus Intensive Programme “Combating Obesity: Strategies for Prevention and Intervention”.

The Erasmus Socrates Mobility Programme Dra. Begoña MANUEL-KEENOY. The AMRU (Antwerp Metabolic Research Unit), University of Antwerp (Amberes; Bèlgica). Title Course: ASSESSMENT OF ANALYTICAL QUALITY FOR BIOMEDICAL RESEARCH; in Reus (Spain)

1. From 5^Th^ May to 9^th^ May 2008) and

2. From 20^th^ April to 26^th^ April 2009.

The Erasmus Intensive Programme is co-financed by a grant from the European Commission, Education, Audiovisual & Culture Executive Agency, under the contract 28563-IC-1-2005-1-AT-ERASMUS-IPUC-1.

2007, 2008 and 2009

Director:

Brigitte M. Winklhofer-Roob, M.D.

Human Nutrition & Metabolism Research and Training Center

Institute of Molecular Biosciences

Karl-Franzens University

Universitätsplatz 2

8010 Graz, Austria

The European Program, Leonardo da Vinci: Master training students Université d’Auvergne (Clermont-Ferrand, France). Marie COISNON, Training from may 15 to july 31 2007. Master training students. Université de Rennes II; Mélanie BESSON, From 29th Juny 2008-31th August 2008.
