# Supplementary figures and images for: Impact of Virgin Olive Oil and Phenol-Enriched Virgin Olive Oils on the HDL Proteome in Hypercholesterolemic Subjects: A Double Blind, Randomized, Controlled, Cross-Over Clinical Trial (VOHF Study)
Source: PLoS One. 2015 Jun 10;10(6):e0129160. doi: 10.1371/journal.pone.0129160 (PMC4465699; doi:10.1371/journal.pone.0129160)

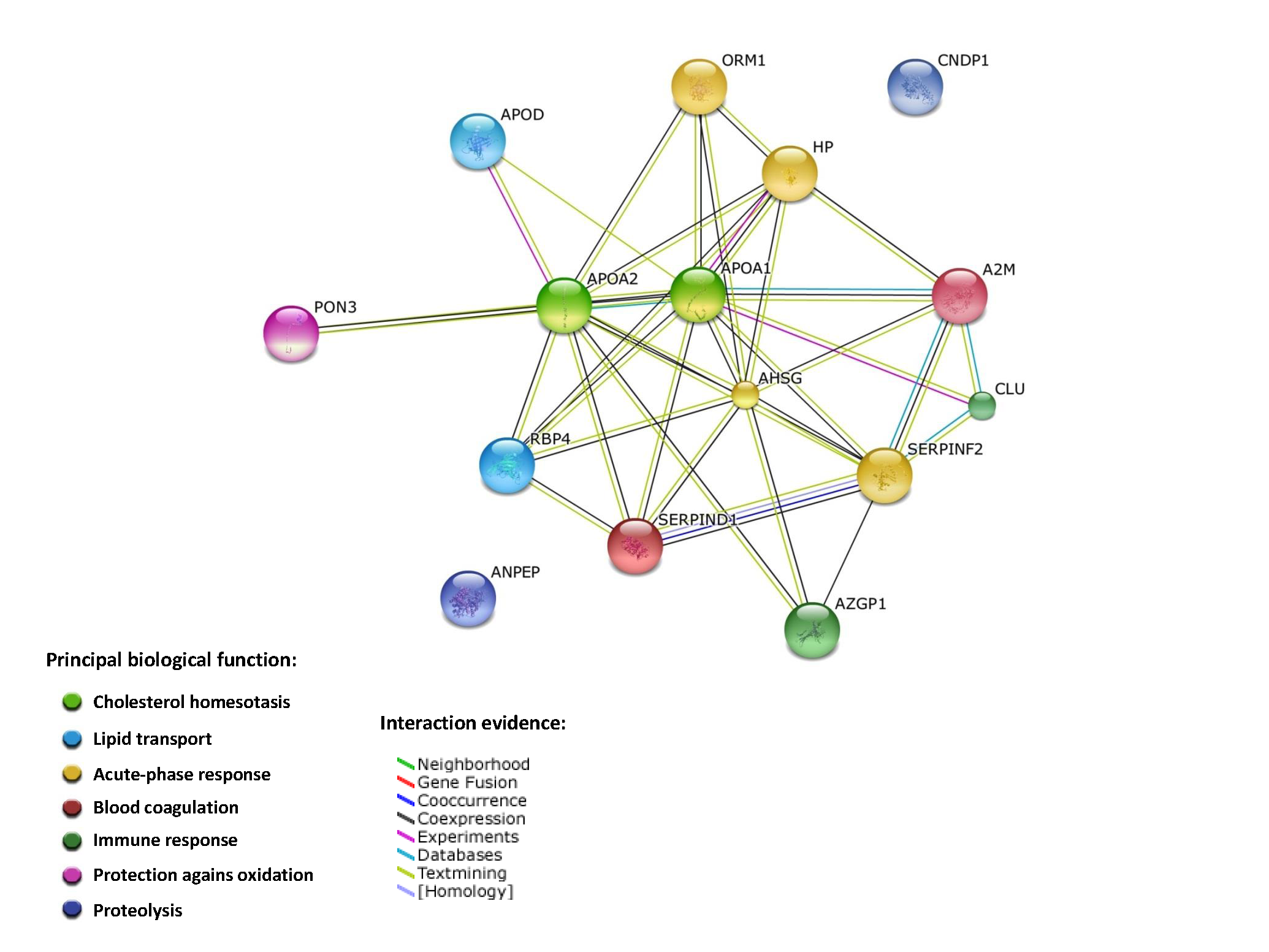

Supplement: S1 Fig — Proteins were presented with their gene encode symbol. Nodes represent genes encoding interacting proteins and the lines between them represent known and predicted interactions. (TIFF) [file pone.0129160.s003.tiff]
