# Supplementary material for: Impact of Virgin Olive Oil and Phenol-Enriched Virgin Olive Oils on the HDL Proteome in Hypercholesterolemic Subjects: A Double Blind, Randomized, Controlled, Cross-Over Clinical Trial (VOHF Study)
Source: PLoS One. 2015 Jun 10;10(6):e0129160. doi: 10.1371/journal.pone.0129160 (PMC4465699; doi:10.1371/journal.pone.0129160)
Supplement: S1 File — (DOCX) [file pone.0129160.s004.docx]

**SUPPORTING INFORMATION S1.**

**SUPPLEMENTAL METHODS**

**Proteomic sample preparation and analysis**

***Pooling***

The 123 isolated HDL samples were divided into ten pools taking into consideration the randomized sequence detailed in **Figure 2** and the visit number. Pool 1 includes the HDL fraction from visit 1 of all participants (n=33) in order to normalize the data obtained between the three groups (**Figure 3**). Pool 1 includes the HDL fraction from visit 1 of all participants (n=33) in order to normalize the data obtained among the three administration sequences. Pool 2 includes the HDL fractions from visit 3 of participants that followed the administration sequence 1 (n=11). Pool 3 includes the HDL fraction from visit 3 of volunteers that have followed the administration sequence 2 (n=11). Pool 4 includes the HDL fraction from visit 3 of volunteers that followed the administration sequence 3 (n=11). Pool 5 includes the HDL fraction from visit 5 of participants that have followed the administration sequence 1 (n=9). Pool 6 includes the HDL fraction from visit 5 of volunteers that followed the administration sequence 2 (n=11). Pool 7 includes the HDL fraction from visit 5 of volunteers that followed the administration sequence 3 (n=10). Pool 8 includes the HDL fraction from visit 7 of participants that followed the administration sequence 1 (n=8). Pool 9 includes the HDL fraction from visit 7 of volunteers that followed the administration sequence 2 (n=9). Pool 10 includes the HDL fraction from visit 7 of participants that followed the administration sequence 3 (n=10; **Figure 3**).

Protein pools were dialyzed using Amicon Ultra-4 10K filters (Millipore, Co. Cork, IRL) at 4 000 x*g* for 20 minutes at 4 ºC.

***HDL delipidation and protein precipitation***

After concentration, pools were delipidated and precipitated. Briefly, concentrated HDL samples were dripped over 3 mL of methanol (Sigma-Aldrich, Tres Cantos, Spain) and then 7 mL of diethyl ether (-20°C; Sigma-Aldrich, Tres Cantos, Spain) were added. Samples were incubated for 10 minutes on ice, centrifuged at 1 000 rpm for 5 minutes at 4 °C, and supernatants were aspired. The step was repeated with 10 mL of diethyl ether (-20°C) and finally pelleted samples were suspended into 200 µL of sodium dodecyl sulfate- dithiothreitol (SDS-DTT) solution (62.5 mM Tris-HCl, 2%, SDS, 40 mM DTT) and stored at -80ºC. Samples were precipitated with trichloroacetic acid (TCA) (Sigma-Aldrich, Tres Cantos, Spain) and acetone (AppliChem GmbH , Darmstadt, Germany), to concentrate and purify them. The pellet was resuspended in dissolution buffer (0.5 M triethylammonium bicarbonate, pH 8.5 and 0.1% SDS). Total protein concentration was calculated by Bradford assay (Sigma, St Louis, MO, USA).

***In-solution protein digestion***

50 µg of each sample was reduced for 1 h at 60 ºC with 5 mM tris-(2-carboxyethyl)phosphine (TCEP) (Sigma-Aldrich, Tres Cantos, Spain) and alkylated for 30 min at room temperature in the dark with 4mM iodoacetamide (Sigma-Aldrich, Tres Cantos, Spain). Sequencing grade trypsin (Promega Corporation, Madison, USA) was added in a 1:100 (w/w) ratio and the incubation was carried out overnight at 48 °C.

***iTRAQ labeling***

Samples were labelled following the manufacturer's instructions of iTRAQ 4-plex (Applied Biosystems, Foster City, CA, USA), using 3 separate 4-plex iTRAQ kits, one for each randomized sequence (**Figure 3)**. Control (pool 1) was labeled with 114 tag, and the rest of the samples were labelled by alternating 115, 116, and 117 tags to avoid bias. The excess of iTRAQ reagents was removed using a strong cation exchange (SCX)-cartridge (Strata, Phenomenex, Torrence, CA, USA). Peptides were eluted with a volatile buffer containing 5% ammonia and 30% methanol and subsequently dried in SpeedVac system prior to OffGel electrophoresis (OGE) fractionation.

***OffGel fractionation***

The resulting labeled peptides were separated according to their isoelectric point using 24 cm IPG strips (pH 3-10) on an Agilent 3100 OFFGEL fractionator, the separated components were then recovered in liquid fractions. The three sample sequences (three sets of iTRAQ labelling) we processed in parallel, diluting the dried mixtures in 1.8 ml of the focusing buffer containing only 5% (v/v) of glycerol contrary to the supplier’s protocol. IPG strips (24cm, pH 3-10) were rehydrated by adding 40 μl of peptide IPG strip rehydration solution per well for 15 minutes. Then, the dried peptide mixtures samples were dissolved directly in the peptide OFFGEL stock solution, pH 3-10 and 150 μl of sample were loaded in each well. Peptide focusing was performed until it reached 50 kVh with a maximum voltage of 8 000 V and maximum current of 50 μA. After focusing, the 24 liquid fractions were carefully recovered and well rinsed twice with 150 μl of 0.1% Formic Acid (FA) for 15 minutes, to recover the maximum sample solution for each well. 3x24 peptide fractions were finally obtained.

All fractions were checked by MALDI-TOF, those with higher mass intensities were kept alone whilst fractions with similar or lower intensities were combined, resulting 10 different fractions for each set of iTRAQ labelling. Samples were desalted and purified using Sep-Pack 100 mg C18 cartridge (Waters, Elstree, UK). The eluent was concentrated to dryness and reconstituted in 50 µL 0.1% FA.

***Nano-LC separation and MALDI MS/MS***

A 15 μl-aliquot of each fraction was analyzed by LC–MALDI-TOF/TOF. An Easy-nLC system (Proxeon, Thermo Scientific) coupled to a Proteinner-fc fraction collector (Bruker) was used for the nano-LC with a C18 pre-column (EASY-Column, 2 cm, ID100 µm, 5 µm, C18-A1) and a C18 analytical column (EASY-Column, 10cm, ID75µm, 3µm, C18-A2). Mobile phases were water (A) and ACN (B), both containing 0.1% (v/v) trifluoroacetic acid (TFA). Peptides were separated over 35 min at a flow rate of 0.3 μL/min as follows: 0−60% solvent B within 10 min; 10 min 100% solvent B; and held for 15 min 2% solvent B before ramping back down to the initial solvent conditions. During gradient, 16’’ fractions were collected and directly deposited on a Maldi Target plate PACII 384, pre-spotted with α-cyano-4-hydroxycinnamic acid matrix. MALDI plates were rinsed with 10 mM ammonium phosphate monobasic buffer (Sigma-Aldrich, Tres Cantos, Spain) and immediately analyzed on a MALDI-TOF/TOF UltrafleXtrem (Bruker Daltonics, Bremen, Germany) instrument. Automated data acquisition was done using Flex Control v 3.4 and Warp-LC v 1.3 softwares ( Bruker) , and was constituted to a full scan (m/z 700-5000) in positive reflector ion mode using 2 000 laser shots for every MS spectra. The twenty most abundant peptide precursor ions, with a signal-to-noise ratio greater than or equal to 10, were selected for MS/MS analysis with LIFT detection mode using 4 000 laser shots.

***Data analysis (MALDI)***

The raw data files for protein identification were processed using FlexAnalysis v 3.4 via ProteinScape v 3 softwares (Bruker). Searches were performed with Mascot v 2.4.0 (Matrix Science, London, UK), against the SwisProt (released on 03/2013) human database. The following search parameters were selected: Homo sapiens for organism, trypsin as the digestion enzyme, with up to 2 missed cleavages allowed, cysteine carbamidomethylation as a fixed modification, whilst oxidation of methionine was set as variable. Tolerance settings were 50 ppm for full scan and 0.9 for MS/MS. Protein identifications were accepted with a minimum peptide Mowse score of 10 and a minimum Protein Mascot Score of 20.

***Nano-LC separation and ORBITRAP MS/MS***

Samples were analyzed on an Orbitrap Velos PRO instrument (Thermo Fisher Scientific, Bremen) connected to an Easy-nLC system (Proxeon, Thermo Scientific) with the same configuration columns as the nLC for MALDI, but coupled to a nanoelectrospray source with a stainless steel emitter. Solvent A consisted of 0.1 % AF in deionized water (Milli-Q, Millipore), and solvent B consisted of 0.1 % AF in 100% acetonitrile (ACN). The flow rate was 0.3 μL/min with a 130 min long separation gradient running from 5% to 100% B. The mass spectrometry detection constituted a full scan (m/z 350–2 000) with Orbitrap detection at resolution R=30,000 (at m/z 400) followed by up to ten data-dependent acquisition MS/MS scans with Orbitrap detection of the most intense ions. The signal threshold for triggering an MS/MS event was set to 10 000 counts. The low mass cutoff was set to 100 m/z. Charge state screening was enabled, and precursors with unknown charge state or a charge state of 1 were excluded. Dynamic exclusion of 30 s was used with an activation time of 0.1 s. For efficient fragmentation and detection of iTRAQ reporter ions, HCD normalized collision energy of 45 was used since reported optimization experiments showed that it gave the highest number of identified peptides with iTRAQ signal.

***Data analyses (ORBITRAP)***

Peak lists were searched against Swissprot (released on 03/2013), taxonomy filter was set to Homo sapiens using Mascot software version 2.4.04 (Matrix Science, UK). Proteome Discover v 1.4 was used for protein identification and iTRAQ reporter quantification. Trypsin was chosen as cleavage specificity with a maximum number of three missed cleavages allowed. Carbamidomethylation (C) was set as a fixed modification whereas those originating from iTRAQ protocol (iTRAQ4Plex on the N-terminal residue, iTRAQ4Plex on tyrosine (Y), iTRAQ4Plex on lysine (K)) and oxidation (M) were used as variable modifications. The searches were performed using a peptide tolerance of 7 ppm and a product ion tolerance of 0.02 Da (Orbitrap readout). For further filtering the decoy search options was enabled and only unique peptides were accepted. The resulting data files were exported and filtered for <1% false discovery rate at peptide. Hence there is no fixed Mascot score cutoff because peptide matches are accepted until a FDR rate of 1% is reached. However, only PSMs with Mascot scores >20 were accepted to ensure that only high quality data were employed for this study.

The files generated with Maldi and Orbitrap MS/MS were combined in order to identify the HDL-associated proteins. The final identified proteins were required to present more than one peptide-spectrum match (PSM) or have a confidence score at least >30% and a coverage >10%.

***Quantification and statistics (ORBITRAP)***

Samples analyzed by nLC-ORBIRAP-ESI MS/MS were used for the quantitative analyzes. In the quantitative calculations, only protein isoforms with iTRAQ values in at least two of the analyses were included. For each protein, the relative expression levels were calculated as a ratio between the sample and the 114 labeling controls. Biological replicates from the three iTRAQ sequences were combined in an Excel document where the mean of the ratios of each protein were calculated. The fold changes for differential expression have been calculated as reported in Mangé et al., 2012. The significant cutoff threshold of fold change was determined using two equal amounts of tryptic digested protein samples labeled with iTRAQ reagent 114 and 115 respectively, and analyzed with nanoLC/MS/MS. The standard deviaton (SD) based on the ratios of 80 identified proteins was 0.125. A cutoff of 1± 2 SD (1.3 and 0.8 for the significant up-regulated and down-regulated proteins, respectively) was determined to be significant (p < 0.05). Proteins identified as differentially expressed were those with a differential expression of at least 0.8-fold change, or 1.3-fold change relative to the baseline. In addition, a cut-off inferior to 0.5 or superior to 1.5 was applied to establish the more relevant protein expression changes observed after each intervention, which were defined as stronger effects.
