# Supplementary material for: Impact of Virgin Olive Oil and Phenol-Enriched Virgin Olive Oils on the HDL Proteome in Hypercholesterolemic Subjects: A Double Blind, Randomized, Controlled, Cross-Over Clinical Trial (VOHF Study)
Source: PLoS One. 2015 Jun 10;10(6):e0129160. doi: 10.1371/journal.pone.0129160 (PMC4465699; doi:10.1371/journal.pone.0129160)
Supplement: S1 Table — (DOCX) [file pone.0129160.s005.docx]

**Table S1.** Concentration of the different phenolic metabolites present in the HDL pools.

|  | **BASAL** | **VOO**  **post-intervention** | **FVOO**  **post-intervention** | **FVOOT**  **post-intervention** |
| --- | --- | --- | --- | --- |
| **Olive phenolics (nanomols/L)** |  |  |  |  |
| sulfHT | 0,00 | 0,60 | 3,99 | 14,26 |
| sulfHTAc | 0,00 | 12,72 | 37,44 | 23,89 |
| sulfHValc | 1,12 | 0,00 | 29,82 | 11,31 |
| sulfHVac | 0,88 | 0,08 | 21,67 | 37,32 |
| **Thyme Phenolics (nanomols/L)** |  |  |  |  |
| sulfTHY | 33,39 | 15,12 | 42,84 | 580,75 |
| sulfCA | 0,00 | 3,26 | 4,96 | 145,12 |
| sulfHPPA | 10,66 | 9,88 | 32,22 | 326,85 |
|  |  |  |  |  |

sulfHT, hydroxytyrosol sulfate; sulfHTAc, hydroxytyrosol acetate sulfate; sulfHValc, homovanillic alcohol sulfate; sulfHVac, homovanillic acid sulfate; sulfTHY, thymol sulfate; sulfCA, caffeic acid sulfate; sulfHPPA, hydroxyphenylpropionic acid sulfate.
