# Supplementary material for: Impact of Virgin Olive Oil and Phenol-Enriched Virgin Olive Oils on the HDL Proteome in Hypercholesterolemic Subjects: A Double Blind, Randomized, Controlled, Cross-Over Clinical Trial (VOHF Study)
Source: PLoS One. 2015 Jun 10;10(6):e0129160. doi: 10.1371/journal.pone.0129160 (PMC4465699; doi:10.1371/journal.pone.0129160)
Supplement: S2 Table — Phenolic compounds, fat soluble micronutrients and fatty acids daily intake through 25 mL of VOO, FVOO and FVOOT. (DOCX) [file pone.0129160.s006.docx]

**Supporting Information Table S2.** Virgin olive oils composition. Phenolic compounds, fat soluble micronutrients and fatty acids daily intake through 25 mL of VOO, FVOO and FVOOT.

|  | **VOO** | | | **FVOO** | | **FVOOT** | |
| --- | --- | --- | --- | --- | --- | --- | --- |
| **PHENOLIC COMPOUNDS (mg/25 mL/day)** | | |  |  |  |  |  |
| hydroxytyrosol | | 0,01 | ± 0,00 | 0,21 | ± 0,02 | 0,12 | ± 0,00 |
| 3,4-DHPEA-AC | | n.d. |  | 0,84 | ± 0,06 | 0,39 | ± 0,04 |
| 3,4-DHPEA-EDA | | 0,04 | ± 0,00 | 6,73 | ± 0,37 | 3,43 | ± 0,29 |
| 3,4-DHPEA-EA | | 0,26 | ± 0,04 | 0,71 | ± 0,06 | 0,36 | ± 0,03 |
| ***Total hydroxytyrosol derivates*** | | ***0,30*** |  | ***8,49*** |  | ***4,30*** |  |
| p-hydroxybenzoic acid | | n.d. |  | 0,02 | ± 0,00 | 0,06 | ± 0,00 |
| vanillic acid | | n.d. |  | 0,07 | ± 0,00 | 0,13 | ± 0,01 |
| caffeic acid | | n.d. |  | 0,00 | ± 0,00 | 0,06 | ± 0,00 |
| rosmarinic acid | | n.d. |  | n.d. |  | 0,41 | ± 0,03 |
| ***Total phenolic acids*** | | ***-*** |  | ***0,09*** |  | ***0,65*** |  |
| thymol | | n.d. |  | n.d. |  | 0,64 | ± 0,05 |
| carvacrol | | n.d. |  | n.d. |  | 0,23 | ± 0,02 |
| ***Total monoterpenes*** | | ***-*** |  | ***-*** |  | ***0,86*** |  |
| luteolin | | 0,04 | ± 0,00 | 0,18 | ± 0,02 | 0,21 | ± 0,02 |
| apigenin | | 0,02 | ± 0,00 | 0,06 | ± 0,00 | 0,10 | ± 0,00 |
| naringenin | | n.d. |  | n.d. |  | 0,20 | ± 0,02 |
| eriodictyol | | n.d. |  | n.d. |  | 0,17 | ± 0,01 |
| thymusin | | n.d. |  | n.d. |  | 1,22 | ± 0,09 |
| xanthomicrol | | n.d. |  | n.d. |  | 0,53 | ± 0,06 |
| 7-methylsudachitin | | n.d. |  | n.d. |  | 0,53 | ± 0,09 |
| ***Total flavonoids*** | | ***0,06*** |  | ***0,23*** |  | ***2,95*** |  |
| pinoresinol | | 0,05 | ± 0,00 | 0,12 | ± 0,00 | 0,10 | ± 0,05 |
| acetoxipinoresinol | | 2,47 | ± 0,19 | 3,66 | ± 0,31 | 3,24 | ± 0,28 |
| ***Total lignans*** | | ***2,52*** |  | ***3,78*** |  | ***3,34*** |  |
| **FAT SOLUBLE MICRONUTRIENTS (mg/25 mL/day)** | | | |  |  |  |  |
| α-tocopherol | | 3,27 | ± 0,01 | 3,40 | ± 0,02 | 3,44 | ± 0,01 |
| lutein | | 0,05 | ± 0,00 | 0,06 | ± 0,00 | 0,07 | ± 0,00 |
| β-cryptoxanthin | | 0,02 | ± 0,00 | 0,03 | ± 0,00 | 0,02 | ± 0,00 |
| β-carotene | | 0,01 | ± 0,00 | 0,02 | ± 0,00 | 0,02 | ± 0,00 |
|  | |  |  |  |  |  |  |
| **FATTY ACIDS (relative area %)** | | | |  |  |  |  |
| Palmitic acid | | 11,21 |  | 11,20 |  | 11,21 |  |
| Stearic acid | | 1,92 |  | 1,92 |  | 1,92 |  |
| Araquidic acid | | 0,36 |  | 0,36 |  | 0,36 |  |
| Behenic acid | | 0,11 |  | 0,11 |  | 0,11 |  |
| ***Total saturated*** | | ***13,75*** |  | ***13,74*** |  | ***13,75*** |  |
| Palmitoleic acid | | 0,70 |  | 0,70 |  | 0,69 |  |
| Oleic acid | | 76,74 |  | 76,83 |  | 76,75 |  |
| Gadoleic acid | | 0,27 |  | 0,27 |  | 0,27 |  |
| ***Total monounsaturated*** | | ***77,71*** |  | ***77,80*** |  | ***77,72*** |  |
| Linoleic acid | | 7,43 |  | 7,36 |  | 7,43 |  |
| Timnodonic acid | | 0,36 |  | 0,36 |  | 0,35 |  |
| Linolenic acid | | 0,43 |  | 0,43 |  | 0,43 |  |
| ***Total polyunsaturated*** | | ***8,22*** |  | ***8,15*** |  | ***8,22*** |  |

Values expressed as mean ± standard deviation (SD). 3,4-DHPEA-AC, 4-**(**acetoxyethyl**)**-1,2-dihydroxybenzene; 3,4-DHPEA-EDA, dialdehydic form of elenolic acid linked to hydroxytyrosol; 3,4-DHPEA-EA, oleuropein aglycone
