# Supplementary material for: Impact of Virgin Olive Oil and Phenol-Enriched Virgin Olive Oils on the HDL Proteome in Hypercholesterolemic Subjects: A Double Blind, Randomized, Controlled, Cross-Over Clinical Trial (VOHF Study)
Source: PLoS One. 2015 Jun 10;10(6):e0129160. doi: 10.1371/journal.pone.0129160 (PMC4465699; doi:10.1371/journal.pone.0129160)
Supplement: S3 Table — (DOCX) [file pone.0129160.s007.docx]

**Table S3.** Biochemical characterization of HDL segregated according to VOO intervention sequence.

|  | VOO | |  | FVOO | |  | FVOOT | |  |
| --- | --- | --- | --- | --- | --- | --- | --- | --- | --- |
|  | (n=30) | |  | (n=30) | |  | (n=30) | |  |
| HDL FRACTION | Basal levels | Post intervention levels | *p* | Basal levels | Post intervention levels | *p* | Basal levels | Post intervention levels | *p* |
|  | (Mean ± SD) | (Mean ± SD) |  | (Mean ± SD) | (Mean ± SD) |  | (Mean ± SD) | (Mean ± SD) |  |
| Total Cholesterol (mg/dL) | 53.29 (13.29) | 56.30 (15.04) | 0.033 | 54.92 (14.56) | 58.19 (15.02) | 0.037 | 53.38 (13.37) | 57.78 (16.03) | 0.005 |
| Free Cholesterol (mg/dL) | 17.66 (5.46) | 18.53 (6.22) | 0.123 | 18.45 (5.95) | 19.80 (6.01) | 0.030 | 17.79 (5.55) | 18.96 (6.04) | 0.030 |
| Esterified Cholesterol (mg/dL) | 35.59 (8.13) | 38.83 (10.40) | 0.003 | 35.62 (8.16) | 37.77 (9.29) | 0.022 | 36.46 (8.89) | 38.38 (9.34) | 0.059 |
| Phospholipids (mg/dL) | 99.86 (23.17) | 103.86 (22.06) | 0.146 | 102.25 (23.59) | 109.31 (22.29) | 0.021 | 99.42 (22.84) | 104.85 (21.82) | 0.036 |
| Triglycerides (mg/dL) | 9.32 (7.27) | 8.05 (12.60)^*^ | 0.819 | 8.91 (7.46) | 8.94 (6.91) | 0.980 | 9.13 (7.30) | 5.64 (2.71)† | 0.147 |
| Apo A-I (mg/dL) | 114.92 (31.63) | 117.60 (30.00)^*^ | 0.027 | 113.92 (29.41) | 123.20 (25.33) | 0.021 | 113.16 (29.61) | 124.27 (27.31) | 0.006 |
| Apo A-II (mg/dL) | 25.79 (2.81) | 26.83 (2.72) | 0.029 | 25.86 (2.94) | 26.99 (3.16) | 0.019 | 25.67 (2.85) | 27.02 (3.43) | 0.004 |
| Total protein (mg/dL) | 203.60 (38.88) | 213.78 (32.95) | 0.073 | 204.56 (37.33) | 215.44 (33.01) | 0.045 | 202.43 (37.74) | 214.96 (39.31) | 0.037 |
| Total mass (mg/dL) | 366.07 (72.24) | 382.88 (67.84) | 0.050 | 370.65 (71.55) | 391.88 (65.32) | 0.014 | 364.37 (70.66) | 385.34 (73.91) | 0.016 |
| Average chemical composition (%) Protein/PL/TC/TG | 55.8/27.2/14.5/2.5 | 56.1/27/14.6/2.4 | n.s | 55.4/27.5/14.7/2.4 | 55.2/27.8/14.7/2.3 | n.s | 55.7/27.2/14.6/2.5 | 56/27.2/14.8/2 | n.s |
| PLASMA |  |  |  |  |  |  |  |  |  |
|  |  |  |  |  |  |  |  |  |  |
| Apo A-I (mg/dL) | 136.00 (39.50)* | 144.50 (26.50)* | 0.041 | 138.29 (1.36)† | 150.01 (1.27)† | 0.056 | 140.00 (42.50)* | 147.96 (1.26)† | 0.030 |
| HDL-c (mg/dL) | 1.49 (0.28) | 1.56 (0.35) | 0.056 | 1.56 (0.31) | 1.67 (0.40) | 0.024 | 1.52 (0.30) | 1.59 (0.36) | 0.074 |

* Data are presented as median ± (IQR = interquartile range); † data are presented as geometric mean (antilog SD). Total mass of HDL particle was calculated as the sum of the masses of HDL total cholesterol, triglycerides, phospholipids and total protein. A paired Student’s t-test was employed for the comparison of paired and normally distributed variables. Wilcoxon signed-rank test was used for the comparison of paired and non-normally distributed variables. *p*: two-tailed test of significance between basal levels and post intervention levels. *n.s:* non-significance in either of the chemical composition parameters (%) between basal levels and post intervention levels. There were no significant differences between the three VOO interventions either basal levels or post intervention levels.
