# Supplementary material for: Impact of Virgin Olive Oil and Phenol-Enriched Virgin Olive Oils on the HDL Proteome in Hypercholesterolemic Subjects: A Double Blind, Randomized, Controlled, Cross-Over Clinical Trial (VOHF Study)
Source: PLoS One. 2015 Jun 10;10(6):e0129160. doi: 10.1371/journal.pone.0129160 (PMC4465699; doi:10.1371/journal.pone.0129160)
Supplement: S4 Table — (DOCX) [file pone.0129160.s008.docx]

**Supporting Information Table S4.** HDL-associated proteins identified by MALDI and ORBITRAP MS techniques.

| **UNIPROT ACCESSION NUMBER** | **GENE SYMBOL** | **ENTRY NAME** | **PROTEIN NAME** | **SEQUENCE COVERAGE (%)** | **NUMBER OF UNIQUE PEPTIDES IDENTIFIED** | **NUMBER OF PSMs IDENTIFIED** | **MASCOT SCORE** |
| --- | --- | --- | --- | --- | --- | --- | --- |
| P60709 | ACTB | ACTB_HUMAN | Actin. cytoplasmic 1 | 17.87 | 4 | 5 | 56 |
| Q9HDC9 | APMAP | APMAP_HUMAN | Adipocyte plasma membrane-associated protein | 6.97 | 3 | 6 | 54.4 |
| P43652 | AFM | AFAM_HUMAN | Afamin | 3.01 | 2 | 8 | 98.2 |
| P02763 | ORM1 | A1AG1_HUMAN | Alpha-1-acid glycoprotein 1 | 18.91 | 3 | 12 | 105.1 |
| P19652 | ORM2 | A1AG2_HUMAN | Alpha-1-acid glycoprotein 2 | 12.94 | 2 | 6 | 64.4 |
| P01011 | SERPINA3 | AACT_HUMAN | Alpha-1-antichymotrypsin | 16.08 | 7 | 36 | 388.9 |
| P01009 | SERPINA1 | A1AT_HUMAN | Alpha-1-antitrypsin | 36.84 | 13 | 133 | 1499.3 |
| P04217 | A1BG | A1BG_HUMAN | Alpha-1B-glycoprotein | 12.53 | 4 | 11 | 120 |
| P08697 | SERPINF2 | A2AP_HUMAN | Alpha-2-antiplasmin | 23.63 | 6 | 18 | 166 |
| P02765 | AHSG | FETUA_HUMAN | Alpha-2-HS-glycoprotein | 21.8 | 9 | 76 | 704.4 |
| P01023 | A2M | A2MG_HUMAN | Alpha-2-macroglobulin | 0.75 | 1 | 1 | 12.6 |
| P15144 | ANPEP | AMPN_HUMAN | Aminopeptidase N | 2.17 | 2 | 4 | 47.4 |
| P01019 | AGT | ANGT_HUMAN | Angiotensinogen | 21.24 | 8 | 38 | 422.3 |
| P01008 | SERPINC1 | ANT3_HUMAN | Antithrombin-III | 17.03 | 5 | 10 | 163.1 |
| P02647 | APOA1 | APOA1_HUMAN | Apolipoprotein A-I | 59.55 | 24 | 455 | 3876.5 |
| P02652 | APOA2 | APOA2_HUMAN | Apolipoprotein A-II | 67 | 7 | 50 | 410.7 |
| P06727 | APOA4 | APOA4_HUMAN | Apolipoprotein A-IV | 33.59 | 14 | 27 | 266.7 |
| Q6Q788 | APOA5 | APOA5_HUMAN | Apolipoprotein A-V | 16.9 | 2 | - | 64.3 |
| P04114 | APOB | APOB_HUMAN | Apolipoprotein B-100 | 36.03 | 157 | 794 | 7600.1 |
| P02654 | APOC1 | APOC1_HUMAN | Apolipoprotein C-I | 13.25 | 1 | 1 | 12.6 |
| P02655 | APOC2 | APOC2_HUMAN | Apolipoprotein C-II | 49.5 | 4 | 21 | 217.7 |
| P02656 | APOC3 | APOC3_HUMAN | Apolipoprotein C-III | 37.37 | 4 | 153 | 1652.9 |
| P55056 | APOC4 | APOC4_HUMAN | Apolipoprotein C-IV | 50.4 | 6 | - | 137.9 |
| P05090 | APOD | APOD_HUMAN | Apolipoprotein D | 23.81 | 5 | 69 | 613.3 |
| P02649 | APOE | APOE_HUMAN | Apolipoprotein E | 40.38 | 12 | 62 | 617 |
| Q13790 | APOF | APOF_HUMAN | Apolipoprotein F | 7.36 | 2 | 2 | 20.7 |
| O14791 | APOL1 | APOL1_HUMAN | Apolipoprotein L1 | 14.82 | 8 | 57 | 564 |
| Q9BQE5 | APOL2 | APOL2_HUMAN | Apolipoprotein L2 | 2.08 | 1 | 2 | 20.1 |
| O95445 | APOM | APOM_HUMAN | Apolipoprotein M | 54.79 | 6 | 75 | 536.8 |
| P08519 | LPA | APOA_HUMAN | Apolipoprotein(a) | 44.13 | 32 | 188 | 1624.6 |
| P02730 | SLC4A1 | B3AT_HUMAN | Band 3 anion transport protein | 1.43 | 1 | 2 | 28 |
| P02749 | APOH | APOH_HUMAN | Beta-2-glycoprotein 1 | 17.39 | 4 | 10 | 79.2 |
| Q96KN2 | CNDP1 | CNDP1_HUMAN | Beta-Ala-His dipeptidase | 12.23 | 4 | 9 | 92.6 |
| Q8TDL5 | BPIFB1 | BPIB1_HUMAN | BPI fold-containing family B member 1 | 15.5 | 5 | 20 | 196.6 |
| P00915 | CA1 | CAH1_HUMAN | Carbonic anhydrase 1 | 7.28 | 1 | 1 | 14.9 |
| P23280 | CA6 | CAH6_HUMAN | Carbonic anhydrase 6 | 7.14 | 1 | 1 | 15.3 |
| Q9NNX6 | CD209 | CD209_HUMAN | CD209 antigen | 13.37 | 1 | 1 | 11.2 |
| P16070 | CD44 | CD44_HUMAN | CD44 antigen | 2.7 | 2 | 2 | 23.2 |
| O43866 | CD5L | CD5L_HUMAN | CD5 antigen-like | 4.03 | 1 | 2 | 33.3 |
| P11597 | CETP | CETP_HUMAN | Cholesteryl ester transfer protein | 19.27 | 5 | 8 | 85.1 |
| P10909 | CLU | CLUS_HUMAN | Clusterin | 22.27 | 10 | 43 | 372.6 |
| P00736 | C1R | C1R_HUMAN | Complement C1r subcomponent | 3.85 | 2 | - | 66.1 |
| P06681 | C2 | CO2_HUMAN | Complement C2 | 4.8 | 2 | - | 67.8 |
| P01024 | C3 | CO3_HUMAN | Complement C3 | 26.64 | 32 | 81 | 796.6 |
| P0C0L4 | C4A | CO4A_HUMAN | Complement C4-A | 15.14 | 15 | 41 | 394.1 |
| P0C0L5 | C4B | CO4B_HUMAN | Complement C4-B | 46.6 | 73 | - | 2419.4 |
| P02748 | C9 | CO9_HUMAN | Complement component C9 | 19.8 | 9 | - | 332.8 |
| P00746 | CFD | CFAD_HUMAN | Complement factor D | 26.88 | 3 | 6 | 79.9 |
| P08603 | CFH | CFAH_HUMAN | Complement factor H | 10.3 | 5 | - | 147.7 |
| P01034 | CST3 | CYTC_HUMAN | Cystatin-C | 19.18 | 2 | 15 | 167.3 |
| P81605 | DCD | DCD_HUMAN | Dermcidin | 7.27 | 1 | 1 | 16.2 |
| Q08554 | DSC1 | DSC1_HUMAN | Desmocollin-1 | 1.45 | 1 | 1 | 13.7 |
| P27105 | STOM | STOM_HUMAN | Erythrocyte band 7 integral membrane protein | 3.47 | 1 | 2 | 25.3 |
| P02671 | FGA | FIBA_HUMAN | Fibrinogen alpha chain | 17.44 | 13 | 76 | 676.8 |
| P02675 | FGB | FIBB_HUMAN | Fibrinogen beta chain | 36.7 | 12 | - | 389.6 |
| P02679 | FGG | FIBG_HUMAN | Fibrinogen gamma chain | 3.31 | 1 | 3 | 45.4 |
| P02751 | FN1 | FINC_HUMAN | Fibronectin | 2.3 | 2 | - | 76.2 |
| P21333 | FLNA | FLNA_HUMAN | Filamin-A | 0.34 | 1 | 1 | 14.9 |
| P06396 | GSN | GELS_HUMAN | Gelsolin | 11.51 | 5 | 10 | 122.2 |
| P00738 | HP | HPT_HUMAN | Haptoglobin | 23.89 | 2 | 52 | 498.5 |
| P00739 | HPR | HPTR_HUMAN | Haptoglobin-related protein | 43.1 | 5 | 84 | 770.3 |
| P69905 | HBA1 | HBA_HUMAN | Hemoglobin subunit alpha | 10.56 | 1 | 1 | 12.4 |
| P68871 | HBB | HBB_HUMAN | Hemoglobin subunit beta | 15.65 | 2 | 2 | 21 |
| P02790 | HPX | HEMO_HUMAN | Hemopexin | 9.74 | 2 | 6 | 82.5 |
| P05546 | SERPIND1 | HEP2_HUMAN | Heparin cofactor 2 | 5.41 | 2 | 3 | 40.1 |
| P04196 | HRG | HRG_HUMAN | Histidine-rich glycoprotein | 5.71 | 2 | 2 | 20.3 |
| P10412 | HIST1H1E | H14_HUMAN | Histone H1.4 | 7.31 | 1 | 1 | 16.4 |
| P04439 | HLAA | 1A03_HUMAN | HLA class I histocompatibility antigen. A-3 alpha chain | 13.7 | 2 | 15 | 147.9 |
| P30464 | HLAB | 1B15_HUMAN | HLA class I histocompatibility antigen. B-15 alpha chain | 10.5 | 1 | 10 | 109.8 |
| P01876 | IGHA1 | IGHA1_HUMAN | Ig alpha-1 chain C region | 36.83 | 9 | 31 | 290.8 |
| P01857 | IGHG1 | IGHG1_HUMAN | Ig gamma-1 chain C region | 20.3 | 3 | 14 | 131.7 |
| P01859 | IGHG2 | IGHG2_HUMAN | Ig gamma-2 chain C region | 16.26 | 2 | 7 | 68.3 |
| P01743 |  | HV102_HUMAN | Ig heavy chain V-I region HG3 | 5.98 | 1 | 2 | 22.6 |
| P01777 |  | HV316_HUMAN | Ig heavy chain V-III region TEI | 15.97 | 1 | 4 | 49.7 |
| P01834 | IGKC | IGKC_HUMAN | Ig kappa chain C region | 66.98 | 4 | 22 | 293.6 |
| P01619 |  | KV301_HUMAN | Ig kappa chain V-III region B6 | 16.67 | 1 | 2 | 24.4 |
| P01625 |  | KV402_HUMAN | Ig kappa chain V-IV region Len | 15.79 | 1 | 1 | 15.8 |
| P80748 |  | LV302_HUMAN | Ig lambda chain V-III region LOI | 7.21 | 1 | 1 | 10.7 |
| P0CG04 | IGLC1 | LAC1_HUMAN | Ig lambda-1 chain C regions | 63.2 | 4 | - | 169.4 |
| P0CG05 | IGLC2 | LAC2_HUMAN | Ig lambda-2 chain C regions | 81.13 | 4 | 24 | 239 |
| A0M8Q6 | IGLC7 | LAC7_HUMAN | Ig lambda-7 chain C region | 38.68 | 1 | 12 | 148.9 |
| P01871 | IGHM | IGHM_HUMAN | Ig mu chain C region | 14.16 | 5 | 21 | 223.2 |
| Q14623 | IHH | IHH_HUMAN | Indian hedgehog protein | 13.38 | 3 | 7 | 76.6 |
| P35858 | IGFALS | ALS_HUMAN | Insulin-like growth factor-binding protein complex acid labile subunit | 4.13 | 2 | 3 | 39.3 |
| P17301 | ITGA2 | ITA2_HUMAN | Integrin alpha-2 | 1.86 | 1 | 3 | 50.5 |
| P05556 | ITGB1 | ITB1_HUMAN | Integrin beta-1 | 4.01 | 4 | 5 | 41.9 |
| P05106 | ITGB3 | ITB3_HUMAN | Integrin beta-3 | 1.4 | 1 | 1 | 10.3 |
| P19827 | ITIH1 | ITIH1_HUMAN | Inter-alpha-trypsin inhibitor heavy chain H1 | 1.43 | 1 | 1 | 10.7 |
| Q14624 | ITIH4 | ITIH4_HUMAN | Inter-alpha-trypsin inhibitor heavy chain H4 | 7.2 | 4 | 8 | 82.4 |
| P29622 | SERPINA4 | KAIN_HUMAN | Kallistatin | 7.73 | 2 | 4 | 67.7 |
| P01042 | KNG1 | KNG1_HUMAN | Kininogen-1 | 4.97 | 3 | 5 | 61.9 |
| P02750 | LRG1 | A2GL_HUMAN | Leucine-rich alpha-2-glycoprotein | 2.59 | 1 | 2 | 29.8 |
| P18428 | LBP | LBP_HUMAN | Lipopolysaccharide-binding protein | 10.2 | 4 | - | 93.7 |
| P51884 | LUM | LUM_HUMAN | Lumican | 14.2 | 4 | - | 70.6 |
| P61626 | LYZ | LYSC_HUMAN | Lysozyme C | 14.19 | 2 | 9 | 93.6 |
| Q9H8L6 | MMRN2 | MMRN2_HUMAN | Multimerin-2 | 0.95 | 1 | 3 | 37 |
| Q96PD5 | PGLYRP2 | PGRP2_HUMAN | N-acetylmuramoyl-L-alanine amidase | 16.32 | 4 | 11 | 115.3 |
| P04180 | LCAT | LCAT_HUMAN | Phosphatidylcholine-sterol acyltransferase | 18.64 | 7 | 42 | 353.4 |
| P80108 | GPLD1 | PHLD_HUMAN | Phosphatidylinositol-glycan-specific phospholipase D | 5 | 4 | 11 | 137.5 |
| P55058 | PLTP | PLTP_HUMAN | Phospholipid transfer protein | 25.35 | 9 | 36 | 295.3 |
| P36955 | SERPINF1 | PEDF_HUMAN | Pigment epithelium-derived factor | 37.32 | 14 | 54 | 536.6 |
| P05155 | SERPING1 | IC1_HUMAN | Plasma protease C1 inhibitor | 7.8 | 3 | 5 | 63.1 |
| P05154 | SERPINA5 | IPSP_HUMAN | Plasma serine protease inhibitor | 2.71 | 1 | 2 | 30.3 |
| P02775 | PPBP | CXCL7_HUMAN | Platelet basic protein | 32.81 | 3 | 13 | 131.4 |
| P02776 | PF4 | PLF4_HUMAN | Platelet factor 4 | 19.8 | 2 | 3 | 36.5 |
| Q13093 | PLA2G7 | PAFA_HUMAN | Platelet-activating factor acetylhydrolase | 12.47 | 3 | 13 | 157.4 |
| Q9UHG3 | PCYOX1 | PCYOX_HUMAN | Prenylcysteine oxidase 1 | 20.2 | 8 | 25 | 232.3 |
| P41222 | PTGDS | PTGDS_HUMAN | Prostaglandin-H2 D-isomerase | 12.11 | 2 | 4 | 40.5 |
| P02760 | AMBP | AMBP_HUMAN | Protein AMBP | 19.6 | 6 | 19 | 222.9 |
| Q9UK55 | SERPINA10 | ZPI_HUMAN | Protein Z-dependent protease inhibitor | 5.41 | 2 | 5 | 69.7 |
| P00734 | F2 | THRB_HUMAN | Prothrombin | 9.9 | 3 | - | 82.9 |
| P07988 | SFTPB | PSPB_HUMAN | Pulmonary surfactant-associated protein B | 8.14 | 1 | 2 | 33 |
| P02753 | RBP4 | RET4_HUMAN | Retinol-binding protein 4 | 29.85 | 4 | 20 | 119.3 |
| P02787 | TF | TRFE_HUMAN | Serotransferrin | 6.16 | 4 | 4 | 42.7 |
| P02768 | ALB | ALBU_HUMAN | Serum albumin | 66.83 | 45 | 970 | 9096.3 |
| P0DJI8 | SAA1 | SAA1_HUMAN | Serum amyloid A protein | 46.72 | 5 | 44 | 536.9 |
| P35542 | SAA4 | SAA4_HUMAN | Serum amyloid A-4 protein | 39.23 | 6 | 40 | 367.9 |
| P02743 | APCS | SAMP_HUMAN | Serum amyloid P-component | 13.9 | 2 | - | 60.6 |
| P27169 | PON1 | PON1_HUMAN | Serum paraoxonase/arylesterase 1 | 79.44 | 18 | 191 | 1652.3 |
| Q15166 | PON3 | PON3_HUMAN | Serum paraoxonase/lactonase 3 | 25.42 | 6 | 48 | 379.5 |
| P05452 | CLEC3B | TETN_HUMAN | Tetranectin | 16.34 | 2 | 2 | 23.3 |
| P19971 | TYMP | TYPH_HUMAN | Thymidine phosphorylase | 3.11 | 1 | 1 | 16 |
| P02766 | TTR | TTHY_HUMAN | Transthyretin | 34.01 | 5 | 67 | 683.8 |
| Q9BUN1 | MENT | CA056_HUMAN | Uncharacterized protein C1orf56 | 8.5 | 2 | 5 | 50.6 |
| P02774 | GC | VTDB_HUMAN | Vitamin D-binding protein | 35.02 | 11 | 46 | 434.3 |
| P04004 | VTN | VTNC_HUMAN | Vitronectin | 9.62 | 4 | 46 | 440.8 |
| P25311 | AZGP1 | ZA2G_HUMAN | Zinc-alpha-2-glycoprotein | 16.44 | 3 | 10 | 105.7 |

A total of 127 proteins were identified. Of those, 80 appeared in at least 3 different MS studies (red), 32 appeared in at least 1 different MS study (green) and 15 were newly described (black). UniProt accession number, entry name and gene symbol information were from UniProt database (http://www.uniprot.org/).
