# Supplementary material for: Impact of Virgin Olive Oil and Phenol-Enriched Virgin Olive Oils on the HDL Proteome in Hypercholesterolemic Subjects: A Double Blind, Randomized, Controlled, Cross-Over Clinical Trial (VOHF Study)
Source: PLoS One. 2015 Jun 10;10(6):e0129160. doi: 10.1371/journal.pone.0129160 (PMC4465699; doi:10.1371/journal.pone.0129160)
Supplement: S5 Table — (DOCX) [file pone.0129160.s009.docx]

**Supporting Information Table S5.** Proteins differentially expressed after each VOO intervention.

| **UNIPROT ACCESSION NUMBER** | **GENE SYMBOL** | **ENTRY NAME** | **PROTEIN NAME** | **PRINCIPAL BIOLOGICAL FUNCTION** | **FOLD CHANGE** |
| --- | --- | --- | --- | --- | --- |
| **VOO** | | | | | |
| **UP-REGULATED** |  |  |  |  |  |
| P02654 | APOC1 | APOC1_HUMAN | Apolipoprotein C-I | Cholesterol homeostasis | 2.96 |
| P02647 | APOA1 | APOA1_HUMAN | Apolipoprotein A-I | Cholesterol homeostasis | 1.69 |
| P11597 | CETP | CETP_HUMAN | Cholesteryl ester transfer protein | Cholesterol homeostasis | 1.58 |
| Q15166 | PON3 | PON3_HUMAN | Serum paraoxonase/lactonase 3 | Antioxidant protection | 1.56 |
| P04196 | HRG | HRG_HUMAN | Histidine-rich glycoprotein | Blood coagulation | 1.55 |
| P01743 |  | HV102_HUMAN | Ig heavy chain V-I region HG3 | Innate immune response | 1.55 |
| P02652 | APOA2 | APOA2_HUMAN | Apolipoprotein A-II | Cholesterol homeostasis | 1.45 |
| P0CG04 | IGLC1 | LAC1_HUMAN | Ig lambda-1 chain C regions | Innate immune response | 1.37 |
| Q14624 | ITIH4 | ITIH4_HUMAN | Inter-alpha-trypsin inhibitor heavy chain H4 | Transport | 1.37 |
| P06727 | APOA4 | APOA4_HUMAN | Apolipoprotein A-IV | Cholesterol homeostasis and Antioxidant protection | 1.36 |
| P19827 | ITIH1 | ITIH1_HUMAN | Inter-alpha-trypsin inhibitor heavy chain H1 | Transport | 1.36 |
| Q13790 | APOF | APOF_HUMAN | Apolipoprotein F | Cholesterol homeostasis | 1.34 |
| P01871 | IGHM | IGHM_HUMAN | Ig mu chain C region | Innate immune response | 1.32 |
| P81605 | DCD | DCD_HUMAN | Dermcidin | Defense response | 1.31 |
| P01042 | KNG1 | KNG1_HUMAN | Kininogen-1 | Blood coagulation | 1.30 |
| P05546 | SERPIND | HEP2_HUMAN | Heparin cofactor 2 | Blood coagulation | 1.27 |
| P10412 | HIST1H1E | H14_HUMAN | Histone H1.4 | nucleosome assembly | 1.27 |
| P01023 | A2M | A2MG_HUMAN | Alpha-2-macroglobulin | Blood coagulation | 1.25 |
| P02730 | SLC4A1 | B3AT_HUMAN | Band 3 anion transport protein | Transport | 1.25 |
| **DOWN-REGULATED** |  |  |  |  |  |
| P80748 |  | LV302_HUMAN | Ig lambda chain V-III region LOI | Innate immune response | 0.47 |
| P00738 | HP | HPT_HUMAN | Haptoglobin | Acute-phase response | 0.56 |
| P02776 | PF4 | PLF4_HUMAN | Platelet factor 4 | Blood coagulation | 0.56 |
| P30464 | HLAB | 1B15_HUMAN | HLA class I histocompatibility antigen. B-15 alpha chain | Immune response | 0.56 |
| P10909 | CLU | CLUS_HUMAN | Clusterin | Complement pathway and innate immune response | 0.61 |
| P05090 | APOD | APOD_HUMAN | Apolipoprotein D | Lipid transport | 0.61 |
| P16070 | CD44 | CD44_HUMAN | CD44 antigen | Cell adhesion | 0.64 |
| P69905 | HBA1 | HBA_HUMAN | Hemoglobin subunit alpha | Transport | 0.65 |
| P25311 | AZGP1 | ZA2G_HUMAN | Zinc-alpha-2-glycoprotein | Immune response | 0.69 |
| P0DJI8 | SAA1 | SAA_HUMAN | Serum amyloid A protein | Acute-phase response | 0.74 |
| P21333 | FLNA | FLNA_HUMAN | Filamin-A | Binding | 0.74 |
| [P02763](http://www.uniprot.org/uniprot/P02763) | ORM1 | A1AG1_HUMAN | Alpha-1-acid glycoprotein 1 | Acute-phase response | 0.75 |
| P08697 | SERPINF2 | A2AP_HUMAN | Alpha-2-antiplasmin | Acute-phase response | 0.75 |
| P01008 | SERPINC1 | ANT3_HUMAN | Antithrombin-III | Blood coagulation | 0.75 |
| Q9HDC9 | APMAP | APMAP_HUMAN | Adipocyte plasma membrane-associated protein | Membrane protein | 0.76 |
| P02753 | RBP4 | RET4_HUMAN | Retinol-binding protein 4 | Transport | 0.78 |
| Q96PD5 | PGLYRP2 | PGRP2_HUMAN | N-acetylmuramoyl-L-alanine amidase | Innate immune response | 0.79 |
| O14791 | APOL1 | APOL1_HUMAN | Apolipoprotein L1 | Lipid transport | 0.79 |
| P15144 | ANPEP | AMPN_HUMAN | Aminopeptidase N | Proteolysis | 0.80 |
| P68871 | HBB | HBB_HUMAN | Hemoglobin subunit beta | Transport | 0.81 |
| Q96KN2 | CNDP1 | CNDP1_HUMAN | Beta-Ala-His dipeptidase | Proteolysis | 0.82 |
| P27105 | STOM | STOM_HUMAN | Erythrocyte band 7 integral membrane protein | Membrane protein | 0.82 |
| P02671 | FGG | FIBG_HUMAN | Fibrinogen gamma chain | Blood coagulation | 0.83 |
| O95445 | APOM | APOM_HUMAN | Apolipoprotein M | Cholesterol homeostasis | 0.83 |
| [P04217](http://www.uniprot.org/uniprot/P04217) | A1BG | A1BG_HUMAN | Alpha-1B-glycoprotein | Extracellular region | 0.83 |
| P02765 | AHSG | FETUA_HUMAN | Alpha-2-HS-glycoprotein | Acute-phase response | 0.84 |
| P35542 | SAA4 | SAA4_HUMAN | Serum amyloid A-4 protein | Acute-phase response | 0.84 |
| P02656 | APOC3 | APOC3_HUMAN | Apolipoprotein C-III | Cholesterol homeostasis | 0.84 |
| P04439 | HLAA | 1A03_HUMAN | HLA class I histocompatibility antigen. A-3 alpha chain | Immune response | 0.84 |
| Q8TDL5 | bpifb1 | BPIB1_HUMAN | BPI fold-containing family B member 1 | Lipid binding | 0.84 |
| **FVOO** | | | | | |
| **UP-REGULATED** |  |  |  |  |  |
| P02775 | PPBP | CXCL7_HUMAN | Platelet basic protein | Blood coagulation | 1.77 |
| Q15166 | PON3 | PON3_HUMAN | Serum paraoxonase/lactonase 3 | Antioxidant protection | 1.55 |
| P01871 | IGHM | IGHM_HUMAN | Ig mu chain C region | Innate immune response | 1.54 |
| P01743 |  | HV102_HUMAN | Ig heavy chain V-I region HG3 | Innate immune response | 1.47 |
| P02652 | APOA2 | APOA2_HUMAN | Apolipoprotein A-II | Cholesterol homeostasis | 1.40 |
| P04196 | HRG | HRG_HUMAN | Histidine-rich glycoprotein | Blood coagulation | 1.39 |
| P02647 | APOA1 | APOA1_HUMAN | Apolipoprotein A-I | Cholesterol homeostasis | 1.37 |
| P01023 | A2M | A2MG_HUMAN | Alpha-2-macroglobulin | Blood coagulation | 1.33 |
| P0CG04 | IGLC1 | LAC1_HUMAN | Ig lambda-1 chain C regions | Innate immune response | 1.32 |
| Q9NNX6 | CD209 | CD209_HUMAN | CD209 antigen | Innate immune response | 1.32 |
| P19827 | ITIH1 | ITIH1_HUMAN | Inter-alpha-trypsin inhibitor heavy chain H1 | Transport | 1.32 |
| P11597 | CETP | CETP_HUMAN | Cholesteryl ester transfer protein | Cholesterol homeostasis | 1.30 |
| P01042 | KNG1 | KNG1_HUMAN | Kininogen-1 | Blood coagulation | 1.29 |
| P08519 | LPA | APOA_HUMAN | Apolipoprotein(a) | Lipid transport | 1.28 |
| P05546 | SERPIND1 | HEP2_HUMAN | Heparin cofactor 2 | Blood coagulation | 1.27 |
| P0DJI8 | SAA1 | SAA_HUMAN | Serum amyloid A protein | Acute-phase response | 1.26 |
| P0CG05 | IGLC2 | LAC2_HUMAN | Ig lambda-2 chain C regions | Innate immune response | 1.26 |
| **DOWN-REGULATED** |  |  |  |  |  |
| [P02763](http://www.uniprot.org/uniprot/P02763) | ORM1 | A1AG1_HUMAN | Alpha-1-acid glycoprotein 1 | Acute-phase response | 0.53 |
| P81605 | DCD | DCD_HUMAN | Dermcidin | Defense response | 0.53 |
| Q96KN2 | CNDP1 | CNDP1_HUMAN | Beta-Ala-His dipeptidase | Proteolysis | 0.69 |
| P05090 | APOD | APOD_HUMAN | Apolipoprotein D | Lipid transport | 0.71 |
| P05154 | SERPINA5 | IPSP_HUMAN | Plasma serine protease inhibitor | Lipid transport | 0.71 |
| Q9BUN1 | MENT | CA056_HUMAN | Uncharacterized protein C1orf56 | Extracellular region | 0.71 |
| P08697 | SERPINF2 | A2AP_HUMAN | Alpha-2-antiplasmin | Acute-phase response | 0.72 |
| O14791 | APOL1 | APOL1_HUMAN | Apolipoprotein L1 | Lipid transport | 0.72 |
| P69905 | HBA1 | HBA_HUMAN | Hemoglobin subunit alpha | Transport | 0.72 |
| P25311 | AZGP1 | ZA2G_HUMAN | Zinc-alpha-2-glycoprotein | Immune response | 0.75 |
| [P19652](http://www.uniprot.org/uniprot/P19652) | ORM2 | A1AG2_HUMAN | Alpha-1-acid glycoprotein 2 | Acute-phase response | 0.76 |
| P10909 | CLU | CLUS_HUMAN | Clusterin | Complement pathway and innate immune response | 0.78 |
| P15144 | ANPEP | AMPN_HUMAN | Aminopeptidase N | Proteolysis | 0.78 |
| P02753 | RBP4 | RET4_HUMAN | Retinol-binding protein 4 | Transport | 0.78 |
| P02671 | FGA | FIBA_HUMAN | Fibrinogen alpha chain | Blood coagulation | 0.79 |
| Q9HDC9 | APMAP | APMAP_HUMAN | Adipocyte plasma membrane-associated protein | Membrane protein | 0.79 |
| P02765 | AHSG | FETUA_HUMAN | Alpha-2-HS-glycoprotein | Acute-phase response | 0.80 |
| P00738 | HP | HPT_HUMAN | Haptoglobin | Acute-phase response | 0.80 |
| P01008 | SERPINC1 | ANT3_HUMAN | Antithrombin-III | Blood coagulation | 0.80 |
| Q9BQE5 | APOL2 | APOL2_HUMAN | Apolipoprotein L2 | Lipid transport | 0.81 |
| P35858 | IGFALS | ALS_HUMAN | Insulin-like growth factor-binding protein complex acid labile subunit | cell adhesion and signalling | 0.82 |
| P02671 | FGG | FIBG_HUMAN | Fibrinogen gamma chain | Blood coagulation | 0.83 |
| P04180 | LCAT | LCAT_HUMAN | Phosphatidylcholine-sterol acyltransferase | Cholesterol homeostasis | 0.83 |
| **FVOOT** | | | | | |
| **UP-REGULATED** |  |  |  |  |  |
| P43652 | AFM | AFAM_HUMAN | Afamin | Transport | 2.38 |
| P02655 | APOC2 | APOC2_HUMAN | Apolipoprotein C-II | Cholesterol homeostasis | 1.78 |
| P05106 | ITGB3 | ITB3_HUMAN | Integrin beta-3 | Cell adhesion | 1.74 |
| P02656 | APOC3 | APOC3_HUMAN | Apolipoprotein C-III | Cholesterol homeostasis | 1.70 |
| P02647 | APOA1 | APOA1_HUMAN | Apolipoprotein A-I | Cholesterol homeostasis | 1.60 |
| P06727 | APOA4 | APOA4_HUMAN | Apolipoprotein A-IV | Cholesterol homeostasis and Antioxidant protection | 1.52 |
| P35542 | SAA4 | SAA4_HUMAN | Serum amyloid A-4 protein | Acute-phase response | 1.51 |
| P02774 | GC | VTDB_HUMAN | Vitamin D-binding protein | Transport | 1.49 |
| P0DJI8 | SAA1 | SAA_HUMAN | Serum amyloid A protein | Acute-phase response | 1.47 |
| P02775 | PPBP | CXCL7_HUMAN | Platelet basic protein | Blood coagulation | 1.41 |
| Q15166 | PON3 | PON3_HUMAN | Serum paraoxonase/lactonase 3 | Antioxidant protection | 1.39 |
| P01023 | A2M | A2MG_HUMAN | Alpha-2-macroglobulin | Blood coagulation | 1.39 |
| P01743 |  | HV102_HUMAN | Ig heavy chain V-I region HG3 | Innate immune response | 1.39 |
| P68871 | HBB | HBB_HUMAN | Hemoglobin subunit beta | Transport | 1.37 |
| P02652 | APOA2 | APOA2_HUMAN | Apolipoprotein A-II | Cholesterol homeostasis | 1.34 |
| P01859 | IGHG2 | IGHG2_HUMAN | Ig gamma-2 chain C region O | Innate immune response | 1.34 |
| P05546 | SERPIND1 | HEP2_HUMAN | Heparin cofactor 2 | Blood coagulation | 1.32 |
| P02654 | APOC1 | APOC1_HUMAN | Apolipoprotein C-I | Cholesterol homeostasis | 1.25 |
| **DOWN-REGULATED** |  |  |  |  |  |
| P30464 | HLAB | 1B15_HUMAN | HLA class I histocompatibility antigen. B-15 alpha chain | Immune response | 0.53 |
| [P02763](http://www.uniprot.org/uniprot/P02763) | ORM1 | A1AG1_HUMAN | Alpha-1-acid glycoprotein 1 | Acute-phase response | 0.58 |
| P25311 | AZGP1 | ZA2G_HUMAN | Zinc-alpha-2-glycoprotein | Immune response | 0.63 |
| P02776 | PF4 | PLF4_HUMAN | Platelet factor 4 | Blood coagulation | 0.64 |
| P10909 | CLU | CLUS_HUMAN | Clusterin | Complement pathway and innate immune response | 0.64 |
| P00738 | HP | HPT_HUMAN | Haptoglobin | Acute-phase response | 0.65 |
| P05090 | APOD | APOD_HUMAN | Apolipoprotein D | Lipid transport | 0.68 |
| P15144 | ANPEP | AMPN_HUMAN | Aminopeptidase N | Proteolysis | 0.68 |
| P16070 | CD44 | CD44_HUMAN | CD44 antigen | Cell adhesion | 0.69 |
| P05154 | SERPINA5 | IPSP_HUMAN | Plasma serine protease inhibitor | Lipid transport | 0.72 |
| Q96KN2 | CNDP1 | CNDP1_HUMAN | Beta-Ala-His dipeptidase | Proteolysis | 0.72 |
| P21333 | FLNA | FLNA_HUMAN | Filamin-A | Binding | 0.75 |
| P02765 | AHSG | FETUA_HUMAN | Alpha-2-HS-glycoprotein | Acute-phase response | 0.76 |
| P08697 | SERPINF2 | A2AP_HUMAN | Alpha-2-antiplasmin | Acute-phase response | 0.77 |
| P02753 | RBP4 | RET4_HUMAN | Retinol-binding protein 4 | Transport | 0.77 |
| Q9HDC9 | APMAP | APMAP_HUMAN | Adipocyte plasma membrane-associated protein | Membrane protein | 0.80 |
| P04439 | HLAA | 1A03_HUMAN | HLA class I histocompatibility antigen. A-3 alpha chain | Immune response | 0.84 |

Fold change > 1.3 denotes proteins up-regulated while fold change < 0.8 denotes decrease protein expression after the VOO supplementations relative to the control-baseline. UniProt accession number, entry name, gene symbol and principal biological function information were from UniProt database (http://www.uniprot.org/).
